# Selective titin cleavage disrupts cardiac mechanical homeostasis to drive heart failure and fibrosis

**DOI:** 10.1038/s44161-026-00829-z

**Published:** 2026-06-16

**Authors:** Johanna K. Freundt, Paulina Hartmann, Christine M. Loescher, Andreas Unger, Franziska Koser, Annika J. Klotz, Lena Wildschütz, Lydia Wachsmuth, Susanne Hille, Michaela M. Door, Anne Helfen, Richard Holtmeier, Cornelius Faber, Jonathan A. Kirk, Verena Hoerr, Oliver J. Müller, Wolfgang A. Linke

**Affiliations:** 1 Institute of Physiology II, University of Münster, Münster, Germany; 2 Clinic of Radiology, University of Münster, Münster, Germany; 3 Department of Internal Medicine V, University of Kiel, Kiel, Germany; 4 Section of Cardiology, Department of Medicine, University of Chicago Biological Sciences Division, Chicago, IL USA; 5 German Centre for Cardiovascular Research, Partner Site North, Kiel, Germany; 6 Department of Cardiology and Pneumology, University Medicine Göttingen, Göttingen, Germany; 7 German Centre for Cardiovascular Research, Partner Site Lower Saxony, Göttingen, Germany

**Keywords:** Cardiovascular diseases, Heart failure

## Abstract

Titin, the largest human protein, forms the elastic sarcomeric backbone, providing passive stiffness and length-dependent activation in cardiomyocytes. Whereas titin mutations cause inherited cardiomyopathies, ischemic and chemotherapy-induced injury also provoke proteolytic cleavage of titin’s elastic segment. However, the effects of acute titin stiffness loss remain unknown. Here we develop a knock-in mouse enabling in vivo cleavage of cardiac titin springs and use multimodal analysis (cardiac magnetic resonance imaging, echocardiography, microscopy, omics) to show that titin cleavage does not dilate the heart but reduces chamber size and impairs ventricular filling. Mechanical assays of isolated cardiomyocytes reveal diminished restoring forces causing a loss of elastic recoil. In vivo cleavage disrupts junctions, including integrin linkages and connexin 43 gap junctions, widens intermyocyte space without hypertrophy or hyperplasia and drives fibroblast activation, extracellular matrix remodeling and fibrosis. Compensatory mechanisms fail, leading to decompensated heart failure. These findings establish that proteolytic titin cleavage perturbs cardiac mechanical homeostasis, driving disease and matrix stiffening.

## Abstract (web summary)

Freundt, Hartmann and colleagues use a multimodal approach to show that titin cleavage impairs ventricular filling and reduces chamber size by disrupting elastic recoil, promotes fibroblast activation and ultimately drives decompensated heart failure, supporting a function of titin as a mechanical stress sensor.

## Main

Cardiac mechanical function is driven by sarcomeres, the terminal effectors of excitation–contraction coupling. The structural integrity of the sarcomere critically depends on the giant protein titin, which spans half a sarcomere from the Z-disk to the M-band and serves as a molecular template for the highly ordered assembly of myosin filaments in striated muscle^1–4^. Within its I-band region, titin contains an elastic segment that governs myocardial passive stiffness, contributes to viscoelasticity and modulates contractile properties^5–9^. Although pathological perturbations of titin function are common, their in vivo impact on cardiac performance remains incompletely understood. Truncating variants in *TTN* underlie numerous inherited cardiomyopathies^10^ and considerably increase cardiomyopathy risk^11^. In addition, alterations in titin, whether through shifts in isoform expression (N2BA:N2B)^12–14^ or through posttranslational modifications^15^, finely tune cardiomyocyte passive stiffness but, when dysregulated, promote heart disease^5,6,15^.

Equally relevant, yet less explored, is the increased proteolytic cleavage of titin’s elastic I-band by calpains and matrix metalloproteinases (MMPs) under common disease conditions, including atrial fibrillation^16^, postischemic injury^17,18^ and chemotherapy-induced cardiotoxicity^19,20^. Notably, an ‘enigmatic’ proteolytic cleavage site resides in the mid-I-band titin region and gives rise to the appearance of ‘T2’ titin on loose gels. T2 titin comprises the A-band and adjacent spring fragment and accounts for approximately 12–14% of total titin protein in endstage failing human hearts^21^. T2 titin is thought to arise from calpain- and MMP-mediated cleavage and has been proposed to reflect sarcomere turnover and repair^16,17^. However, the (patho)physiological relevance of T2 titin and its Z-disk-anchored counterpart, particularly with respect to titin’s mechanical function, remains poorly understood. Dissecting the in vivo mechanical consequences of titin-spring cleavage remains challenging, as it requires the precise disruption of the elastic element without compromising sarcomeric scaffolding, unlike existing deletion models^2,22^.

Beyond intrinsic cardiomyocyte mechanics, proteolytic titin cleavage (TC) may have broader consequences for the interplay among cardiomyocytes, the extracellular matrix (ECM), and fibroblasts, which is central to the pathogenesis of heart failure (HF). Fibroblasts sense mechanical cues, including altered substrate stiffness or stretch^23,24^, and, upon activation into myofibroblasts, drive collagen deposition that stiffens the myocardium^25^. This mechanosensing also involves the remodeling of cardiomyocyte–ECM–fibroblast contacts through changes in the expression and organization of integrin complexes (such as fibronectin-binding α5β1)^26–28^. Whether fibroblasts can directly detect pathological alterations in cardiomyocyte force to initiate ECM remodeling is an emerging concept^29,30^. However, it remains unknown whether the acute loss of titin-based passive force, such as through proteolytic cleavage, can trigger fibroblast activation and fibrosis, or how such changes affect ventricular function. Recent evidence suggests that protease activation in cardiomyocytes initiates reactive fibrosis and a subsequent decline in cardiac performance^17,20,31^, but the specific contribution of TC to this process has not been defined. These knowledge gaps may hinder the development of therapies aimed at preventing maladaptive structural remodeling in diseased hearts. To address this, we generated a knock-in mouse that permits the targeted in vivo cleavage of titin springs, enabling the direct interrogation of how stiffness loss from a single cleavage event affects ventricular mechanics, intercellular integrity and ECM properties.

## Results

### Cleavage of elastic titin in mouse heart

**Fig. 1:**
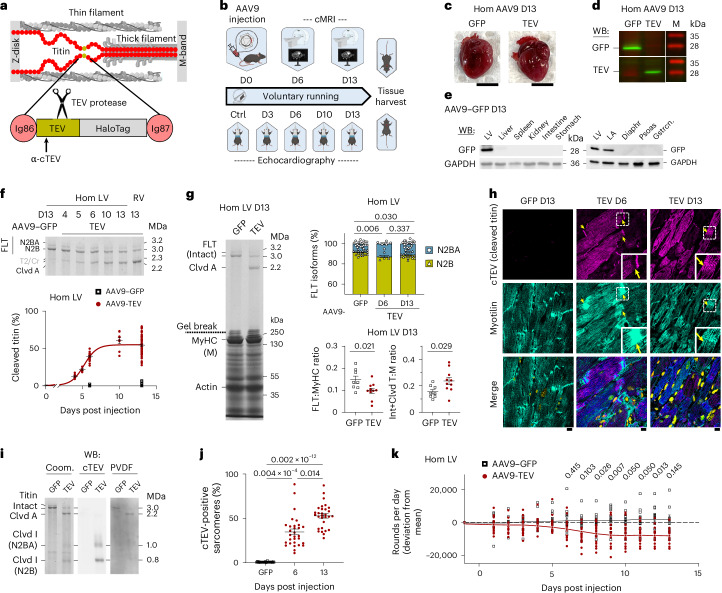
In vivo TC model. **a**, A schematic of a TC mouse half sarcomere with a TEV protease recognition site in titin’s I-band; the arrow indicates the α-cTEV-detected fragment. **b**, The experimental timeline. Hom/Het TC mice received AAV9-TEV or AAV9–GFP at D0; daily running was recorded; echocardiography and MRI were performed at select time points; and tissues were harvested mainly at D6 and D13. **c**, Representative D13 hearts from Hom mice (GFP/TEV). Scale bars, 5 mm. **d**, A WB of Hom TC LV at D13 showing GFP and TEV overexpression, respectively (*n* = 3 biological replicates). **e**, A WB of GFP across AAV9–GFP-injected tissues at D13 (LA, left atrium; Diaphr, diaphragm; Gstrcn., gastrocnemius muscle), with GAPDH as the loading control. See Extended Data Fig. 1a for a summary analysis. **f**, Top: the Coomassie (Coom.)-stained titin gel of Hom TC LV/RV showing progressive cleavage post TEV injection. N2BA/N2B, intact, full-length titin (FLT) isoforms; Clvd A, Cleaved A-band titin fragment; T2/Cr, proteolytic titin fragment/Cronos. Bottom: TC time course (GFP D0 *N* = 7 animals (*n* = 7 gel bands), D6 *N* = 6 (*n* = 6) and D13 *N* = 17 (*n* = 28); TEV D4 and D5 *N* = 2 (*n* = 6), D6 *N* = 6 (*n* = 23), D10 *N* = 2 (*n* = 8) and D13 *N* = 17 (*n* = 66). **g**, The representative, Coom.-stained, two-phase gel (left), used to determine the FLT (N2BA, N2B) isoform composition at D13 (top right): GFP *N* = 19; TEV D6 *N* = 6 and D13 *N* = 19 (one-way ANOVA or Tukey) and the intact FLT-to-myosin heavy chain (MyHC) (bottom left) and total (intact+cleaved (Int+Clvd)) titin-to-MyHC (bottom right) ratios (GFP *N* = 8, TEV *N* = 10; two-tailed, unpaired *t*-test). **h**, The confocal LV images: α-cTEV (Cy3), α-myotilin (AlexaFluor 488) and merge with DAPI (yellow); the arrows mark sarcomere–ICD detachment. Scale bars, 10 µm (insets: ROIs). **i**, The validation of cleavage in Hom LV at D13 by titin gel, α-cTEV WB and PVDF loading control. Clvd I, Cleaved I-band titin fragment. **j**, The quantification of titin-cleaved versus intact sarcomeres (~13,000–18,000 per image; GFP *N* = 2 and TEV *N* = 3; Kruskal–Wallis and Dunn) on anti-cTEV-stained IF images. **k**, The voluntary running deviation from baseline (laps/day; GFP D1, D5 and D7 *N* = 10; GFP D2 and D6 *N* = 8; GFP D3, D4 and D13 *N* = 9; GFP D8–D12 *N* = 11; TEV D1 *N* = 17; TEV D2 *N* = 13; TEV D3 and D10–D13 *N* = 16; TEV D4 *N* = 15; and TEV D5–D9 *N* = 18; two-way ANOVA (mixed-effects model for repeated measures or Šidák)). Data are mean ± s.e.m. Schematics created in BioRender: **a**, Linke, W. https://biorender.com/fse1x68 (2026); **b**, Linke, W. https://biorender.com/9lme2xm (2026). Source data

We hypothesized that selective cleavage of elastic titin near the native T2 cleavage site would recapitulate aspects of titin-related cardiomyopathy and elucidate the role of titin stiffness in maintaining cardiac mechanical balance. To test this, we employed the TC mouse model^32^, inserting a tobacco etch virus protease (TEV) recognition site into titin’s I-band region between *Ttn* exons 225 and 226, encoding immunoglobulin-like domains 86 and 87 (Fig. 1a). Heart-specific cleavage was induced via AAV9-mediated TEV expression under a cardiac troponin-T promoter, delivered by tail vein injections into homozygous (Hom) or heterozygous (Het) TC mice, with AAV9–GFP serving as a control. Cardiac function was monitored by magnetic resonance imaging (MRI) at day (D)6 and D13 post injection and by transthoracic echocardiography (TTE) at D0, D3, D6, D10 and D13 (Fig. 1b). Voluntary running activity was recorded throughout, and tissue samples were mainly collected at D6 and D13.

**Extended Data Fig. 1:**
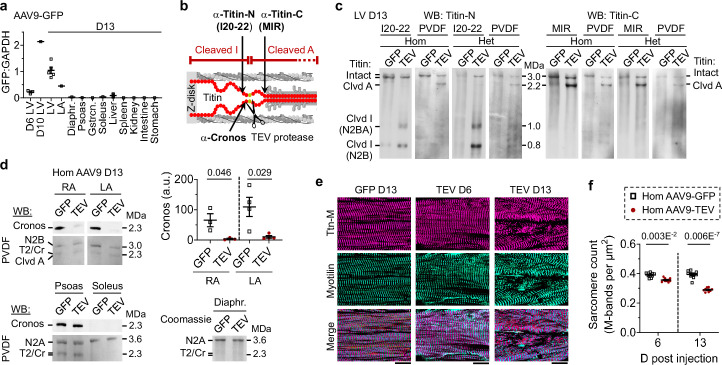
Additional cardiac protein analysis after in vivo TC. **a**, Results of Western blot (WB) of GFP expression in organs from AAV9-GFP-injected mice, normalized to GAPDH: D6 left ventricle (LV; N = 3 mice), D10 LV (N = 1), D13 LV (N = 5), and D13 left atrium (LA), liver, diaphragm, psoas, gastrocnemius, soleus, spleen, kidney, intestine, and stomach (N = 1 each). **b**, Schematic of TEV-induced TC within the half-sarcomere, showing cleaved I-band and A-band fragments and the binding sites for anti-titin antibodies (I20-22, MIR, Cronos). **c**, WB of LV tissue from Hom and Het TC mice at D13 post-injection confirms in vivo TC: *left*, N-terminal epitope (I20-22); *right*, C-terminal epitope (MIR); PVDF staining as loading control. **d**, Cronos expression and cleavage in right atrium (RA) and LA of Hom TC mice at D13 after AAV9-GFP or AAV9-TEV; representative WBs, with PVDF control, and summary data (RA, N = 3 mice; LA, N = 4); lower panels show absence of cleavage in skeletal muscles by Cronos WB and Coomassie staining. **e**, Representative confocal images (out of >20 similar images) of Hom TC LV tissue: AAV9-GFP at D13 and AAV9-TEV at D6 or D13; *top*, titin C-terminus (anti-Ttn-M, Cy3); *middle*, Z-disks (anti-myotilin, AlexaFluor 488); *bottom*, merge. Scale bars, 10 µm. **f**, Sarcomere counts from Ttn-M staining (semi-quantitative NIS software; n = 10 images per group; GFP, N = 6; TEV, N = 3). Data are mean ± SEM; statistical significance by unpaired t-test. Schematic in **b** created in BioRender; Linke, W. https://biorender.com/om5t110 (2026). Source data

At D13, hearts from AAV9–GFP and AAV9-TEV-injected Hom TC mice displayed similar external morphology (Fig. 1c) and robust expression of GFP and TEV, respectively (Fig. 1d). Western blots (WBs) using tissue from GFP-injected mice confirmed cardiac-restricted expression: GFP was evident in all heart chambers yet nearly absent in skeletal muscle, spleen and even liver (Fig. 1e and Extended Data Fig. 1a). In TEV-injected Hom TC left ventricle (LV), TC, assessed on loose protein gels, increased from ~12% at D4 to ~40% at D6, plateauing at ~55–60% by D10–D13 (Fig. 1f); titin was also cleaved in the right ventricle (RV; Fig. 1f). No cleavage was observed in GFP-injected controls. Both major titin isoforms (N2BA and N2B) were cleaved to a similar extent, with only minor shifts in their relative abundance, indicating a modest preference for N2B (Fig. 1g). Cleaved titin levels exceeded the loss of intact titin, resulting in a net increase in total titin protein (Fig. 1g). TEV-cleaved titin appeared on WBs as an N-terminal fragment (Cleaved-I) and a C-terminal fragment (Cleaved-A) (Extended Data Fig. 1b,c); Cleaved-A appeared as a single band, whereas Cleaved-I split into two signals corresponding to N2BA and N2B (Extended Data Fig. 1c). The alternative titin isoform Cronos, abundantly expressed in the atria and fast skeletal muscles^2^, was cleaved at its extreme N-terminus, confirming cleavage in the atria but not in skeletal muscles (Extended Data Fig. 1d). Together, these data demonstrate the successful, cardiac-specific, in vivo cleavage of titin.

We next examined the spatial pattern of TC using immunofluorescence (IF) on LV sections from Hom TC mice. An antibody that recognizes the TEV-cleaved sequence (α-cTEV) revealed a mosaic pattern of titin-cleaved and uncleaved cardiomyocytes (Fig. 1h). On WBs, α-cTEV specifically detected the two Cleaved-I bands (Fig. 1i). Cleaved titin localized to both the sarcomere and the intercalated disk (ICD), where costaining with the Z-disk/ICD marker myotilin revealed ICD ‘streaming’, indicative of mechanical deformation (Fig. 1h, arrows), reflecting the disorganization of myotilin-binding cytoskeletal structures. The quantification of cTEV-positive signals showed ~35% cleaved sarcomeres at D6 and ~53% at D13, consistent with WB data, whereas GFP-injected hearts were negative (Fig. 1j). Furthermore, TEV-injected LV exhibited a reduction in sarcomere density by 8.9% at D6 and 26.4% at D13 compared with controls (Extended Data Fig. 1e,f). Functionally, TEV-injected Hom TC mice demonstrated reduced voluntary wheel running activity starting at D7 and D8 (Fig. 1k), prompting further detailed cardiac functional analyses.

### Restricted filling after TC

**Fig. 2:**
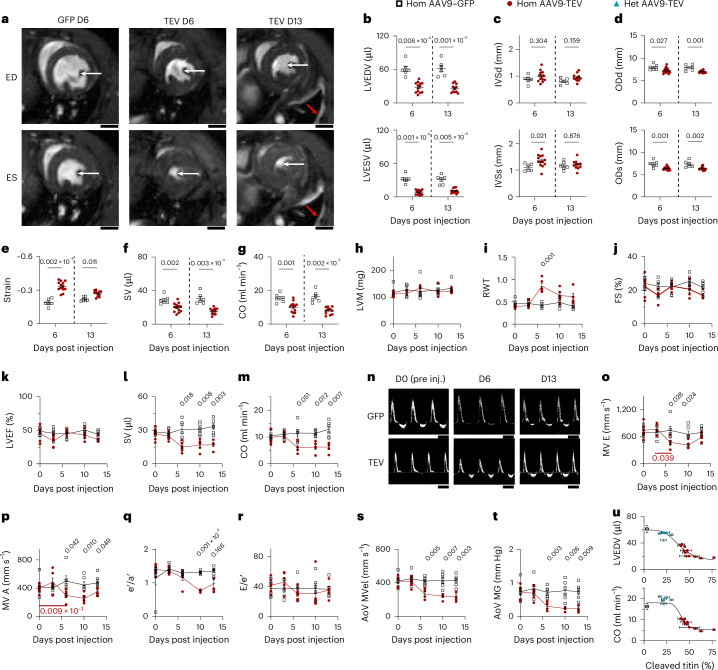
Cardiac structure and function in AAV9-TEV titin-cleaved and AAV9–GFP control mice. **a**, Representative SAX cMRI images (two-chamber view) at end-diastole (ED) and end-systole (ES) at D6 and D13 post AAV9 injection versus AAV9–GFP controls. LV volume (white arrows), IVS width and OD were quantified. Note pleural effusion (red arrows) at D13 post TEV injection. Scale bars, 2 mm. **b**–**g**, The cMRI-derived parameters: LVEDV and LVESV (**b**), IVS width at ED (IVSd) and IVS width at ES (IVSs) (**c**), OD at ED (ODd) and OD at ES (ODs) (**d**), Green–Lagrange strain (**e**), SV (**f**) and cardiac output (CO) (**g**). **h**–**m**, The M-mode TTE parameters: LV mass (LVM) (**h**), RWT (**i**), fractional shortening (FS) (**j**), LVEF (**k**), SV (**l**) and CO (**m**). **n**, Representative pulsed-wave Doppler measurements of MV flow. Scale bars, 500 mm s^−1^ (vertical), 0.1 s (horizontal). **o**,**p**, The pulsed-wave Doppler: MV E wave velocity (early filling) (**o**) and MV A wave velocity (late filling) (**p**). **q**,**r**, The tissue Doppler: e′/a′ ratio (**q**) and E/e′ ratio (**r**). **s**,**t**, Pulsed-wave Doppler measurements of aortic valve (AoV) flow: AoV mean velocity (MVel) (**s**) and AoV mean pressure gradient (MG) (**t**). **u**, The correlation of TC with LVEDV (top) and CO (bottom), across AAV9-injected Hom and Het mice; lines are sigmoidal fits to means; *N* = 6 (GFP, Het TEV), *N* = 13 (Hom TEV) (top), *N* = 6 (GFP, Het TEV) and *N* = 11 (Hom TEV) mice per group (bottom). cMRI in **b**–**g**, *N* = 6 (GFP), *N* = 13 (D6 TEV) and *N* = 11 (D13 TEV) mice per group; TTE in **h**–**t**, *N* = 6 (GFP), *N* = 6 (D0–6 TEV) and *N* = 5 (D10–D13 TEV) mice per group. Data are mean ± s.e.m. Statistical significance by two-way ANOVA (mixed-effects model for repeated measures), followed by Šidák’s or Tukey’s test. Source data

**Extended Data Fig. 2:**
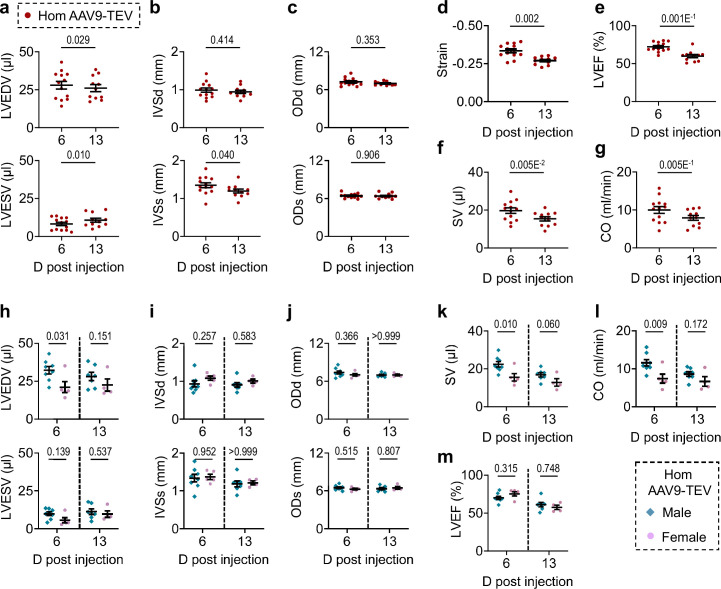
Additional cMRI analyses in AAV9-TEV-injected Hom TC mice. **a-g**, cMRI-derived parameters in AAV9-TEV D6 vs. D13: **a**, left ventricular (LV) volumes: end-diastolic (LVEDV) and end-systolic (LVESV); **b**, Interventricular septum (IVS) widths: diastolic (IVSd) and systolic (IVSs); **c**, Outer heart diameters (OD): diastolic (ODd) and systolic (ODs); and functional parameters: **d**, Green-Lagrange strain, **e**, LV ejection fraction (LVEF), **f**, stroke volume (SV), and **g**, cardiac output (CO). Data are mean ± SEM; N = 13 (D6), N = 11 (D13). Statistical significance by paired t-test. **h-m**, sex-stratified cMRI analyses: **h**, LVEDV and LVESV; **i**, IVSd and IVSs; **j**, ODd and ODs; and functional parameters: **k**, SV, **l**, CO, and **m**, LVEF. Data are mean ± SEM; N = 8 (D6, male), N = 7 (D13, male), N = 5 (D6, female), N = 4 (D13, female). Statistical significance by two-way ANOVA (mixed-effects analysis) followed by Šidák’s test. Source data

Time-resolved noninvasive imaging revealed that selective TC in TEV-injected versus GFP-injected Hom TC mice markedly shrinks the LV cavity size—an unexpected finding given the proposed role of titin stiffness in protecting against overstretch^5,6^, yet consistent with its predicted function in supporting elastic recoil^33,34^. Short-axis (SAX) cardiac MRI (cMRI) scans (Fig. 2a) demonstrated pronounced reductions in end-diastolic and end-systolic LV volumes at D6 and D13 (Fig. 2b); diastolic volume further decreased from D6 to D13, whereas systolic volume slightly increased by D13 (Extended Data Fig. 2a). The interventricular septum (IVS) was unchanged at end-diastole but thickened at end-systole at D6 (Fig. 2c), recovering by D13 (Extended Data Fig. 2b). End-diastolic and end-systolic outer heart diameters (OD) were modestly reduced (Fig. 2d) and similar at D6 and D13 (Extended Data Fig. 2c). The Green–Lagrange strain sharply increased at D6 and declined by D13 (Fig. 2e; Extended Data Fig. 2d). Under anesthesia, heart rate (~500 beats per minute) remained steady, whereas stroke volume (SV) and cardiac output decreased at D6 and declined further by D13, as did the otherwise only mildly affected LV ejection fraction (LVEF; Fig. 2f,g and Extended Data Fig. 2e–g). Notably, subtle sex-dependent differences emerged, with females exhibiting slightly greater reductions in LV end-diastolic volume, SV and cardiac output at D6 (Extended Data Fig. 2h–m). Cinematic long-axis cMRI scans (Supplementary Movies 1–3) revealed minimal post-systolic expansion in titin-cleaved hearts, impairing LV inflow across the mitral valve (MV). Pleural effusions frequently appeared at D13 (Fig. 2a), indicating severe backward failure.

**Extended Data Fig. 3:**
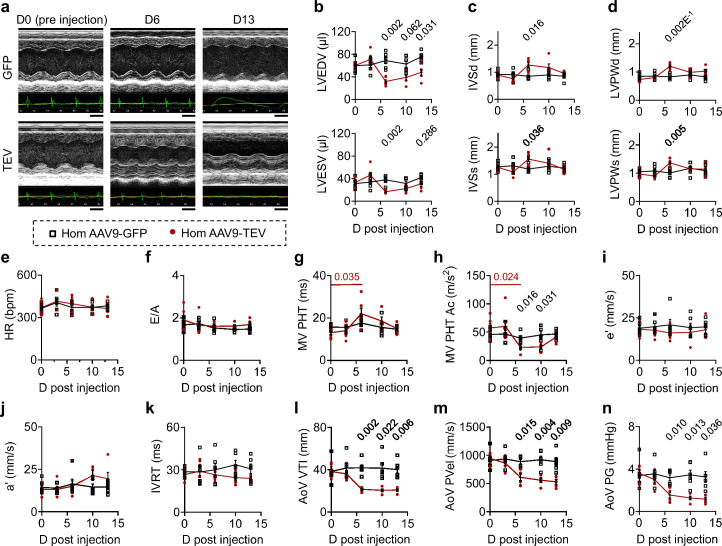
Additional transthoracic echocardiography analyses in AAV9-injected Hom TC mice. **a**, Representative M-mode echocardiography images used to quantify left ventricular (LV) volume, interventricular septum (IVS) width, and posterior wall (PW) thickness at four time points post-AAV9 injection (D0 = pre injection). Scale bars, 0.1 s. **b**, LV volumes: end-diastolic (LVEDV) and end-systolic (LVESV). **c**, IVS width: diastolic (IVSd) and systolic (IVSs). **d**, LV posterior wall: diastolic (LVPWd) and systolic (LVPWs). **e**, Heart rate (HR) under anesthesia. **f-h**, Pulsed-wave Doppler assessment of mitral valve (MV) flow: **f**, E/A ratio; **g**, MV pressure half time (PHT); **h**, MV PHT acceleration (Ac). **i-j**; Tissue Doppler imaging: **i**, e′ and **j**, a′ velocities. **k**, Isovolumic relaxation time (IVRT). **l-n**, Pulsed-wave Doppler measurements of aortic valve (AoV) flow: **l**, AoV velocity time integral (VTI); **m**, AoV peak velocity (PVel); and **n**, AoV pressure gradient (PG); Data are mean ± SEM; N = 6 (GFP, TEV D0-3); N = 5 (TEV D10-13) mice/group. Statistical significance by two-way ANOVA (mixed-effects analysis) followed by Šidák’s or Tukey’s test. Source data

M-mode echocardiography confirmed the dramatic reduction in LV cavity size beginning at D6—more pronounced for end-diastolic than end-systolic volume—accompanied by transient (D6) increases in septal and posterior wall (PW) thickness that partially recovered at later time points (Extended Data Fig. 3a–d). LV mass remained unchanged (Fig. 2h), whereas relative wall thickness (RWT) increased at D6 and subsequently recovered (Fig. 2i). Heart rate (Extended Data Fig. 3e), fractional shortening (Fig. 2j) and LVEF (Fig. 2k) remained stable, whereas SV and cardiac output were reduced at D6–D13 (Fig. 2l,m). Together, these findings indicate that TC initially preserves systolic function while impairing diastolic performance, followed by the modest compensation of several parameters but the progressive deterioration of cardiac output over time.

Doppler imaging (Fig. 2n) revealed diminished MV flow, with both early (Fig. 2o, MV E) and late (Fig. 2p, MV A) filling velocities decreased at D6–D10, followed by partial recovery. The E/A ratio remained preserved throughout (Extended Data Fig. 3f). Pressure half time was prolonged, and acceleration slowed at D6–D10, returning to baseline by D13 (Extended Data Fig. 3g,h). Early diastolic (e′) and atrial ‘kick’ (a′) velocities exhibited only minor changes (Extended Data Fig. 3i,j); however, the e′/a′ ratio was lowered at D10 (Fig. 2q). Doppler assessment further showed that both the E/e′ ratio, reflecting LV filling pressure, and the isovolumic relaxation time were largely unchanged (Fig. 2r and Extended Data Fig. 3k). In addition, aortic flow velocity and aortic valve pressure gradients were clearly reduced from D6 onward (Fig. 2s,t and Extended Data Fig. 3l–n). Collectively, these findings indicate low-output failure due to restricted ventricular filling, unusually, without evidence of increased myocardial stiffness.

**Extended Data Fig. 4:**
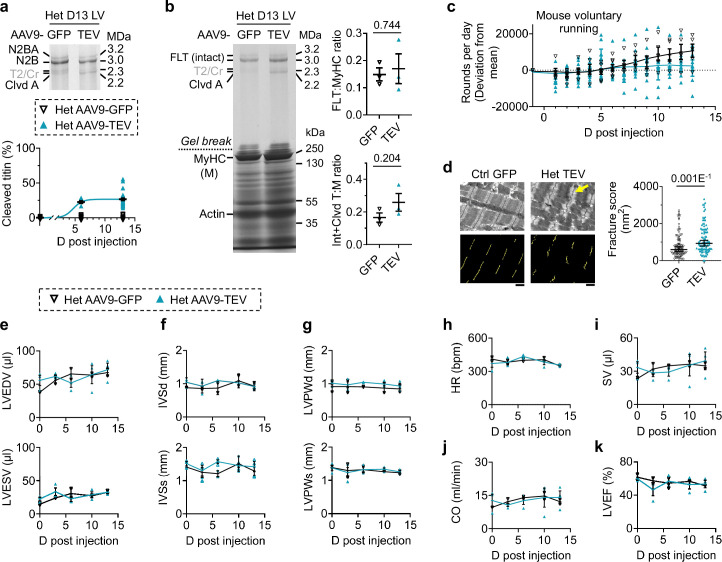
Consequences of in vivo TC in heterozygous (Het) TC mouse hearts. **a**, Coomassie-stained titin gel of left ventricular (LV) tissue at D13 post-AAV9 injection, with time-course quantification of cleaved titin in TEV- versus GFP-injected hearts (TEV D0: N = 7 (n = 7); TEV D6: N = 3 (n = 6); TEV D13: N = 9 (n = 30); GFP D6: N = 9 (n = 13); GFP D13: N = 3 (n = 12)). **b**, Two-phase polyacrylamide gel (1.8% for titin, 7.5% for other cardiac proteins) of D13 LV extracts from AAV9-GFP or AAV9-TEV-injected Het TC mice, with densitometric analysis (*right*) of full-length (*upper*) or total titin (*lower*) to myosin heavy chain (MyHC) ratio (N = 3 per group). **c**, Voluntary running activity showing daily lap count deviation from baseline in Het TC mice following AAV9-GFP: N = 6 (D1, D4), N = 8 (D2-3, D5-6), N = 5 (D7-13) mice/day) or AAV9-TEV: N = 13 (D1, D8-13), N = 14 (D2-4, D7), N = 16 (D5-6) mice/day. **d**, Transmission electron micrographs of D13 GFP- and TEV-injected Het TC hearts (arrow indicates wavy Z-disk; scale bars: 1 µm), with semi-automated fracture scoring of Z-disk disorder (n = 99 (GFP); n = 94 (TEV) Z-disks; median ± 95% CI). **e-k**, Transthoracic echocardiography in Het TC mice (Het TEV: N = 3; Het GFP: N = 2) reporting: **e**, LV end-diastolic and end-systolic volumes (LVEDV, LVESV); **f**, Interventricular septum width in diastole and systole (IVSd, IVSs); **g**, LV posterior wall thickness in diastole and systole (LVPWd, LVPWs); **h**, Heart rate (HR) under anesthesia; **i**, Stroke volume (SV); **j**, Cardiac output (CO); and **k**, LV ejection fraction (LVEF). Data are mean ± SEM except in (**d**); statistical significance by unpaired t-test. Source data

To determine the threshold of TC required to alter key morphological and functional parameters, we also analyzed Het TC mice, in which TEV expression cleaved ~27% of titin at D13 (Extended Data Fig. 4a). Key biochemical and functional measures remained unchanged between TEV- and GFP-injected Het mice, although mild trends toward increased total titin expression (Extended Data Fig. 4b) and reduced voluntary running activity could be observed (Extended Data Fig. 4c). Interestingly, the Z-disk fracture score^35^—a sensitive marker of subcellular disruption—was considerably elevated in TEV-injected Het hearts (Extended Data Fig. 4d), whereas no echocardiographic parameters were affected (Extended Data Fig. 4e–k). Plotting parameter changes against the percentage of TC (in Het and Hom mice) revealed that LV end-diastolic volume and cardiac output were preserved until cleavage exceeded ~30% (Fig. 2u). Conversely, TEV-injected mice succumbed when TC reached ~80%, a threshold achieved by administering a threefold higher dose of AAV9-TEV (3 × 10^12^ viral particles), resulting in death by D6 post injection.

### Loss of restoring forces with cleavage

**Fig. 3:**
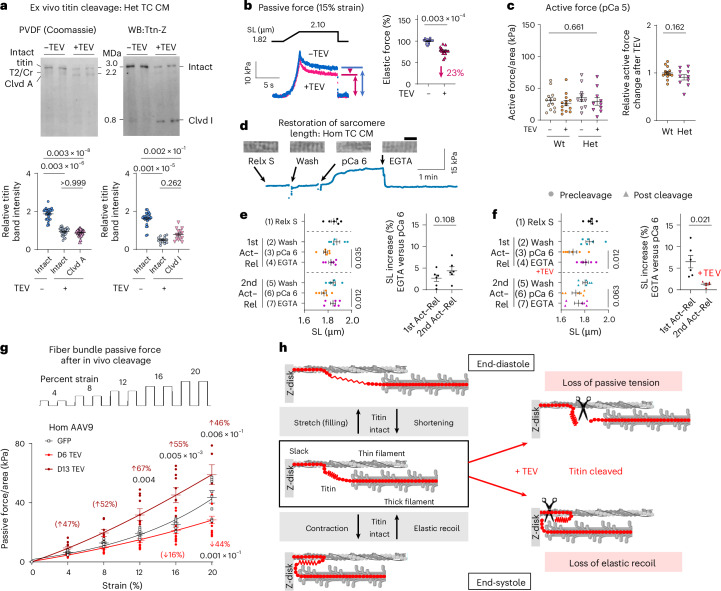
Impaired passive tension and elastic recoil in titin-cleaved cardiomyocytes. **a**, Coomassie-stained PVDF and WB against Ttn-Z in permeabilized, ex vivo TEV-treated, Het TC LV cardiomyocytes (CM) show ~50% TC after 30 min (Coomassie *n* = 28 gel lanes; WB *n* = 15; Kruskal–Wallis and Dunn’s test). **b**, The stretch protocol, sample passive force traces and mean relative passive elastic force before and after ex vivo TEV treatment (*n* = 15 Het cells per group; two-tailed, paired *t*-test). **c**, The mean Ca^2+^-activated (pCa 5) force per cross-sectional area in permeabilized Wt and Het CM pre and post TEV incubation and relative change in active force with TEV (*n* = 15; one-way ANOVA (left) and two-tailed, unpaired *t*-test (right)). **d**, Representative force trace and phase images of sarcomeres during CM activation (pCa 6) and relaxation (EGTA). Scale bar, 5 µm. **e**, In untreated Hom cells, resting SL is fully restored over two successive activation–relaxation (Act–Rel) cycles; the mean SL increase during EGTA relaxation relative to pCa 6 activation (*n* = 6; repeated-measures one-way ANOVA or Tukey (left) and paired *t*-test (right)). **f**, After 20 min TEV, SL fails to recover during EGTA relaxation; the quantification of SL increase versus precleavage (*n* = 6; one-way ANOVA or Tukey (left) and two-tailed, paired *t*-test (right)). **g**, The passive stress–strain curves of fiber bundles from Hom TC LV injected with AAV9–GFP or AAV9-TEV at D6 or D13 (*n* = 8/14/10 bundles; *N* = 2 animals per group); second-order exponential fits; red numbers indicate percent change versus GFP, black numbers *P* values (one-way ANOVA or Tukey) and parentheses nonsignificant trends. **h**, A schematic summarizing how TC impairs cardiac mechanics. Data are mean ± s.e.m. Relx S, relaxing solution; pCa, −log_10_[Ca^2+^]; EGTA, calcium chelator. Schematic in **h** created in BioRender; Linke, W. https://biorender.com/whc6ccb (2026). Source data

Intrigued by the functional phenotype of titin-cleaved hearts, we isolated cardiomyocytes from Het TC hearts (50% cleavable titins) for ex vivo mechanical analysis. Cells were permeabilized and incubated with TEV for 20–30 min, thereby cleaving approximately half of the titin springs in a controlled manner (Fig. 3a)—closely matching the usual in vivo extent of TC between D6 and D13. Single cardiomyocytes were mounted between a force transducer and a micromotor, and stretch-induced passive forces, sarcomere length (SL) and Ca^2+^-dependent active forces were measured. In permeabilized Het TC cardiomyocytes, ex vivo TEV treatment reduced passive force by ~23% at an average ‘physiological’ strain of ~15% (Fig. 3b), whereas maximum Ca^2+^-activated force remained unchanged relative to TEV-treated wild-type (Wt) cardiomyocytes (Fig. 3c).

We next assessed the ability of permeabilized, lightly adherent TC cardiomyocytes to recover SL following contraction. To maximize the observable effect, we used Hom cells, which express 100% cleavable titins, and employed an intermediate calcium concentration (pCa 6) to minimize contraction-induced cellular damage. Cells activated at pCa 6 and then rapidly relaxed with EGTA (Fig. 3d) showed complete SL recovery over two successive cycles when uncleaved (Fig. 3e); by contrast, TC after the first cycle clearly impaired SL restoration during the second cycle (Fig. 3f). These findings indicate that TC reduces intrinsic restoring forces, thereby impairing SL recovery and cardiomyocyte elastic recoil.

To more directly evaluate the mechanical impact of TC in vivo, we isolated cardiac fiber bundles from Hom TC hearts injected with AAV9–GFP or AAV9-TEV and measured passive stress during stepwise stretches to 20% strain (Fig. 3g). As anticipated, passive stress was reduced in D6 TEV-injected samples relative to GFP controls, particularly at higher strains. However, by D13, TEV-injected fibers exhibited a marked increase in passive tension at intermediate-to-high strain levels, an effect unlikely attributable to further TC and more plausibly secondary to ECM remodeling. We conclude that, before compensatory remodeling, TC primarily reduces passive tension and, critically, impairs elastic recoil (Fig. 3h), the latter probably accounting, at least in part, for the profound reduction in ventricular filling observed in vivo.

### Cell–matrix crosstalk and ECM remodeling

**Fig. 4:**
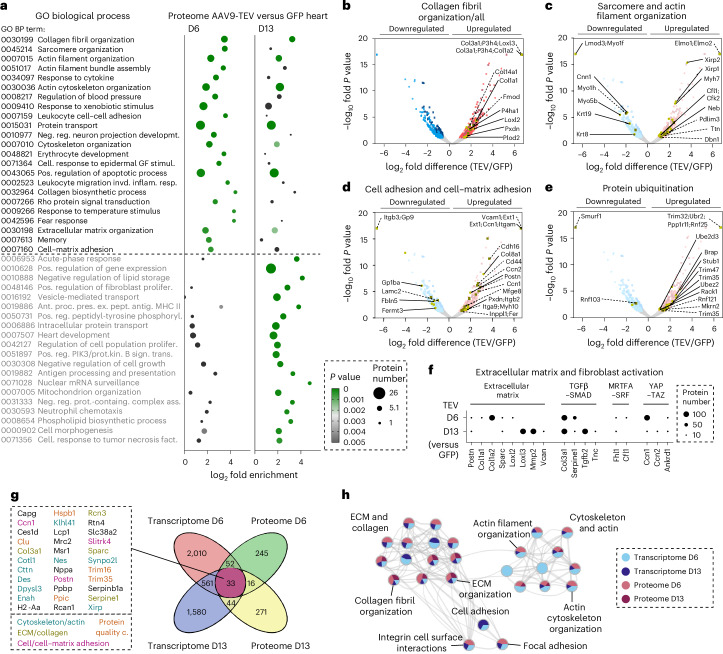
Molecular profiling of titin-cleaved hearts. **a**, The GO biological process (BP) analysis of significantly up- or downregulated proteins in TEV versus GFP samples. The color indicates *P* value (threshold 0.005; modified Fisher’s exact test (EASE score)); the circle size reflects the number of proteins. Highly significant GO terms (*P* ≤ 0.001; modified Fisher’s exact test (EASE score)) are ordered by increasing *P* value at D6 and separated (dashed line) from enriched GO terms with *P* > 0.001 at D6 (dark gray circles). **b**–**e**, The volcano plots for proteins in TEV:GFP samples at D6 (circles) and D13 (squares), for select GO terms: collagen fibril organization (GO:0030199) (**b**), sarcomere and actin filament organization (GO:0045214; GO:0007015) (**c**), cell adhesion and cell–matrix adhesion (GO:0007160; GO:0007155) (**d**) and protein ubiquitination (GO:0016567) (**e**). All proteins with fold change >1.5 (*P* < 0.05; ‘background-based’ paired two-sided *t*-test) are color-coded—blue for downregulation and red for upregulation (shown as faint color in **c**–**e**)—whereas proteins designated by the respective GO terms are highlighted in yellow; select proteins are identified via solid (D6) and dashed lines (D13). **f**, The protein changes within common ECM and fibroblast activation pathways. **g**, A Venn diagram illustrating the overlap of significantly altered proteins and genes from the proteomic and transcriptomic datasets. The 33 common molecules are listed, with key molecules and pathways highlighted. **h**, A network analysis combining D6 and D13 proteomic and transcriptomic data. Each circle node is a pie chart representing a GO term; the pie sections are proportional to the normalized gene count from each ‘omic’ dataset. Nodes with a similarity score >0.3 are connected. The network visualization was performed using Cytoscape. D6 *N* = 6 (Hom TEV; Het GFP); *N* = 3 (Hom GFP); D13 *N* = 6 (Hom TEV; Hom GFP). Source data

**Extended Data Fig. 5:**
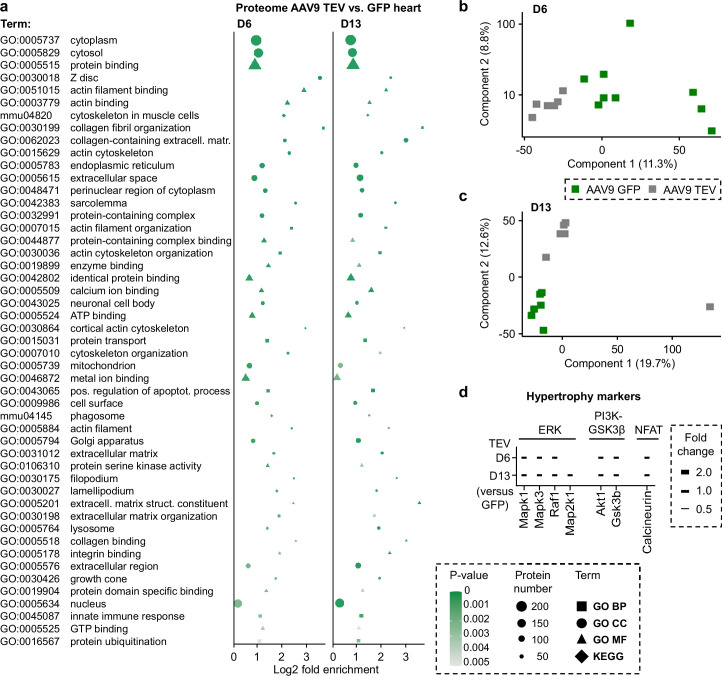
Additional proteomics analyses of in vivo titin-cleaved hearts. **a**, Gene ontology (GO) and KEGG enrichment of proteins significantly altered in TEV- versus GFP-injected left ventricles, analyzed with DAVID and plotted in R (ggplot2). Point color denotes *p* ≤ 0.005; size reflects the number of proteins. GO biological processes (BP) are shown as squares, cellular components (CC) as circles, molecular function (MF) as triangles, and KEGG pathways as diamonds. Displayed terms, sorted by increasing *p*-value at D6, were significant at both D6 and D13. **b-c**, Principal component analysis of left-ventricular proteomes at D6 (Hom TEV N = 6, Hom GFP N = 3, Het GFP N = 6) and D13 (Hom TEV N = 6, Hom GFP N = 6) post-AAV9 injection. **d**, In the proteomic analysis at D6 and D13 post-AAV9 injection, cardiac hypertrophy markers were detected but showed no significant differences between TEV- and GFP-injected hearts (all *p* > 0.05). Source data

**Extended Data Fig. 6:**
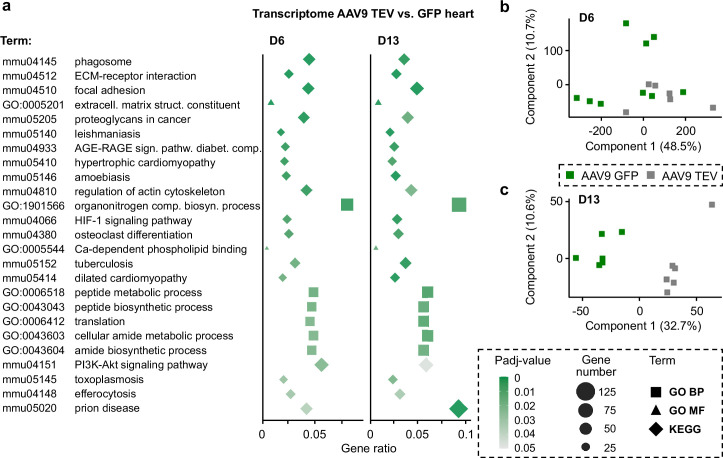
Additional transcriptomics of in vivo titin-cleaved mouse hearts. **a**, Gene Ontology (GO) and KEGG pathway enrichment analyses of transcripts significantly altered in TEV- versus GFP-injected left ventricles were performed using DAVID and plotted in R (ggplot2). Point color denotes adjusted *p* ≤ 0.05, and point size reflects the number of genes. GO biological processes (BP) are shown as squares, molecular functions (MF) as triangles, and KEGG pathways as diamonds. Displayed terms, ordered by increasing *p*-value at D6, were significant at both D6 and D13. **b-c**, Principal component analysis of transcriptomes at D6 (Hom TEV N = 6, Het GFP N = 6, Hom GFP N = 3) and D13 (Hom TEV N = 6, Hom GFP N = 6) post-AAV9 injection. Source data

To elucidate molecular changes in the myocardium following TC, we performed proteomic and transcriptomic analyses on LV tissues from TEV- versus GFP-injected TC mice at D6 and D13. Gene Ontology (GO) and Kyoto Encyclopedia of Genes and Genomes (KEGG) enrichment revealed consistently altered proteomic terms at both time points but fewer common transcriptomic terms (Fig. 4a and Extended Data Figs. 5 and 6), indicating more stable shifts in protein-level processes versus transient gene expression changes. Principal component analysis clearly segregated TEV- and GFP-injected proteomes at both days (Extended Data Fig. 5b,c), whereas transcriptomic separation emerged only by D13 (Extended Data Fig. 6b,c).

At D6, key upregulated proteomic GO terms included ‘collagen fibril organization’, ‘extracellular matrix organization’, ‘sarcomere organization’, ‘actin cytoskeletal organization’, and ‘cell–matrix adhesion’ (Fig. 4a). Most of these remained altered by D13 (Fig. 4a, top), alongside additional terms suggestive of compensatory responses (Fig. 4a, bottom). Transcript-level enrichment likewise featured ‘ECM–receptor interaction’, ‘focal adhesion’ and ‘regulation of the actin cytoskeleton’ at both days (Extended Data Fig. 6a). Volcano plots highlighted upregulation of collagen-organizing proteins (Col1a1, Col3a1, Loxl2 and Loxl3) and selected sarcomeric and cytoskeletal proteins (Myh7, Xirp1/2 and titin), with downregulation of others (Lmod3, Myo1f and Myo5b) and variable adhesion molecules (increased Postn, Col8a1, Vcam1 and Itgb2; decreased Lamc2, Fbln5 and Itgb3) (Fig. 4b–d). Several other proteins fell under the ‘protein quality control’ umbrella, as evidenced by the proteomic enrichment of ubiquitination pathways and upregulation of E3 ubiquitin ligases at both time points (Fig. 4e). Inflammatory pathway alterations intensified by D13 (Fig. 4a), although innate immune and mitochondrial remodeling processes were already evident at D6 (Extended Data Fig. 5a). Within ECM and fibroblast activation pathways, canonical effectors (Tgfb–Smad, MRTFA–SRF and Yap–Taz) were not detected; nevertheless, associated proteins appeared with divergent activation patterns at D6 and D13 (Fig. 4f). Remarkably, no hypertrophy markers were enriched at either time point (Extended Data Fig. 5d).

The Venn analysis identified a core set of 33 genes and proteins commonly dysregulated at D6 and D13, predominantly linked to cytoskeletal organization, ECM and collagen remodeling and cell-adhesion functions, as well as protein quality control (Fig. 4g). Network analysis further corroborated these links (Fig. 4h). Among the set of upregulated molecules was the heart-failure marker A-type natriuretic peptide (encoded by NPPA). Moreover, the GO term ‘positive activation of apoptotic process’ was enriched at D6 and D13, suggesting the concurrent activation of rescue-and-removal mechanisms (Fig. 4a).

**Extended Data Fig. 7:**
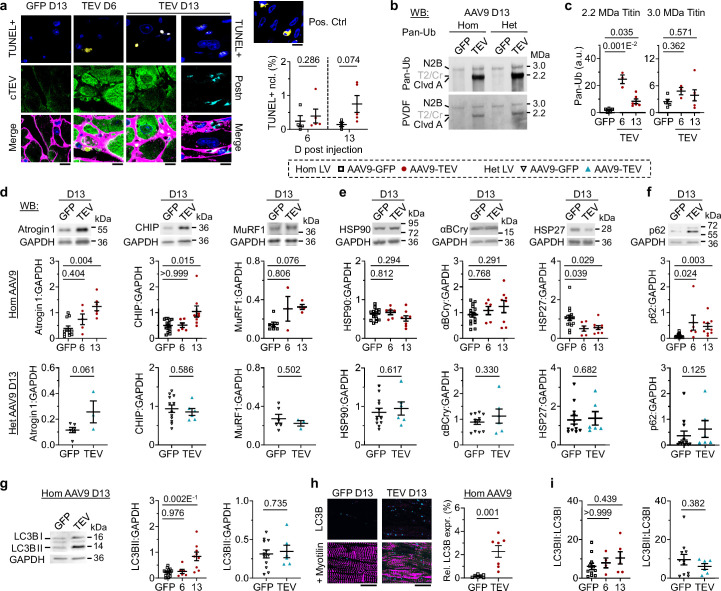
Apoptosis and protein quality control in titin-cleaved hearts. **a**, TUNEL/DAPI staining (yellow/blue) of left ventricular (LV) sections from Hom TC mice at D6 and D13 after AAV9-GFP or AAV9-TEV injection; mouse pancreatic tumor cells as positive control (Pos. Ctrl). Co-staining using anti-cTEV (cleaved titin; green, Cy3) or anti-periostin (Postn, myofibroblasts; cyan, AlexaFluor 488); merge includes wheat germ agglutinin staining (magenta, AlexaFluor 647). Scale bars, 20 µm. Graph shows quantification of TUNEL+ nuclei (ncl.) as a percentage of DAPI-positive cells (GFP N = 4; TEV N = 5 mice/group). **b**, Pan-ubiquitin (Pan-Ub) Western blot (WB) and Coomassie-stained PVDF membrane of titin in Hom and Het TC hearts at D13. **c**, Densitometry quantification of Pan-Ub titin normalized to Coomassie signal: cleaved fragment (~2.2 MDa) and full-length N2B (~3.0 MDa) in Hom hearts (GFP N = 5; TEV N = 6). **d-f**, WBs and protein quantification of heart tissue (top, Hom; bottom, Het TC) comparing AAV9-TEV at D6/D13 versus AAV9-GFP at D13 for **d**, E3 ubiquitin ligases Atrogin-1, CHIP/Stub1, MuRF1; **e**, Chaperones HSP90, αB-Crystallin, HSP27; and **f**, Autophagy adaptor p62. **g**, WBs for LC3B (left, GAPDH as loading control) and summary ratios of LC3BII:GAPDH, in Hom (middle) and Het (right) TC LV versus GFP. **h**, Immunofluorescence images of Hom LV sections at D13 (scale bars, 10 µm), stained for LC3B puncta (Cy3) and myotilin (AlexaFluor 488), and LC3B puncta frequency per unit area (right, n = 7 images/group). **i**, Summary ratios of LC3BII:LC3BI protein WBs in Hom (left) and Het (right) TC LV versus GFP. For WBs: Hom GFP N = 8, TEV N = 5; Het GFP N = 6, TEV N = 3. Data are mean ± SEM. Statistical significance by unpaired t-test or one-way ANOVA followed by appropriate multiple comparisons test. Source data

To independently validate these rescue pathways, we assessed apoptosis via the TUNEL assay, which revealed a trend toward increased apoptotic activity in titin-cleaved hearts (Extended Data Fig. 7a). However, costaining for cleaved titin using anti-cTEV together with the activated fibroblast marker periostin demonstrated that TUNEL-positive cells were fibroblasts, not cardiomyocytes (Extended Data Fig. 7a). Anti-pan-ubiquitin WBs, leveraging titin’s large size to minimize signal interference (Extended Data Fig. 7b), indicated that cleaved (but not intact) titin was more heavily ubiquitinated in both Hom and Het LV tissues, particularly at D6 (Extended Data Fig. 7c), suggesting rapid targeting for proteasomal degradation. Muscle E3 ligases (Atrogin-1, CHIP [Stub1] and MuRF1) were elevated in titin-cleaved Hom LV but not in Het (Extended Data Fig. 7d). Moreover, known I-band titin-binding chaperones^36,37^ were either unchanged (HSP90, αB-crystallin) or downregulated (HSP27), at least in Hom LV (Extended Data Fig. 7e), whereas the autophagy adapter p62 was upregulated in Hom but not Het (Extended Data Fig. 7f). Normalized LC3BII content increased in D13 Hom hearts but not in Het (Extended Data Fig. 7g). In addition, LC3B puncta were observed by IF in D13 Hom TC hearts, demonstrating the increased presence of autophagosomes (Extended Data Fig. 7h). However, the LC3BII:LC3BI ratio remained unchanged, indicating limited autophagic flux (Extended Data Fig. 7i). Collectively, these findings indicate that TC rapidly activates intracellular protein quality control mechanisms, whereas cardiomyocyte apoptosis remains minimal.

### Fibroblast activation by TC

**Fig. 5:**
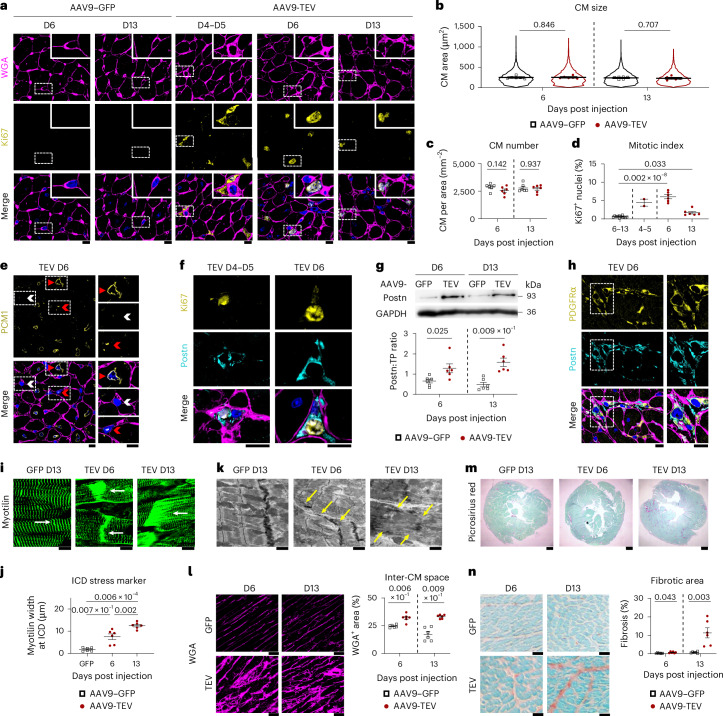
Myocardial remodeling in titin-cleaved hearts. **a**, The IF images of Hom TEV LV sections at D4–D5, D6 and D13 versus GFP D6 and D13 post AAV9 injection: WGA (magenta; AlexaFluor 555) labels cell borders; Ki67 (AlexaFluor 488) marks cycling cells; DAPI (blue; added in merge) stains nuclei. Insets: ROIs. **b**,**c**, The semi-automated quantification of CM size (**b**) and number (**c**), in 3,324–3,662 cells per group (*N* = 6); the violin plots display individual distributions. **d**, The Ki67^+^ nuclei as percentage of total nuclei (3,928 total nuclei for TEV D4–D5, *N* = 2; 12,155–17,063 total nuclei for GFP and TEV D6/D13, *N* = 6). Data at 4–5 days post injection were not included in the statistical testing (dashed lines). **e**, The IF for PCM1 (AlexaFluor 488) and merge with WGA (magenta) and DAPI (blue) at D6 post TEV injection; the red arrows indicate quiescent CM, red arrowheads cycling interstitial cells and white arrowheads resting interstitial cells. **f**, The Ki67^+^ activated fibroblasts (Cy3), costained for periostin (Postn; AlexaFluor 488) and merge with DAPI and WGA at D4–D5 and D6 post AAV9-TEV. **g**, The Postn expression by WB relative to the total protein (TP), with GAPDH as loading control. **h**, The PDGFRα^+^ activated fibroblasts (Cy3), costained for Postn (AlexaFluor 488) and merge with DAPI (blue) and WGA (magenta), at D6 post AAV9-TEV. **i**, The IF of ICDs (arrows) in longitudinal sections stained for myotilin (AlexaFluor 488) from AAV9–GFP and AAV9-TEV LV at D6 and D13. **j**, The quantification of myotilin signal width at ICDs (*N* = 6 per group). **k**, The TEM of ICDs; the yellow arrows denote distorted junctions. Representative images from 50 independent images. **l**, The WGA staining of Hom TC LV at D6 and D13 (GFP versus TEV) and the quantification of the WGA^+^ intercellular area as a fraction of total area at D6 and D13 (*N* = 6). **m**, The Picrosirius red staining at D6 and D13 comparing TEV and GFP LV. **n**, The detail of Picrosirius red-stained LV and quantification of interstitial fibrosis (Picrosirius red^+^ area/total area; *N* = 6). Data are mean ± s.e.m.; statistical significance by unpaired two-tailed *t*-test or one-way ANOVA with Tukey’s test. Scale bars, 10 µm (**a**,**e**,**f**,**h**,**i**,**l**,**n**), 500 µm (**m**), 1 µm (**k**). Representative images selected from >20 independent images. Source data

Given that our omics data indicated altered cell–matrix interactions and ECM reorganization already at D6, we further explored these aspects by microscopic tissue analysis. Wheat germ agglutinin (WGA) staining of tissue cross-sections (Fig. 5a) allowed the quantification of cardiomyocyte size and number, revealing no evidence of hypertrophy or hyperplasia (Fig. 5b,c). However, Ki67 staining indicated increased mitotic activity, with Ki67-positive nuclei elevated as early as D4 and D5 post TEV injection (when TC was still only 12–20% (Fig. 1f)), peaking at 6% on D6 and declining to 2% by D13, whereas GFP controls remained below 1% (Fig. 5a,d). The costaining of pericentriolar material 1 (PCM1)^38^, DAPI and WGA distinguished resting cardiomyocytes (perinuclear PCM1) from resting (PCM1-negative) and cycling (intranuclear PCM1) interstitial cells (Fig. 5e). Colabeling of Ki67 with periostin demonstrated that the proliferating cells were myofibroblasts confined to interstitial niches (Fig. 5f). On WBs, periostin was increased in TEV D6 and D13 LV tissues (Fig. 5g). Moreover, periostin-positive cells at TEV D6 were also positive for platelet-derived growth factor receptor A (PDGFRα), confirming that the Ki67-positive cells represent proliferating activated cardiac fibroblasts rather than cardiomyocytes (Fig. 5h). Together, these findings strongly suggest immediate fibroblast activation in response to titin stiffness loss that precedes any overt functional impairment.

### Stress imbalance and onset of fibrosis

We hypothesized that the loss of titin stiffness induces internal stress and disrupts intra- and intercellular connections, thereby activating fibroblasts and driving fibrosis, and we set out to demonstrate this principle. Anti-cTEV staining revealed that titin-cleaved sarcomeres detached from ICDs (Fig. 1h, arrows), whereas myotilin staining showed ICD-associated ‘streaming’ (Figs. 1h and 5i). The quantification in longitudinal LV sections demonstrated an expanded myotilin-positive region at TEV D6, which further widened by D13 (Fig. 5j), indicating increased mechanical deformation. Electron microscopy confirmed progressive ICD distortion between D6 and D13 (Fig. 5k). Strikingly, WGA staining revealed a greatly enlarged intercardiomyocyte space at D6 and D13 (Fig. 5l), primarily reflecting loss of cell-cell contact rather than scar tissue formation. These expanded gaps are likely to contribute to the LV wall thickening observed in titin-cleaved hearts.

As expected from the fibroblast activation timeline, Picrosirius red staining showed a modest increase in interstitial fibrosis at D6, progressing to a pronounced rise by D13 (Fig. 5m,n), in line with our multiomics data and the elevated passive stiffness of isolated fiber bundles at D13. Thus, TC leads to mechanical disconnection and imbalance, rapidly activating fibroblasts and subsequently promoting interstitial fibrosis as a maladaptive remodeling response.

### Deregulation of cell–cell connectivity

**Fig. 6:**
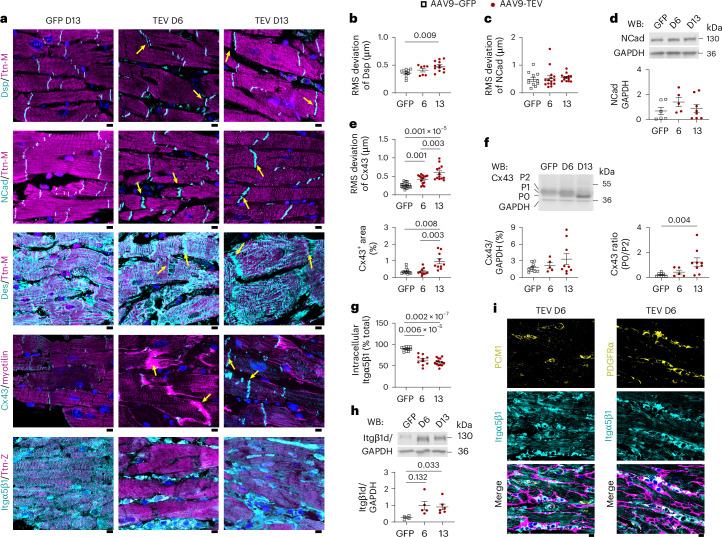
ICD, costamere and ECM proteins in titin-cleaved heart tissue. **a**, The IF staining of LV Hom TC tissue for desmoplakin (Dsp), N-cadherin (NCad) and desmin (Des) (all AlexaFluor 488), each costained with Ttn-M (Cy3), as well as Cx43 (AlexaFluor 488) costained with myotilin (Cy3) and integrin α5β1 (Itgα5β1, AlexaFluor 488) costained with Ttn-Z (Cy3). Yellow arrows highlight ICD regions. Representative images (selected from >20 independent images) are shown for GFP- and TEV-injected samples at D6 and D13. **b**, The RMS deviation of Dsp in IF images (GFP *n* = 10, TEV D6 *n* = 7 and TEV D13 *n* = 12). **c**, The RMS deviation of NCad (GFP *n* = 11, TEV D6 *n* = 15 and TEV D13 *n* = 13). **d**, The WB for NCad with GAPDH as loading control (top) and NCad expression normalized to GAPDH (bottom) (GFP *N* = 6 animals, TEV D6 *N* = 5 and TEV D13 *N* = 8). **e**, The RMS deviation of Cx43 on IF images (top) (GFP *n* = 19, TEV D6 *n* = 15 and TEV D13 *n* = 13) and the Cx43^+^ area as percentage of total area (bottom) (*n* = 10 images). **f**, The WB for Cx43 (top), Cx43 normalized to GAPDH (bottom left) and Cx43 quantified as the ratio of nonphosphorylated (P0) to phosphorylated forms (bottom right) (GFP *N* = 9, TEV D6 *N* = 5 and TEV D13 *N* = 9). **g**, The intracellular integrin (Itgα5β1) as percentage of total integrin in IF images (GFP *n* = 12, TEV D6 *n* = 10 and TEV D13 *n* = 14). **h**, The WB for Itgβ1d (top) and Itgβ1d expression normalized to GAPDH (bottom) (*N* = 4 for GFP, *N* = 5 for TEV D6 and *N* = 6 for TEV D13). **i**, The IF of AAV9-TEV-injected mouse LV (D6) stained for PCM1 (Cy3) (left) or PDGFRα (Cy3) (right), Itgα5β1 (AlexaFluor 488) and merged with WGA (magenta; AlexaFluor 647) and DAPI (blue). Representative images selected from ten independent images. Data are mean ± s.e.m. Statistical significance by one-way ANOVA with Tukey’s post hoc test. Scale bars, 10 µm (**a**,**i**). Source data

Next, we investigated changes in cardiac mechanical connectivity following TC, focusing on key proteins mediating cell–cell and cell–matrix interactions. The IF of LV tissues (Fig. 6a) showed that major ICD components, including desmoplakin (desmosomes) and N-cadherin (adherens junctions), remained largely aligned (Fig. 6b,c), and N-cadherin expression was unaltered (Fig. 6d). However, desmin filaments appeared more irregular in titin-cleaved cardiomyocytes than in GFP controls and were frequently detached from the ICD, suggesting increased internal stress (Fig. 6a). Notably, the gap junction protein connexin 43 (Cx43) showed progressive misalignment from D6 to D13 post TEV (Fig. 6a,e), increased expression at D13 (Fig. 6e,f) and partial internalization into cardiomyocytes that became more pronounced by D13 (Fig. 6a). WBs further demonstrated a Cx43 mobility shift from P2 (phosphorylated) to P0 (unphosphorylated), indicating loss of function^39^ (Fig. 6f). Thus, titin-cleaved cardiomyocytes lose functional gap junctions as part of impaired cell–cell connectivity.

Finally, we examined integrin α5β1 complexes, which connect sarcomeric Z-disks to costameres via actin filaments and associated cytoskeletal networks and are typically upregulated in stressed cardiomyocytes^40^. By IF, α5β1 integrins showed a diffuse but regularly striated sarcolemmal pattern with occasional perinuclear staining in GFP control tissue (Fig. 6a). Following TC, these complexes became disorganized and accumulated dramatically, predominantly localizing to perinuclear areas within the expanded extracardiomyocyte space, whereas intracellular integrin was strongly reduced from D6 to D13 (Fig. 6g). WBs also showed increased integrin β1d expression during this period (Fig. 6h). The costaining of integrin α5β1, WGA, DAPI and PCM1 or PDGFRα (Fig. 6i) confirmed that TC redistributed integrin α5β1 to perinuclear regions of fibroblasts, indicating severely disrupted cell–matrix connections. Taken together, the loss of titin stiffness and the resulting force imbalance compromise sarcomere–ICD connectivity, promote lateral cardiomyocyte detachment and disrupt cardiomyocyte–matrix links, thereby destabilizing the mechanical homeostasis sensed by fibroblasts and triggering matrix remodeling.

## Discussion

**Extended Data Fig. 8:**
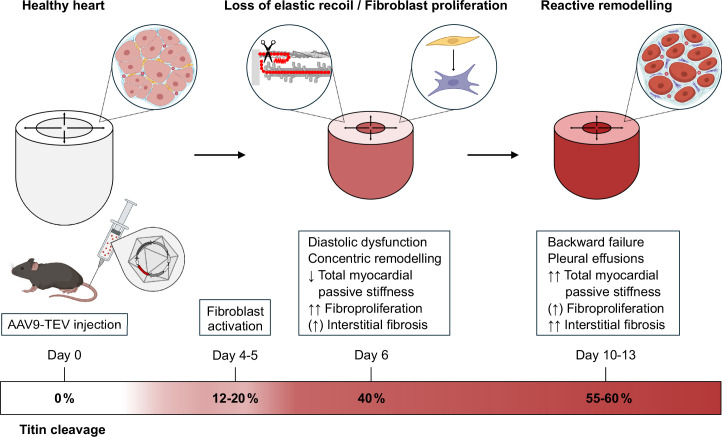
Overview of key events in the heart triggered by selective proteolytic cleavage of elastic titin. Figure created in BioRender; Linke, W. https://biorender.com/6p4wcjc (2026).

Our study provides insights into the dual functions of titin-based sarcomere stiffness in cardiac mechanics: generating elastic recoil and orchestrating cell–matrix crosstalk. By selectively cleaving titin springs in vivo near the native T2 cleavage site—thereby mimicking proteolytic TC in ischemic or chemotherapy-induced myocardial injury^17–20^—we show that the acute loss of passive stiffness profoundly impairs restoring forces, trapping cardiomyocytes in a shortened state and markedly reducing LV cavity size despite initially preserved systolic function. This restrictive filling phenotype, accompanied by modest wall thickening, arises not from hypertrophy, atrophy or hyperplasia but from impaired SL restoration, disrupted cell–cell and cell–matrix interactions and expanded intercardiomyocyte space, which are collectively consistent with the collapse of mechanical homeostasis. An overview of the sequence of myocardial remodeling following TC is provided in Extended Data Fig. 8.

Although titin has long been recognized as a molecular spring that limits cardiomyocyte extensibility and myocardial distensibility^5,6^, its in vivo contribution to restoring force generation, predicted from isolated cell studies^33,34^, had remained unresolved. Here, we demonstrate that these forces, which drive cardiomyocyte re-lengthening^41,42^ and are frequently compromised in disease^43,44^, are functionally indispensable. This property probably reflects the function of the stiff N2B titin isoform, whose short elastic segment enables bidirectional spring behavior, restoring SL from both stretched and shortened states^33,34^ (Fig. 3d–f,h). N2B predominates in most mammalian species, including human myocardium (~60–70% of full-length titin)^12^, compared with ~80–90% in mice (Fig. 1g). Isoform switching from N2B to the more compliant N2BA occurs in ischemic HF, dilated cardiomyopathy and HF with preserved ejection fraction^12–15^, probably compromising elastic recoil and thereby contributing to disease progression.

Single-cardiomyocyte assays revealed that TC reduces resting SL following rapid relaxation after contraction, consistent with the reduced LV cavity size observed in vivo. The accompanying increase in LV wall thickness may partly reflect increased sarcomere diameter at shorter lengths, resulting from the expansion of myofilament lattice spacing. Titin contributes to lattice spacing control, whereas TC reduces transverse sarcomere stiffness^45^ and further expands lattice spacing^46^, thereby promoting wall thickening. Additional contributions may arise from the widened intercardiomyocyte space following in vivo TC (Fig. 5l). The loss of elastic recoil presumably represents the initiating event in geometric remodeling, followed by secondary effects including increased internal stress, disruption of mechanical homeostasis and altered hemodynamics (low flow across the aortic valve and MV). Importantly, these changes can be triggered by proteolytic cleavage at a single I-band titin site. The precise cleavage location is probably less critical than disruption of elastic I-band continuity. MMP2 cleaves titin in the mid-I band near our TEV site, generating similar T2 fragments (~2.2–2.4 MDa)^17^ and is therefore predicted to produce comparable structural and functional consequences in stressed human hearts.

The acute restrictive phenotype in our model contrasts with the dilated cardiomyopathy seen in patients and animal models with RBM20 mutations that reduce N2B while increasing N2BA titin^14,47^. Whereas chronic titin destiffening promotes chamber dilation, our data suggest an initial restrictive phase that progresses to decompensated failure, characterized by systolic decline and pulmonary congestion by D13. These findings imply that acute titin destiffening primarily impairs filling through the loss of elastic recoil and disruption of intra- and intercellular connectivity, whereas chronic remodeling may ultimately manifest as ventricular dilation.

Although the TC mouse model does not replicate *TTN* truncation disorders, which are typically caused by Het truncating variants^10,21,48^, it provides insight into potential consequences of hypothetical biallelic I-band truncations. Such variants would be incompatible with life because titin could no longer maintain sarcomere integrity and cardiac mechanical function, resulting in intrauterine lethality. Titin defects are implicated in spontaneous abortion, although their prevalence is unclear^49^. Prenatal *TTN*-associated phenotypes extend beyond cardiac abnormalities to skeletal muscle, joint and bone defects^49–51^.

TC also rapidly triggers fibroblast activation and ECM remodeling. Locally altered shear stress and ICD disorganization probably function as profibrotic cues: stress-responsive integrin α5β1 complexes anchored by the β1D isoform in adult cardiomyocytes^52^ become disorganized and accumulate around fibroblast nuclei. This redistribution may disrupt coordinated force sensing and mechanoresponsive signaling, thereby promoting myofibroblast differentiation^23,25^. Gap junction organization is also affected: Cx43 becomes misaligned and partially internalized and shifts toward nonphosphorylated isoforms, impairing channel opening and electrical coupling and promoting arrhythmogenesis^40,53^. Beyond electrical uncoupling, Cx43 dysregulation probably exacerbates mechanical dysfunction through effects on cytoskeletal tension and nuclear mechanotransduction. Although the precise mechanisms linking TC to fibrosis remain incompletely defined, reduced titin stiffness and force imbalance clearly destabilize integrin- and Cx43-mediated signaling, possibly causing local matrix stiffening—initially without overt fibrotic deposition—which is sensed by fibroblasts, as recently reported in a model of sarcomeric hypocontractility^30^.

Multiomics analyses further support these findings, revealing cleavage-induced early activation of cell–matrix adhesion and ECM remodeling pathways, cytoskeletal reorientation and absence of hypertrophic signaling. As I-band titin functions as a scaffold for hypertrophic signaling complexes^5,6^, the cleavage of elastic titin is expected to disrupt these pathways. Consistently, proteomics showed minimal changes in extracellular signal-regulated kinases (ERK2 (Mapk1), ERK1 (Mapk3), Raf1 and MEK1 (Map2k1)), which associate with titin’s N2B region via four-and-a-half LIM-domain proteins; FHL1 itself remained largely unchanged. Early compensatory responses included ubiquitin-mediated protein degradation and selective autophagy, whereas the expression of I-band titin-associated heat shock proteins^36,37^ remained relatively stable, probably because their binding sites are no longer accessible when the cleaved spring recoils toward the Z-disk^35^. Apoptosis remained near baseline and primarily affected fibroblasts. Despite substantial TC on D6 (35–40%), sarcomere loss reached only 8.9%, indicating transient buffering of mechanical failure. However, in the absence of titin stretch signaling, these compensatory mechanisms ultimately fail, leading to progressive fibrosis and rebound in passive tension by D13. This ECM-driven stiffness increase can transiently stabilize myocardial geometry, function and elastic recoil, as reflected by partial recovery of echocardiographic parameters on D10–D13, but cannot prevent rapid decompensation. Thus, a narrow temporal window exists during which the primary consequences of TC can be observed before secondary remodeling dominates.

Our data also suggest a threshold beyond which TC triggers pathological remodeling. Fibroblast proliferation increased at only 12–20% TC (D4 and D5), preceding measurable functional decline. This range closely approximates T2 titin proportions in human hearts (12–14%)^21^, suggesting tightly regulated physiological titin turnover. Excess proteolysis, for example, during oxidative stress, may shift this balance toward fibroblast activation and fibrosis. In healthy myocardium, limited TC probably supports protein turnover without triggering pathological remodeling. In the TC model, cardiac function and geometry remained largely preserved until cleavage exceeded ~30%, delineating a potential therapeutic window. These findings may guide targeted interventions for acquired cardiomyopathies^17,19^, atrial fibrillation^16^ and RBM20-associated inherited cardiomyopathies^14,47^ and support moderate titin destiffening strategies in HF with preserved ejection fraction, such as the controlled upregulation of N2BA isoforms via RBM20 modulation^54,55^.

Proteolytic TC functionally uncouples sarcomeric springs from both the ECM and ICDs, abolishing elastic recoil and precipitating diastolic dysfunction. The disruption of β1D/α5β1 integrin adhesion and Cx43 phosphorylation emerges as a central link between titin mechanical failure, fibroblast activation and fibrotic remodeling. A deeper understanding of titin’s pivotal role in cardiac mechanical homeostasis may open new avenues to prevent maladaptive remodeling and HF progression.

## Methods

### Study design

To investigate the in vivo effects of selective TC on cardiac structure and function, we used adult C57BL/6JRj mice (8–44 weeks, both sexes) engineered with a TEV protease recognition site in the titin spring region^32^. TC was triggered specifically in beating myocardium by intravenous injection of AAV9-TEV under the Tnnt2 promoter; controls received equivalent AAV9–GFP doses. Within each litter, Wt, Het and Hom TC mice were randomized concurrently to TEV or GFP vectors to eliminate litter effects. Longitudinal cardiac structure and function were assessed by cMRI and TTE. Group sizes ranged from 5 to 13 mice per genotype; postmortem analyses included at least two hearts per group with a minimum of six technical replicates per sample. Ex vivo studies included IF, electron microscopy, immunohistochemistry, SDS–polyacrylamide gel electrophoresis (SDS–PAGE) and immunoblotting for TC and protein changes, and force measurements on permeabilized single cardiomyocytes and fiber bundles. LV tissue underwent label-free quantitative proteomics and transcriptomics for downstream molecular profiling. All in vitro experiments lacked formal power calculations; sample sizes (*N* = 5–7 animals) were based on prior experience for detecting genotype and phenotype differences in cardiac muscle properties. No data were excluded. Investigators were unblinded owing to the immediate phenotypic and ultrastructural hallmarks of TC. ChatGPT (OpenAI, v4.0) was used solely to assist with the language editing of the paper. All content was originally written by the authors, who reviewed and take full responsibility for the final text.

### Animal model and muscle preparation

All procedures were approved by North Rhine-Westphalia (Germany) Animal Care and Use Committees (LANUV NRW, 81-02.04.2019.A472). Mice were singly housed in ventilated cages at approximately 22 °C with 12-h light–dark cycles and food and water ad libitum. The TC mouse model^32^ contains a TEV protease recognition site and a HaloTag cassette near titin’s I-band/A-band junction between Ig domains I86 (amino acids 14072–14157) and I87 (residues 14161–14246; UniProt A2ASS6-1). Animals are phenotypically normal unless activated^32^. The genotyping was by PCR (primers: 5′ cgtggtggcttatcttctagc 3′, 5′ ctgttggttcatgcatctcc 3′)^9^. TEV protease was delivered specifically via AAV9 under the cardiac-specific human Tnnt2 promoter. The optimized TEV open-reading frame (pMHT238Δ) was cloned into a self-complementary AAV (scAAV) plasmid; the vector production involved the cotransfection of scAAV and helper plasmid pDP9rs into HEK293T (AAV-293) cells, obtained from Stratagene/Agilent (https://www.integratedsci.com.au/product/aav-293-cells.html) and regularly tested for mycoplasma contamination, with purification and titration as described^56^. AAV9–GFP vector served as control.

Mice were anesthetized (1.5–2.5% isoflurane/O_2_) and injected intravenously with 1 × 10^12^ viral particles (once 3 × 10^12^). From 2 days before until D13 after injection, mice had access to running wheels to induce cardiac stress. TTE was performed on D0 and then twice weekly, with cMRI on D6 and D13 post injection. TTE cohorts (aged 9–44 weeks) included *N* = 15 Hom and Het TEV or GFP animals; cMRI cohorts (8–18 weeks) included *N* = 12 Hom TEV or GFP and *N* = 6 Het TEV or GFP animals. Mice were euthanized by cervical dislocation. Hearts were washed in phosphate-buffered saline (PBS) and snap-frozen for biochemistry or retrogradely perfused for microscopic analysis.

### TTE

Mice were anesthetized (1.5–2.5% isoflurane/O_2_), chest-shaved and imaged on a Vevo 2100 (MS550D, 18–55 MHz; VisualSonics) following established protocols^57^. The heart rate was continuously monitored. The LV structure and function were assessed via parasternal SAX M-mode imaging. LV posterior wall (PW) and IVS were measured directly in systole (s) and diastole (d). RWT was calculated as twice the LV PW thickness at end-diastole (LVPWd) divided by the LV end-diastolic internal diameter (LVIDd) (RWT = 2 × LVPWd / LVIDd). LV end-systolic and end-diastolic volumes were derived from LV Trace. From these, LVEF, fractional shortening, SV and cardiac output were calculated as key performance indicators. The pulsed-wave Doppler imaging of the MV measured E_max_ and A_max_, from which the E/A ratio was determined. Pressure half time and acceleration were also assessed. Tissue Doppler imaging of the medial and lateral mitral annulus was performed, and e′ and a′ were extracted from tissue Doppler imaging curves, with corresponding ratios calculated. The pulsed-wave Doppler imaging of the aortic valve evaluated the LV ejection. Parameters were analyzed with Vevo Lab 2.2.0 software.

### cMRI

Mice were placed in a warmed animal bed and anesthetized with 1–2% v/v isoflurane in O_2_ delivered via a breathing mask. Breathing rate and body temperature were continuously monitored, and anesthesia depth was adjusted as needed. In vivo cMRI was performed at 9.4 T using a Bruker BioSpec 94/20 system equipped with a 1 T/m gradient system and ParaVision 5.1 software, including IntraGate for sequence acquisition and reconstruction. Data were acquired using a 35-mm volume coil. A stack of contiguous SAX slices (1-mm thickness) covering the entire RV and LV was obtained using the self-gated cine FLASH (IntraGate FLASH) sequence, as described^58^. For the analysis, regions of interest (ROIs) in end-diastolic and end-systolic frames of each SAX slice were manually defined in ImageJ v.1.54 (NIH) by tracing the epicardial and endocardial borders, as well as measuring ventricular and heart diameters and septal and posterior wall thickness. Left ventricular end-diastolic volume (LVEDV) and left ventricular end-systolic volume (LVESV) were calculated as the sum of the traced areas in each slice multiplied by slice thickness. SV, CO and LVEF were then derived. Outer diameter (OD) was measured from SAX images, and the Green–Lagrange strain (*E*) was quantified from the cyclic deformation of the myocardium at the mid-ventricular level during systole, on the basis of the circumferential dimensions of the endocardial contour at end-diastole (*C*_D_) and end-systole (*C*_S_), using the following equation

1 $$E=\frac{1}{2}\left({\left(\frac{{C}_{{\rm{S}}}}{{C}_{{\rm{D}}}}\right)}^{2}-1\right).$$

### IF and electron microscopy

IF and transmission electron microscopy (TEM) were performed following published protocols^59^. For IF microscopy, LV tissue samples were fixed in 4% paraformaldehyde (PFA) and 15% saturated picric acid and then dehydrated and embedded in paraffin. Sections (5–7-µm thick) were rehydrated, treated with peroxidase buffer and citrate–EGTA and blocked for 1 h in 5% bovine serum albumin containing 0.5% Triton X-100. Sections were incubated overnight at 4 °C with primary antibodies, followed by secondary antibodies. A full list of antibodies is provided in Supplementary Table 1. Images were taken with a Nikon Eclipse Ti2 confocal microscope.

At D6 and D13 post AAV9 injection, cleaved titin was detected using anti-cTEV and normalized to antimyotilin, a marker of both Z-disks and ICDs, using NIS Elements 4.3 software. Sarcomere number was determined by the semi-automated counting of M-band in anti-M-band titin (Ttn-M) confocal images (Nikon NIS v.4.3). The ICD width was measured on antimyotilin images in ImageJ v.1.54. WGA staining on tissue cross-sections delineated cell borders, allowing the semi-automated quantification of cardiomyocyte number per unit area and cardiomyocyte size using NIS v.4.3. Cycling cells were detected by anti-Ki67 staining; the mitotic index was calculated as the percentage of Ki67-positive nuclei relative to the total number of DAPI-stained nuclei. Cardiomyocytes were identified using anti-PCM1 and myofibroblasts by antiperiostin. The area stained by WGA in longitudinal sections was quantified in NIS v.4.3 and expressed as a fraction of total tissue area. Relative LC3B expression was normalized to antimyotilin signal. Apoptosis was assessed via TUNEL assay, with the apoptotic index calculated as the percentage of TUNEL-positive nuclei relative to total nuclei.

For TEM, LV tissue was fixed in 4% PFA, longitudinally sectioned into 50-μm slices, washed in PBS, dehydrated and embedded in resin. Ultrathin sections were cut from resin blocks, mounted on glow-discharged Formvar carbon-coated copper grids and imaged using a Zeiss LEO 910 transmission electron microscope. Images were acquired with a TRS SharpEye CCD camera (Troendle).

### Quantitative analysis of IF images

Confocal fluorescence images were analyzed using a custom MATLAB pipeline (R2024b, MathWorks). Spatial calibration was performed for each image using the embedded scale bar to convert pixel-based measurements into micrometers. Reference lines were manually placed along the ICD and subsequently refined by fitting a best-fit linear approximation of identical length to the segmented signal. Elliptical ROIs were generated around each reference line to define the relevant signal area. Signal segmentation was carried out using an ROI-based threshold determined by Otsu’s method applied to the intensity distribution within each ROI. The root mean square (RMS) deviation of segmented signal pixels from the corresponding reference line was calculated and reported as an absolute deviation in micrometers.

To quantify the intracellular versus extracellular fraction of integrin, the signal of interest was segmented using intensity-based thresholding and then classified according to its spatial relationship to a mask derived from the intracellular reference marker Z-disk titin (Ttn-Z). Signal pixels colocalizing with the Ttn-Z-derived mask were defined as intracellular, whereas noncolocalizing pixels were classified as extracellular. For each image, the relative intracellular and extracellular signal fractions were quantified as proportions of the total segmented signal. The classification accuracy was visually validated using overlay images.

### Quantification of Z-disk disorder

To quantify Z-disk disorder (‘fracture score’) in AAV9-injected hearts, we used a semi-automated program previously developed by our group^35^. Briefly, the TEM images of cardiac fiber bundles were filtered to isolate Z-disk structures. Each pixel along a Z-disk was assigned *x* and *y* coordinates and fitted to a best-fit straight line via linear regression. The residuals were weighted on the basis of angular differences between regression lines for all Z-disks in the *x*–*y* coordinate space. These values were averaged and further analyzed to derive the final fracture score.

### Immunohistochemistry

Ventricular tissue samples were fixed in 4% PFA and 15% saturated picric acid and then processed and embedded in paraffin. Sections (5–7-µm thick) were rehydrated and stained using a Picrosirius Red-Fast Green kit to visualize collagen fibers. Interstitial cardiac fibrosis was quantified as the collagen volume fraction using ImageJ v.1.54.

### SDS–PAGE and immunoblotting

Standard 10–15% SDS–PAGE gels, 1.8% titin gels and 1.8/7.5% two-phase gels were prepared following established protocols^21^. Proteins were visualized by Coomassie staining or identified by WB, as described^9,60^. Relative band intensities of N2BA/N2B titin on Coomassie-stained gels were used to calculate the percentage of titin cleaved by TEV. Antibodies used for WB are listed in Supplementary Table 1. WB signals were visualized via chemiluminescence (Amersham ECL Start, GE Healthcare), recorded with the ImageQuant LAS 4000 Imaging System (GE Healthcare) and quantified using MultiGauge v.3.0 (Fuji) or ImageQuant TL v.7.1 (GE Healthcare).

### Mechanical measurements

#### Isolated cardiomyocytes

For cardiomyocyte isolation, ventricular tissue from snap-frozen hearts was thawed in relaxing solution (170 mM K-propionate, 20 mM MOPS, 2.5 mM Mg-acetate, 5 mM K_2_EGTA, 2.5 mM ATP, 14.5 mM creatine phosphate, 1× protease inhibitor cocktail (Promega, G6521), pH 7.0 at 4 °C). The tissue was mechanically disrupted and permeabilized in relaxing solution containing 0.5% Triton X-100 for 8 min and then washed. Cardiomyocytes were mechanically analyzed as described^9^ and obtained from four Hom TEV, eight Het TEV and four Wt mouse hearts.

To measure the passive elastic force of single Het TC cardiomyocytes, permeabilized cells were mounted between a piezoelectric motor and a force transducer (Aurora Scientific, 403A) using shellac (120 mg ml^−1^ in 70% ethanol) on an Axiovert 135 inverted microscope (Carl Zeiss). Cells were stretched from slack length (~1.82-µm SL on average) to ~2.1-µm SL, held for 7 s and returned to slack. Elastic (quasi-steady state) force was calculated before and after TEV treatment by fitting the force-relaxation segment of raw traces with a one-phase exponential decay using GraphPad Prism v.9.

For active force measurements, permeabilized Wt and Het TC cardiomyocytes were mounted as above, adjusted to ~1.9 µm SL in relaxing solution and then sequentially exposed to the washing solution and pCa 5 solution (170 mM K-propionate, 10 mM MOPS, 2.40 mM Mg-acetate, 46.20 mM Ca-EGTA, 0.22 mM K_2_EGTA, pH 7, room temperature). The contraction was terminated with 20 µl K_2_EGTA (50 mM). Active force was defined as the difference between peak force in pCa 5 and post-EGTA force. The cell diameter was measured using 901D Hi-Speed Video Sarcomere Length v.4.195 (Aurora Scientific), and the force per area was calculated assuming circular cross-section. Active tension was normalized to Wt after TEV treatment.

To assess SL restoration after contraction, 200 µl of cell suspension was placed on a glass coverslip. After adhesion, relaxing solution was exchanged for washing buffer (185 mM K-propionate, 20 mM MOPS, 2.5 mM Mg-acetate, 2.5 mM ATP, pH 7, room temperature), followed by activation with pCa 6 solution (170 mM K-propionate, 10 mM MOPS, 2.42 mM Mg-acetate, 31.60 mM Ca-EGTA, 1.72 mM K_2_EGTA, pH 7, room temperature). Contraction was stopped with 20 µl K_2_EGTA (50 mM). The solution was then changed to relaxing solution with or without in-house-produced TEV^9^. After 20–30 min at room temperature, the washing solution was added and a second contraction induced, followed by EGTA relaxation. The SL was measured after each solution change using 901D Hi-Speed Video Sarcomere Length v.4.195 (Aurora Scientific).

#### Cardiac fibers

Thawed hearts were cut open and permeabilized in relaxing solution with 0.5% Triton X-100 for 16 h at 4 °C, then washed. Trabeculae were dissected from the LV wall under a stereo microscope and secured with surgical suture on ice. Each bundle was mounted between a piezo motor and force transducer using aluminum clamps (Scientific Instruments)^9^. Dimensions were recorded at slack length (~1.8-µm SL), with lengths of 1.0–2.5 mm and widths of 0.6–1.8 mm. Bundles were suspended in the relaxing solution at room temperature and subjected to a five-step passive stretch protocol up to 20% strain. Force data were acquired at 1,000 Hz using custom software (https://github.com/DrDJIng/FiberStretchProgram). Peak force values at each strain step were normalized to the (circular) cross-sectional area estimated from bundle diameter. Trabeculae were isolated from six hearts (two each from Hom TEV D6, D13 and Hom GFP).

### Quantitative proteome analysis

Proteomic analyses were performed using the following number of animals: at D6 post injection, *N* = 6 (Hom TEV), *N* = 6 (Het GFP) and *N* = 3 (Hom GFP); at D13, *N* = 6 (Hom TEV and GFP). For D6 analysis, HET GFP and Hom GFP were pooled owing to only minor differences between them.

#### In-solution digestion and peptide purification

Heart tissue was homogenized in 9 M urea with 0.3% Triton X-100 and protease/phosphatase inhibitors (Halt). Lysates were sonicated, centrifuged at 17,000*g* for 5 min and the pellet discarded. Triton was removed from 100 µg of clarified lysate via chloroform-methanol precipitation, and the pellet resuspended in 100 µl 9 M urea. Samples were reduced with 5 mM dithiothreitol (45 min, room temperature) and alkylated with 10 mM iodoacetamide (30 min, dark). The solution was diluted with 450 µl 50 mM Tris–HCl (pH 8.0) to lower the urea concentration to <2 M. Trypsin/Lys-C Protease Mix (2 µg; Thermo Fisher, A41007) was added, and digestion performed at 37 °C with shaking (600 rpm) for 16–18 h. Digestion was stopped by adjusting the pH to ≤3 with trifluoroacetic acid. Samples were dried in a vacuum centrifuge and peptides desalted using C18 spin columns (G Biosciences).

#### Liquid chromatography

Desalted peptides were dried, resuspended in buffer A (0.1% formic acid) and loaded onto a Vanquish Neo UHPLC system (Thermo Fisher) using a heated trap-and-elute setup with a PepMap C18 trap column (5 mm, P/N 160454) in forward-flush mode. Separation was carried out on a 50 cm Easy-Spray PepMap Neo 2 µm C18 analytical column (75 µm × 500 mm, P/N ES75500PN) at 40 °C using a 150-min gradient with buffer B (80% acetonitrile, 0.1% formic acid): 4–5% (5 min), 5–35% (110 min), 35–65% (25 min), 65–99% (3 min) and then held at 99% (12 min).

Mass spectra were acquired on an Orbitrap Eclipse Tribrid with FAIMS Pro (Thermo Fisher) using Tune 3.5 and Xcalibur 4.5. Acquisition parameters: spray voltage 1,600 V, ion transfer tube 300 °C, FAIMS CVs −45, −55, −65 V (1.5-s cycle). MS1 scans: Orbitrap, 120,000 resolution, 375–1,600 *m*/*z*, AGC 300%, max injection time 50 ms, S-lens RF 30, monoisotopic precursor selection (2–7 charges), dynamic exclusion (60 s, ±10 ppm). Data-dependent MS2 scans: quadrupole isolation (1.6 *m*/*z*), HCD at 30%, detection in ion trap (Turbo scan rate), AGC target 10,000, 35 ms maximum injection, one microscan, centroid mode.

#### Tandem mass spectrometry data analysis and processing

Raw data were analyzed using Proteome Discoverer 2.5 (Thermo Fisher) with the Sequest HT search engine against UniProt-proteome_UP000000589. Search parameters: 10 ppm precursor and 0.02 Da fragment tolerance; up to two missed cleavages; variable Ox(M) and Ac(N-term); fixed Cam(C). PSMs were validated by Percolator (false discovery rate 0.01/0.05, ΔCn ≤ 0.05, *q* value). Quantification was based on Unique+Razor peptides, with intensities normalized to total peptide signal and summed per protein for ratio calculation. A summary of results is provided in Supplementary Table 2.

Raw mass spectrometry data were deposited in the MassIVE data repository (https://massive.ucsd.edu/ProteoSAFe/static/massive.jsp; ID MSV000097567), part of the ProteomeXchange Consortium (https://www.proteomexchange.org/), and are directly accessible via https://massive.ucsd.edu/ProteoSAFe/dataset.jsp?task=92e03a238c28452e913fff91a5253a8f.

#### Bioinformatics and data visualization

Principal component analysis was conducted using Perseus software (v.1.6.15.0). GO and KEGG pathway enrichment analyses were performed using DAVID v.2021 (knowledgebase v2023q4)^61,62^, with the default *Mus musculus* background gene list. Data visualization included volcano plots and GO biological process enrichment plots generated in GraphPad Prism v.9 and combined GO BP, MF, CC and KEGG visualizations created in R (v.4.4.2) using the ggplot2 package. Venn diagrams were generated using the online tool from the Van de Peer laboratory (http://bioinformatics.psb.ugent.be/webtools/Venn). Network analysis details are described below.

### Transcriptomics

LV tissue (~10 mg) was collected at D6 and D13 post injection, snap-frozen and submitted to Novogene for RNA sequencing. Total RNA was extracted using Trizol reagent. RNA quality was assessed by 1% agarose gel electrophoresis and analyzed on a Bioanalyzer 2100 (Agilent Technologies). Ribosomal RNA (rRNA) was removed, and rRNA-free samples were purified by ethanol precipitation. Sequencing libraries were prepared from rRNA-depleted RNA by fragmentation, followed by first-strand cDNA synthesis using random hexamers and second-strand synthesis substituting dUTP with dTTP. The resulting directional libraries were completed by end repair, A-tailing, adapter ligation, size selection, USER enzyme digestion, PCR amplification and final purification. Libraries were quantified, pooled and sequenced on the Illumina NovaSeq X Plus platform using V1.5 reagents and S4 flow cell with paired-end 150-bp reads, targeting 40 million reads per sample.

#### Bioinformatics

Data processing was performed by Novogene. Cleaned paired-end reads were aligned to the *Mus musculus* reference genome (GRCm38) using Hisat2 v.2.0.5. The differential gene expression between TEV- and GFP-injected samples was analyzed using DESeq2 v.1.20.0. For the D6 dataset, Hom GFP (*N* = 3) and Het GFP (*N* = 6) samples were pooled and compared with Hom TEV (*N* = 6). For the D13 dataset, Hom TEV (*N* = 6) was compared with Hom GFP (*N* = 6). The GO enrichment and KEGG pathway analyses were performed using ClusterProfiler v.3.8.1. An adjusted *P* value of <0.05 was considered statistically significant for enrichment. Data visualization was conducted in GraphPad Prism v.9 and R using the ggplot2 package.

#### Network analysis

Differentially expressed gene lists comparing TEV- with GFP-injected hearts at both D6 and D13, from transcriptomic and proteomic datasets, were analyzed with Metascape^63^ (https://metascape.org). The resulting network diagrams were visualized in Cytoscape, where each enriched term was represented by a circular node with a pie chart indicating the proportion of genes contributing from each omics dataset. The gene count for each node was normalized to the total number of genes detected in that specific omics analysis. Nodes with a similarity score >0.3 were connected by edges, indicating biological or functional similarity between terms.

### Statistical analysis

Data organization, visualization and analysis were performed using Microsoft Excel 2021 and GraphPad Prism (v.9 or v.10). Results are presented as mean ± s.e.m., unless noted (Supplementary Fig. 4d shows median ± 95% CI). Normality was assessed using, at a minimum, the Shapiro–Wilk test. For two-group comparisons, the two-tailed *t*-test (paired or unpaired) was used for normal data or Mann–Whitney *U* test for non-normal data. For multiple groups, one-way analysis of variance (ANOVA) with Tukey’s post hoc test was applied for normal data, or Kruskal–Wallis with Dunn’s test for nonparametric data. For repeated measures, two-way ANOVA (mixed-effects model) was used, followed by Šidák’s or Tukey’s test. Significance was set at *P* < 0.05.

### Reporting summary

Further information on research design is available in the Nature Portfolio Reporting Summary linked to this article.

## Supplementary information

Reporting summary

Peer Review File

Supplementary Movie 1 This movie shows long-axis cMRI frames recorded from a TC mouse injected with control AAV9–GFP on D6 post injection.

Supplementary Movie 2 This movie shows long-axis cMRI frames recorded from a TC mouse injected with AAV9-TEV on D6 post injection.

Supplementary Movie 3 This movie shows long-axis cMRI frames recorded from a TC mouse injected with AAV9-TEV on D13 post injection.

Supplementary Table 1 Antibodies, dyes and staining kits.

Supplementary Table 2 Summary of our proteomic analysis of TC mouse hearts injected with TEV or GFP on D6 and D13 post injection.

## Source data

Source Data Fig. 1 Statistical source data.

Source Data Fig. 2 Statistical source data.

Source Data Fig. 3 Statistical source data.

Source Data Fig. 4 Statistical source data.

Source Data Fig. 5 Statistical source data.

Source Data Fig. 6 Statistical source data.

Source Data Figs. 1, 3, 5 and 6 Unprocessed WBs and gels for Figs. 1d,e,f,g,i, 3a, 5g and 6d,f,h.

Source Data Extended Data Fig. 1 Statistical source data.

Source Data Extended Data Fig. 2 Statistical source data.

Source Data Extended Data Fig. 3 Statistical source data.

Source Data Extended Data Fig. 4 Statistical source data.

Source Data Extended Data Fig. 5 Statistical source data.

Source Data Extended Data Fig. 6 Statistical source data.

Source Data Extended Data Fig. 7 Statistical source data.

Source Data Extended Data Figs. 1, 4 and 7 Unprocessed WBs and gels for Extended Data Figs. 1c,d, 4a,b and 7b,d,e,f,g.

## Data Availability

All data are available in the article or its Supplementary Information. Raw mass spectrometry data were deposited in the MassIVE data repository (https://massive.ucsd.edu/ProteoSAFe/static/massive.jsp; ID MSV000097567), part of the ProteomeXchange Consortium (https://www.proteomexchange.org/), and are directly accessible at https://massive.ucsd.edu/ProteoSAFe/dataset.jsp?task=92e03a238c28452e913fff91a5253a8f.

## Code availability

The Z-disk fracture score was calculated using custom software available via GitHub at https://github.com/UKMPhysII/QuantitativeFractureCode. Fiber mechanics were performed using a custom stretch protocol available via GitHub at https://github.com/DrDJIng/FiberStretchProgram.
