# Supplementary material for: Selective titin cleavage disrupts cardiac mechanical homeostasis to drive heart failure and fibrosis
Source: Nat Cardiovasc Res. 2026 Jun 16;5(6):572–87. doi: 10.1038/s44161-026-00829-z (PMC13271893; doi:10.1038/s44161-026-00829-z)
Supplement: Supplementary file 1 — Reporting summary [file 44161_2026_829_MOESM1_ESM.pdf]

nature portfolio

Corresponding author(s): Wolfgang A. Linke

Last updated by author(s): May 12, 2026

Reporting Summary

Nature Portfolio wishes to improve the reproducibility of the work that we publish. This form provides structure for consistency and transparency in reporting. For further information on Nature Portfolio policies, see our Editorial Policies and the Editorial Policy Checklist.

Statistics

For all statistical analyses, confirm that the following items are present in the figure legend, table legend, main text, or Methods section.

n/a	Confirmed	
☐	☒	The exact sample size (*n*) for each experimental group/condition, given as a discrete number and unit of measurement
☐	☒	A statement on whether measurements were taken from distinct samples or whether the same sample was measured repeatedly
☐	☒	The statistical test(s) used AND whether they are one- or two-sided<br>*Only common tests should be described solely by name; describe more complex techniques in the Methods section.*
☒	☐	A description of all covariates tested
☐	☒	A description of any assumptions or corrections, such as tests of normality and adjustment for multiple comparisons
☐	☒	A full description of the statistical parameters including central tendency (e.g. means) or other basic estimates (e.g. regression coefficient) AND variation (e.g. standard deviation) or associated estimates of uncertainty (e.g. confidence intervals)
☐	☒	For null hypothesis testing, the test statistic (e.g. *F*, *t*, *r*) with confidence intervals, effect sizes, degrees of freedom and *P* value noted<br>*Give P values as exact values whenever suitable.*
☒	☐	For Bayesian analysis, information on the choice of priors and Markov chain Monte Carlo settings
☒	☐	For hierarchical and complex designs, identification of the appropriate level for tests and full reporting of outcomes
☒	☐	Estimates of effect sizes (e.g. Cohen's *d*, Pearson's *r*), indicating how they were calculated

Our web collection on statistics for biologists contains articles on many of the points above.

Software and code

Policy information about availability of computer code

Data collection	Chemiluminescence on western blots and Coomassie stained gels were recorded using the Image Quant software v7.1 of the ImageQuant LAS 4000 Imaging System (GE Healthcare). Stained tissue sections were imaged with NIS Elements Software v4.3 provided with the Nikon DS-Fi2/DS-U3 camera. Electron microscopic images were taken with a TRS sharpeye CCD Camera and manufacturer's software ImageSP (Troendle, Moorenweis, Germany).<br>Passive tension measurements of cardiomyocytes were performed using the manufacturer's software on a setup by Aurora Scientific. Sarcomere length and cell diameter of isolated cardiomyocytes was measured using 901D Hi-Speed Video Sarcomere Length v.4.195 (Aurora Scientific) software.<br>Force data of cardiac fibres were acquired at 1000 Hz using custom software (https://github.com/DrDJIng/FiberStretchProgram).<br>Transthoracic echocardiography were recorded using Vevo 2100 (MS550D, 18-55 MHz).<br>In vivo cMRI was performed at 9.4 T using a Bruker BioSpec 94/20 system (Ettlingen, Germany) with a 1 T/m gradient system and ParaVision 5.1 software.
Data analysis	Chemiluminescence signal intensity was quantified using the ImageQuant TL software v7.1 (GE Healthcare) or MultiGauge v.3.0 (Fuji). Cardiac magnetic resonance images, Immunofluorescence images and Immunohistochemistry images were analyzed by ImageJ 1.54 software (NIH Bethesda) or by NIS Elements Software v4.3 (Nikon).<br>Transthoracic echocardiography parameters were analysed with Vevo Lab 2.2.0 software.<br>Data organization, scientific graphing and statistical analyses were performed using Microsoft Excel 2021 and GraphPad Prism 9 or 10 software. Quantification of Z-disk disorder was done using MATLAB with the Quantitative Fracture Code (https://github.com/UKMPhysII/QuantitativeFractureCode). Confocal fluorescence images were analyzed using a custom MATLAB pipeline (R2024b, MathWorks).<br>Principal component analysis was performed using Perseus software (v.1.6.15.0). Gene ontology and Kyoto Encyclopedia of Genes and Genomes pathway enrichment analyses were conducted using DAVID v.2021.

Differential expression analysis was performed using DESeq2 v.1.20.0. GO enrichment analysis and KEGG pathway analysis were implemented by ClusterProfiler v.3.8.1. Network analysis was performed using Metascape.

For manuscripts utilizing custom algorithms or software that are central to the research but not yet described in published literature, software must be made available to editors and reviewers. We strongly encourage code deposition in a community repository (e.g. GitHub). See the Nature Portfolio guidelines for submitting code & software for further information.

Data

Policy information about availability of data

All manuscripts must include a data availability statement. This statement should provide the following information, where applicable:

- Accession codes, unique identifiers, or web links for publicly available datasets
- A description of any restrictions on data availability
- For clinical datasets or third party data, please ensure that the statement adheres to our policy

All data pertaining to this work are shown in the text, figures, and Supplementary Information. The raw mass spectrometry data has been uploaded to the MassIVE data repository (https://massive.ucsd.edu/ProteoSAFe/static/massive.jsp).

Research involving human participants, their data, or biological material

Policy information about studies with human participants or human data. See also policy information about sex, gender (identity/presentation), and sexual orientation and race, ethnicity and racism.

Reporting on sex and gender	*Use the terms sex (biological attribute) and gender (shaped by social and cultural circumstances) carefully in order to avoid confusing both terms. Indicate if findings apply to only one sex or gender; describe whether sex and gender were considered in study design; whether sex and/or gender was determined based on self-reporting or assigned and methods used. Provide in the source data disaggregated sex and gender data, where this information has been collected, and if consent has been obtained for sharing of individual-level data; provide overall numbers in this Reporting Summary. Please state if this information has not been collected. Report sex- and gender-based analyses where performed, justify reasons for lack of sex- and gender-based analysis.*
Reporting on race, ethnicity, or other socially relevant groupings	*Please specify the socially constructed or socially relevant categorization variable(s) used in your manuscript and explain why they were used. Please note that such variables should not be used as proxies for other socially constructed/relevant variables (for example, race or ethnicity should not be used as a proxy for socioeconomic status). Provide clear definitions of the relevant terms used, how they were provided (by the participants/respondents, the researchers, or third parties), and the method(s) used to classify people into the different categories (e.g. self-report, census or administrative data, social media data, etc.) Please provide details about how you controlled for confounding variables in your analyses.*
Population characteristics	*Describe the covariate-relevant population characteristics of the human research participants (e.g. age, genotypic information, past and current diagnosis and treatment categories). If you filled out the behavioural & social sciences study design questions and have nothing to add here, write "See above."*
Recruitment	*Describe how participants were recruited. Outline any potential self-selection bias or other biases that may be present and how these are likely to impact results.*
Ethics oversight	*Identify the organization(s) that approved the study protocol.*

Note that full information on the approval of the study protocol must also be provided in the manuscript.

Field-specific reporting

Please select the one below that is the best fit for your research. If you are not sure, read the appropriate sections before making your selection.

☒ Life sciences ☐ Behavioural & social sciences ☐ Ecological, evolutionary & environmental sciences

For a reference copy of the document with all sections, see nature.com/documents/nr-reporting-summary-flat.pdf

Life sciences study design

All studies must disclose on these points even when the disclosure is negative.

Sample size	The number of animals was calculated by Resource Equation methods with error degrees of freedom E greater than or equal to 40. (Mead R (1988). The Design of Experiments. Cambridge, New York: Cambridge University Press. Festing MFW, Overend P, Das RG, Borja MC, Berdoy M (2002). The Design of Animal Experiments. The Royal Socie-ty of Medicine Press Limited; Laboratory Animal Handbooks No. 14). For in vitro experiments no specific sample size calculation was undertaken before experiments. We chose sample size according to standard practice in the field of muscle physiology, which suggested that in most cases, n=5-7 animals/group are sufficient for characterization of differences in genotype, phenotype, and specific heart or muscle properties (e.g., DOI: 10.1161/CIRCULATIONAHA.106.645499 and DOI: 10.1126/science.aaa5458).

Data exclusions	No data were excluded.
Replication	The in vivo experiments on adult mice were performed on 5-13 individual animals/group. Post mortem tissue analyses were performed on tissues from at least 2 individual mice/group. The number of in vitro experiments and number of biologically independent samples are mentioned in the figure legends. All data shown could be reproduced in multiple repeats, as indicated in the figure legends.
Randomization	For the mouse studies, littermates were grouped by genotypes (WT, Het, and Hom). From each litter, mice were simultaneously treated with either the TEV vector or the control vector.
Blinding	Investigators were not blinded. Blinded analysis was not done, because in most experiments genotypes were immediately recognized by phenotypic differences. Likewise, protein lysates from AAV9-TEV and AAV9-GFP injected animals were readily distinguished by microscopical images of cleaved titin signals and ultrastructural changes. These obvious differences hindered blinding and made it less relevant.

Reporting for specific materials, systems and methods

We require information from authors about some types of materials, experimental systems and methods used in many studies. Here, indicate whether each material, system or method listed is relevant to your study. If you are not sure if a list item applies to your research, read the appropriate section before selecting a response.

Materials & experimental systems

n/a	Involved in the study
☐	☒ Antibodies
☐	☒ Eukaryotic cell lines
☒	☐ Palaeontology and archaeology
☐	☒ Animals and other organisms
☒	☐ Clinical data
☒	☐ Dual use research of concern
☒	☐ Plants

Methods

n/a	Involved in the study
☒	☐ ChIP-seq
☒	☐ Flow cytometry
☒	☐ MRI-based neuroimaging

Antibodies

Antibodies used

A complete list containing full information about the antibodies is provided in Supplementary Table S1. For all antibodies, we have provided the supplier name, catalog number and dilution.

Anti-Alpha B Crystallin, mouse monoclonal, Abcam, Cat. #AB13496, WB '(1:4000)'
Anti-CHIP/ STUB1, rabbit monoclonal, Abcam, Cat. #AB134064, WB '(1:5000)'
Anti-Connexin 43, rabbit polyclonal, Abcam, Cat. #AB11370, IF '(1:100)'
Anti-Cronos, rabbit polyclonal, Eurogentec Custom-made, WB '(1:2000)'
Anti-Desmin D33, mouse monoclonal, Dako, Cat. #M 0760, IF '(1:100)'
Anti-Desmoplakin, mouse monoclonal, Progen, Cat. #610035, IF '(1:5)'
Anti-FBXO32 (Atrogin1), rabbit polyclonal, St John's Laboratory, Cat. #STJ23637, WB '(1:1000)'
Anti-GAPDH (14C10), rabbit monoclonal, Cell Signaling, Cat. #2118, WB '(1:40000)'
Anti-GFP, monoclonal rabbit, Cell Signaling, Cat. #2956, WB '(1:1000)'
Anti-HSP27, rabbit monoclonal, DSHB, Cat. #CPTC-HSPB 1-4, WB '(1:1000)'
Anti-HSP90, rabbit polyclonal, Cell Signaling, Cat. #4874, WB '(1:1000)'
Anti-Integrin α5β1, rat monoclonal, Merck, Cat. #MAB2514, IF '(1:100)'
Anti-Integrin β1d, mouse monoclonal, Abcam, Cat. #AB8991, WB '(1:1000)'
Anti-Ki67, rabbit polyclonal, Abcam, Cat. #ab15580, IF '(1:250)'
Anti-LC3B, rabbit polyclonal, Cell Signaling, Cat. #2775, IF '(1:200)', WB '(1:2500)'
Anti-MuRF1 (C-11), mouse monoclonal, Santa Cruz, Cat. #sc-398608, WB '(1:10000)'
Anti-Myotilin (Leica NLC Myotilin), mouse monoclonal, ThermoFisher Scientific, Cat. #50-255-2199, IF '(1:100)'
Anti-N-Cadherin, mouse monoclonal, Invitrogen, Cat. #33-3900, IF '(1:100)'
Anti-p62, rabbit monoclonal, Abcam, Cat. #AB109012, WB '(1:2500)'
Anti-Pan-Ubiquitin P4D1, mouse monoclonal, Santa Cruz, Cat. #sc-8017, WB '(1:100)'
Anti-PCM1, rabbit polyclonal, Merck Millipore, Cat. #HPA 023374, IF '(1:250)'
Anti-Periostin, mouse monoclonal, Proteintech, Cat. #66491-I-Ig, IF '(1:1000)', WB '(1:4000)'
Anti-PDGFRα, rabbit monoclonal, Abcam, Cat. #ab203491, IF '(1:250)'
Anti-TEV, rabbit polyclonal, ThermoFisher Scientific, Cat. #200-401-B91, WB '(1:500)'
Anti-TEV cleavage site (cTEV), rabbit polyclonal, ThermoFisher Scientific, Cat. #PA1-119, IF '(1:500)', WB '(1:1000)'
Anti-Titin I20-22, rabbit polyclonal, Myomedix, Cat. #TTN-5, IF '(1:400)', WB '(1:2000)'
Anti-Titin M, rabbit polyclonal, Myomedix, Cat. #TTN-9, IF '(1:100)'
Anti-Titin MIR, rabbit polyclonal, Myomedix, Cat. #TTN-6, WB '(1:20000)'
Anti-Titin Z, rabbit polyclonal, Myomedix, Cat. #TTN-1, WB '(1:10000)'

Validation

All commercially available antibodies are commonly used and were validated by the suppliers, as documented below.

Anti-Alpha B Crystallin, [Abcam ab13496] (https://www.abcam.com/en-us/products/primary-antibodies/alpha-b-crystallin-

antibody-1b61-3g4-ab13496?utm_source=chatgpt.com)
Anti-CHIP/STUB1, [Abcam ab134064] (https://www.abcam.com/products/primary-antibodies/stub1-chip-antibody-epr4447-ab134064.html?utm_source=chatgpt.com)
Anti-Connexin 43, [Abcam ab11370] (https://www.abcam.com/en-us/products/primary-antibodies/connexin-43-gja1-antibody-intercellular-junction-marker-ab11370?utm_source=chatgpt.com)
Anti-Cronos, (Custom-made) [Eurogentec Custom Antibodies] (https://www.eurogentec.com/en/custom-antibody-production.html?utm_source=chatgpt.com)
Anti-Desmin D33, [Dako M0760] (https://www.agilent.com/en/product/immunohistochemistry/primary-antibodies/desmin-clone-d33-m0760?utm_source=chatgpt.com)
Anti-Desmoplakin, [PROGEN 610035] (https://www.progen.com/en/desmoplakin-antibody-dp-1-dp-2-610035.html?utm_source=chatgpt.com)
Anti-FBXO32 (Atrogin1), [St John's STJ23637] (https://www.stjohnslabs.com/anti-fbxo32-antibody-stj23637/?utm_source=chatgpt.com)
Anti-GAPDH (14C10), [Cell Signaling 2118] (https://www.cellsignal.com/products/primary-antibodies/gapdh-14c10-rabbit-mab/2118?utm_source=chatgpt.com)
Anti-GFP, [Cell Signaling 2956] (https://www.cellsignal.com/products/primary-antibodies/gfp-d5-1-xp-rabbit-mab/2956?utm_source=chatgpt.com)
Anti-HSP27, [DSHB CPTC-HSPB1-4] (https://dshb.biology.uiowa.edu/CPTC-HSPB1-4?utm_source=chatgpt.com)
Anti-HSP90, [Cell Signaling 4874] (https://www.cellsignal.com/products/primary-antibodies/hsp90-antibody/4874?utm_source=chatgpt.com)
Anti-Integrin α5β1, [Merck MAB2514] (https://www.emdmillipore.com/DE/de/product/Anti-Integrin-alpha5beta1-Antibody-clone-BIIA1,MM_NF-MAB2514?utm_source=chatgpt.com)
Anti-Integrin β1d, [Abcam ab8991] (https://www.abcam.com/products/primary-antibodies/integrin-beta-1d-antibody-ab8991.html?utm_source=chatgpt.com)
Anti-Ki67, [Abcam ab15580] (https://www.abcam.com/products/primary-antibodies/ki67-antibody-ab15580.html?utm_source=chatgpt.com)
Anti-LC3B, [Cell Signaling 2775] (https://www.cellsignal.com/products/primary-antibodies/lc3b-antibody/2775?utm_source=chatgpt.com)
Anti-MuRF1 (C-11), [Santa Cruz sc-398608] (https://www.scbt.com/p/murf1-antibody-c-11-sc-398608?utm_source=chatgpt.com)
Anti-Myotilin (Leica NCL Myotilin), [Thermo Fisher 50-255-2199] (https://www.thermofisher.com/antibody/product/Myotilin-Antibody-clone-MYOT-1-Monoclonal/50-255-2199?utm_source=chatgpt.com)
Anti-N-Cadherin, [Invitrogen 33-3900] (https://www.thermofisher.com/antibody/product/N-Cadherin-Antibody-clone-32-NCAD-Monoclonal/33-3900?utm_source=chatgpt.com)
Anti-p62, [Abcam ab109012] (https://www.abcam.com/products/primary-antibodies/p62-antibody-epr4844-ab109012.html?utm_source=chatgpt.com)
Anti-Pan-Ubiquitin P4D1, [Santa Cruz sc-8017] (https://www.scbt.com/p/ubiquitin-antibody-p4d1-sc-8017?utm_source=chatgpt.com)
Anti-PCM1, [Merck HPA023374] (https://www.sigmaaldrich.com/DE/en/product/sigma/hpa023374?utm_source=chatgpt.com)
Anti-Periostin, [Proteintech 66491-I-Ig](https://www.ptglab.com/products/Periostin-Antibody-66491-1-Ig.htm?utm_source=chatgpt.com)
Anti-PDGFRα, [Abcam ab203491] (https://www.abcam.com/products/primary-antibodies/pdgfr-alpha-antibody-epr19598-ab203491.html?utm_source=chatgpt.com)
Anti-TEV, [Thermo Fisher 200-401-B91] (https://www.thermofisher.com/antibody/product/TEV-Protease-Antibody-Polyclonal/200-401-B91?utm_source=chatgpt.com)
Anti-TEV cleavage site (cTEV), [Thermo Fisher PA1-119] (https://www.thermofisher.com/antibody/product/TEV-Protease-Cleavage-Site-Antibody-Polyclonal/PA1-119?utm_source=chatgpt.com)
Anti-Titin I20-22, [Myomedix TTN-5] (http://www.myomedix.com/seiten/styled/index.html)
Anti-Titin M, [Myomedix TTN-9] (http://www.myomedix.com/seiten/styled/index.html)
Anti-Titin MIR, [Myomedix TTN-6] (http://www.myomedix.com/seiten/styled/index.html)
Anti-Titin Z, [Myomedix TTN-1] (http://www.myomedix.com/seiten/styled/index.html)

Eukaryotic cell lines

Policy information about cell lines and Sex and Gender in Research

Cell line source(s)
AAV-293 (HEK-293) cell line is commercially available from Stratagene/Agilent (https://www.integratedsci.com.au/product/aav-293-cells.html)

Authentication
The cell line was not authenticated because it was obtained commercially.

Mycoplasma contamination
Cells were tested negative for mycoplasma contamination.

Commonly misidentified lines
(See ICLAC register)
No commonly misidentified cell lines were used in the study.

Animals and other research organisms

Policy information about studies involving animals; ARRIVE guidelines recommended for reporting animal research, and Sex and Gender in Research

Laboratory animals
Mus musculus of background strain C57BL/6JRj were used, aged 8-44 weeks, both male and female. Genetic modifications are as indicated in the paper. Mice were singly housed in ventilated cages at approximately 22 degrees Celsius with 12-hour light/dark cycles, food and water ad libitum.

Wild animals	none
Reporting on sex	Sex-based analyses of in vivo data were performed as described in the results section (Suppl. Fig. 2).
Field-collected samples	none
Ethics oversight	All experimental procedures were approved by the Animal Care and Use Committees of North Rhine-Westphalia, Germany (LAVE NRW, 81-02.04.2019.A472).

Note that full information on the approval of the study protocol must also be provided in the manuscript.

Plants

Seed stocks	none
Novel plant genotypes	none
Authentication	none
