# Supplementary material for: Selective titin cleavage disrupts cardiac mechanical homeostasis to drive heart failure and fibrosis
Source: Nat Cardiovasc Res. 2026 Jun 16;5(6):572–87. doi: 10.1038/s44161-026-00829-z (PMC13271893; doi:10.1038/s44161-026-00829-z)

Fig. 1D

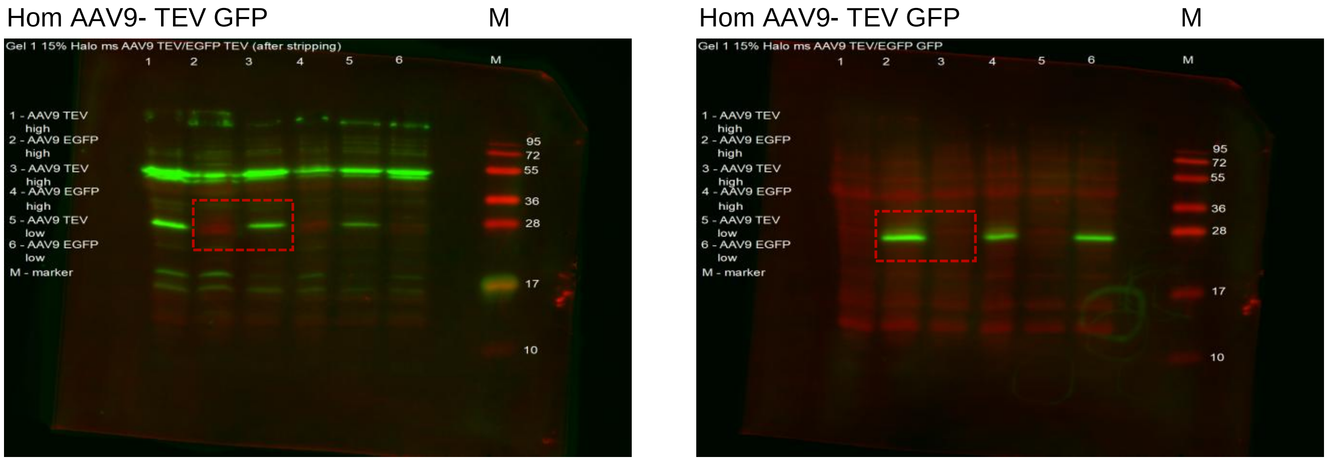
Hom AAV9- TEV GFP
M
Gel 1 15% Halo ms AAV9 TEV/EGFP TEV (after stripping)
1 2 3 4 5 6 M
1 - AAV9 TEV high
2 - AAV9 EGFP high
3 - AAV9 TEV high
4 - AAV9 EGFP high
5 - AAV9 TEV low
6 - AAV9 EGFP low
M - marker
95
72
55
36
28
17
10
Hom AAV9- TEV GFP
M
Gel 1 15% Halo ms AAV9 TEV/EGFP GFP
1 2 3 4 5 6 M
1 - AAV9 TEV high
2 - AAV9 EGFP high
3 - AAV9 TEV high
4 - AAV9 EGFP high
5 - AAV9 TEV low
6 - AAV9 EGFP low
M - marker
95
72
55
36
28
17
10

Fig. 1E

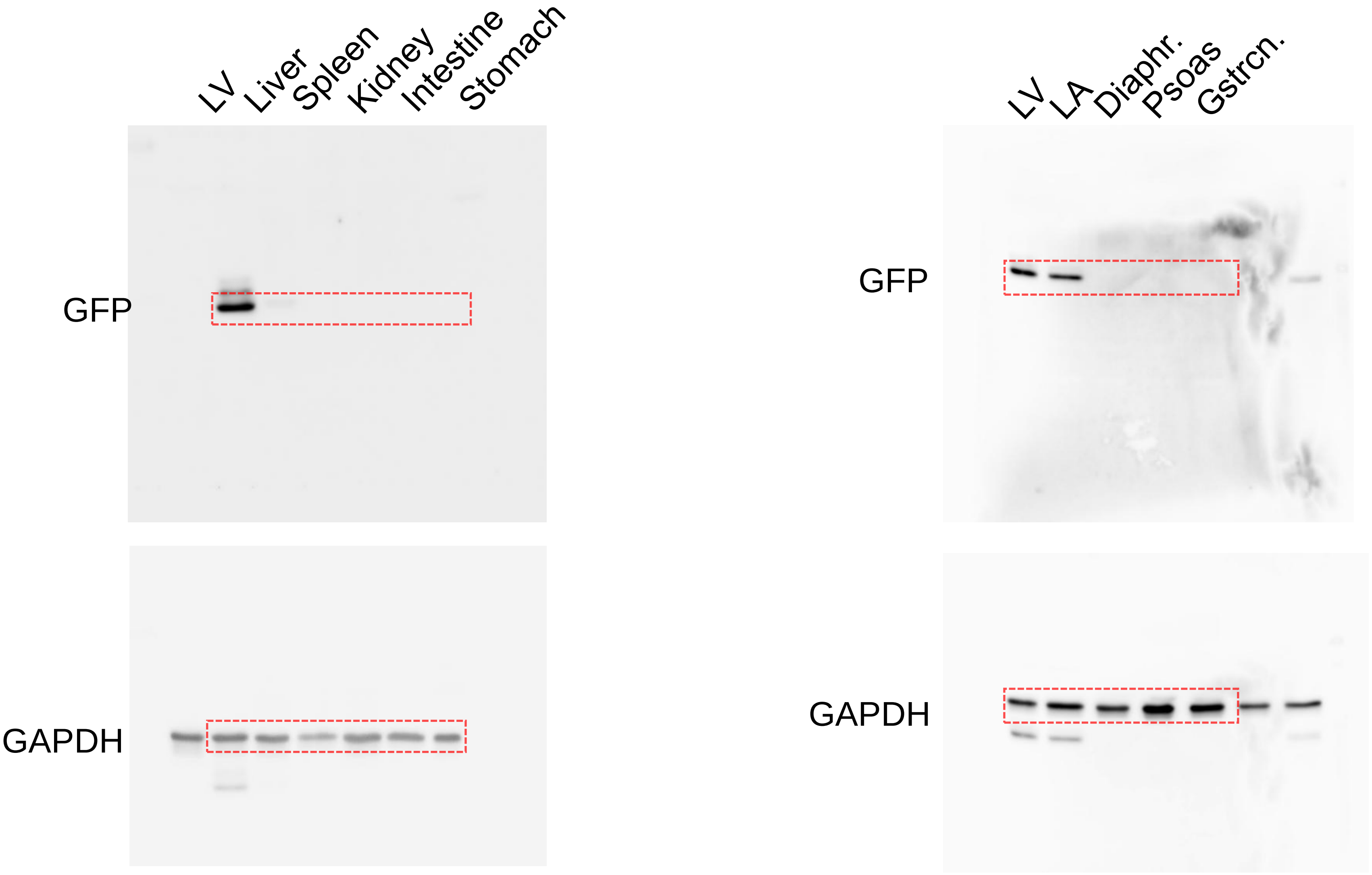
LV
Liver
Spleen
Kidney
Intestine
Stomach
GFP
GAPDH
LV
LA
Diaphr.
Psoas
Gstrcn.
GFP
GAPDH

Fig. 1F

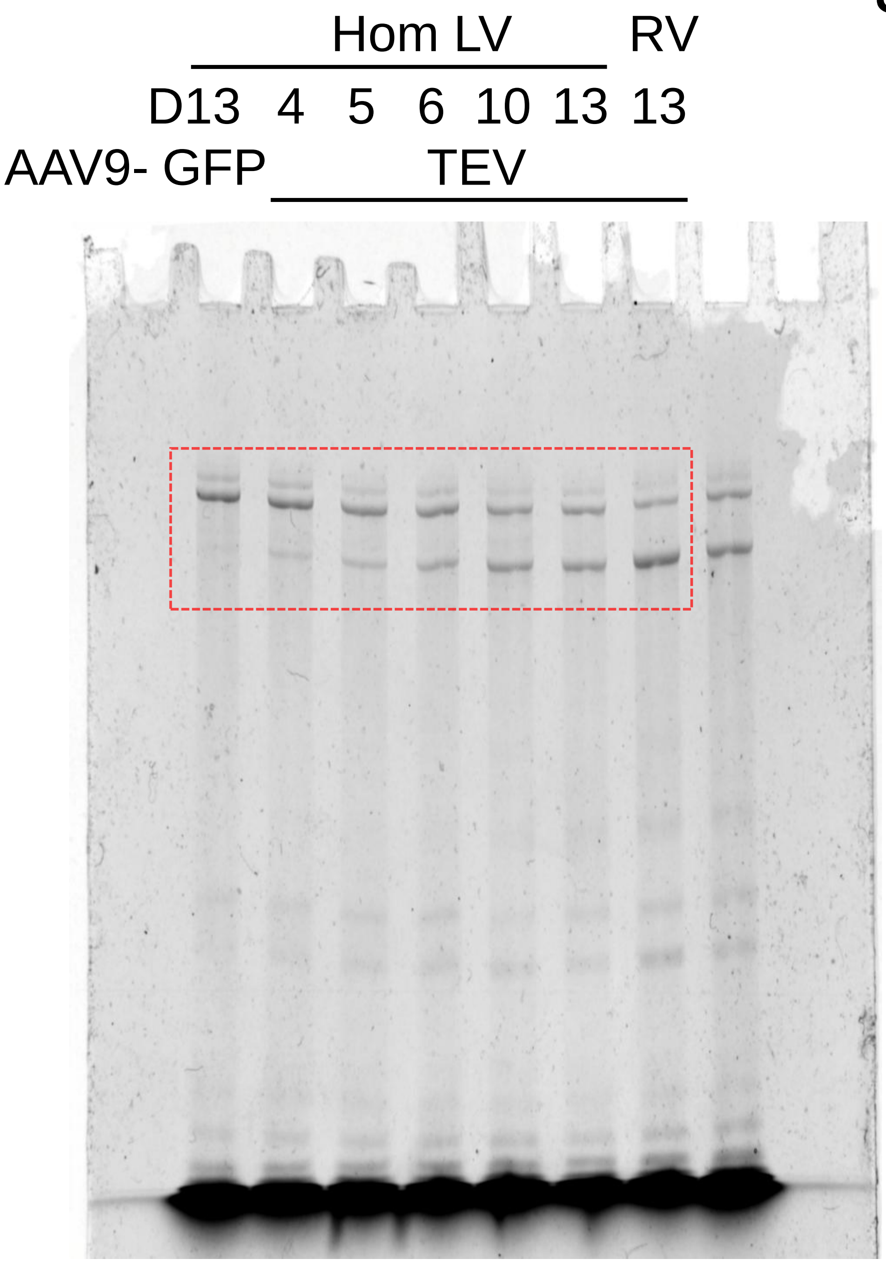

Fig. 1G

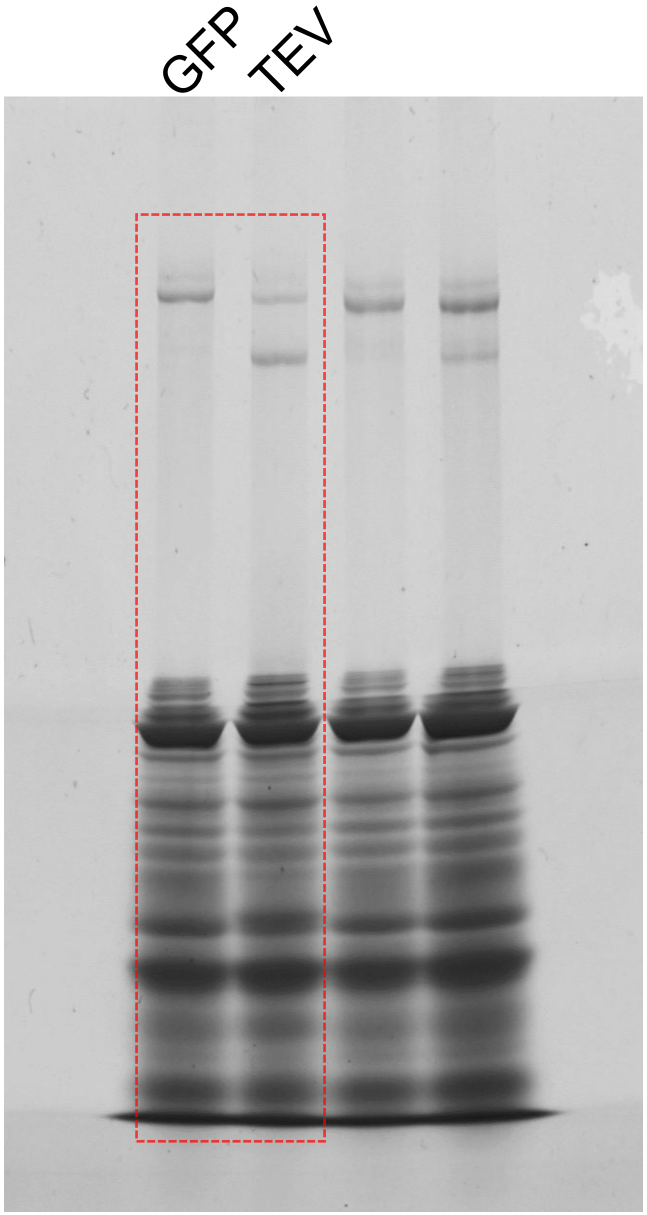

Fig. 1l

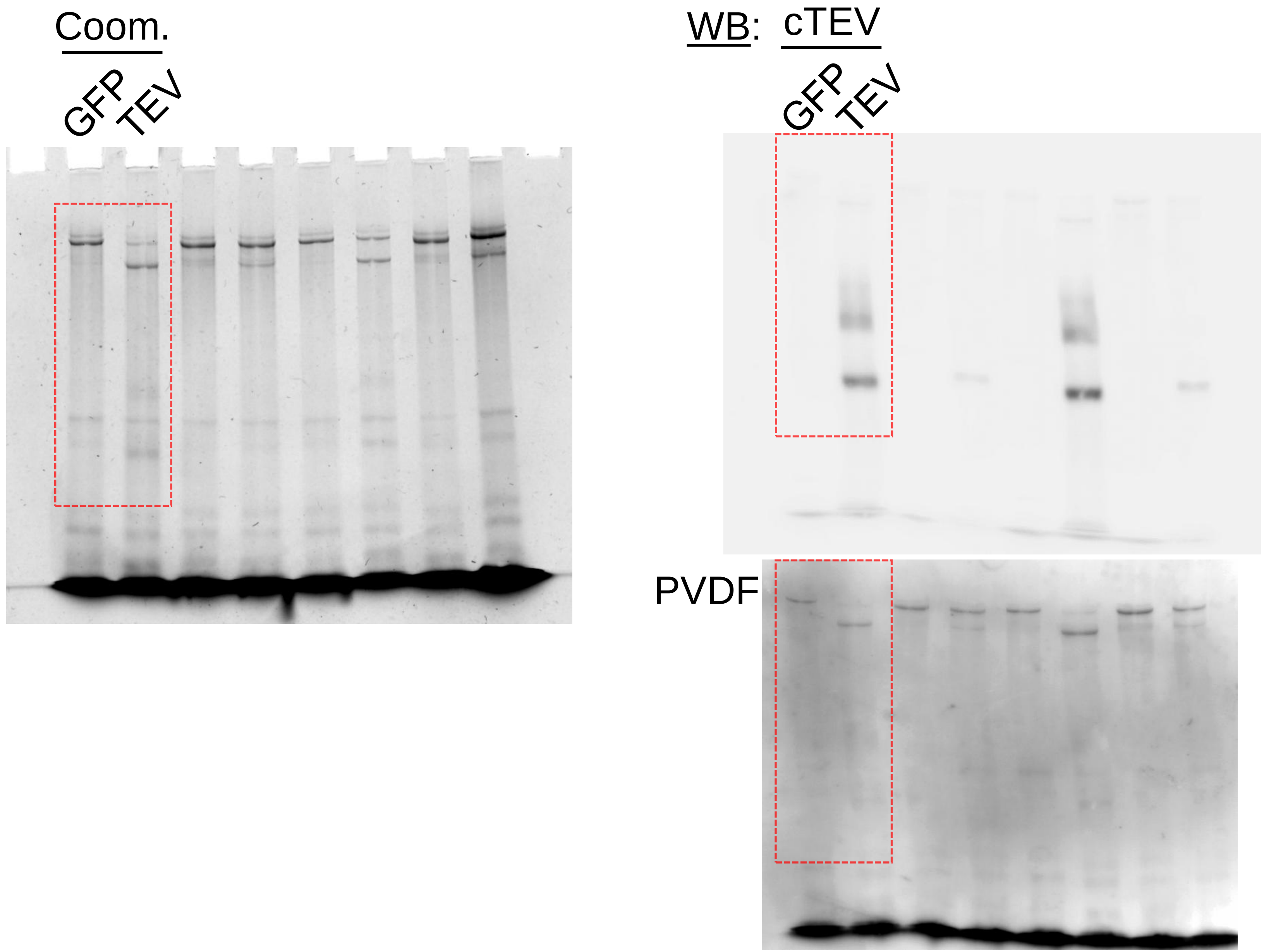

Fig. 3A

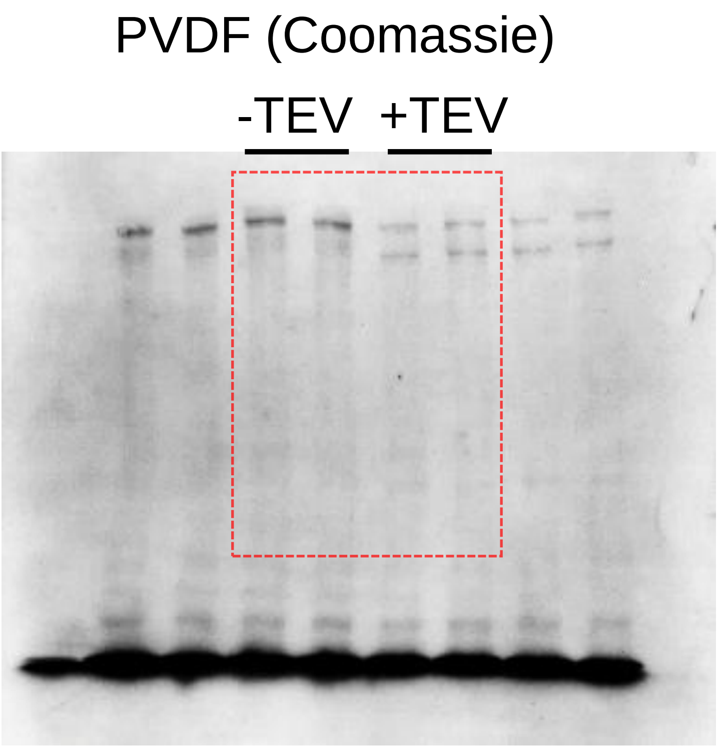

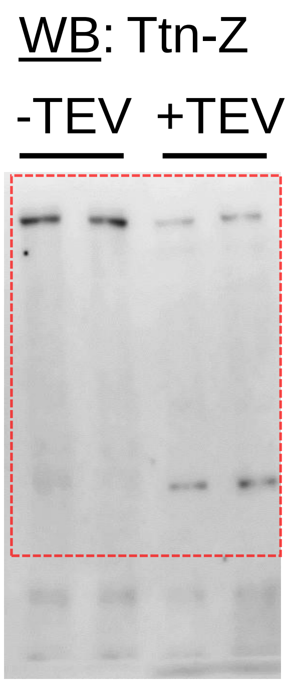

Fig. 5G

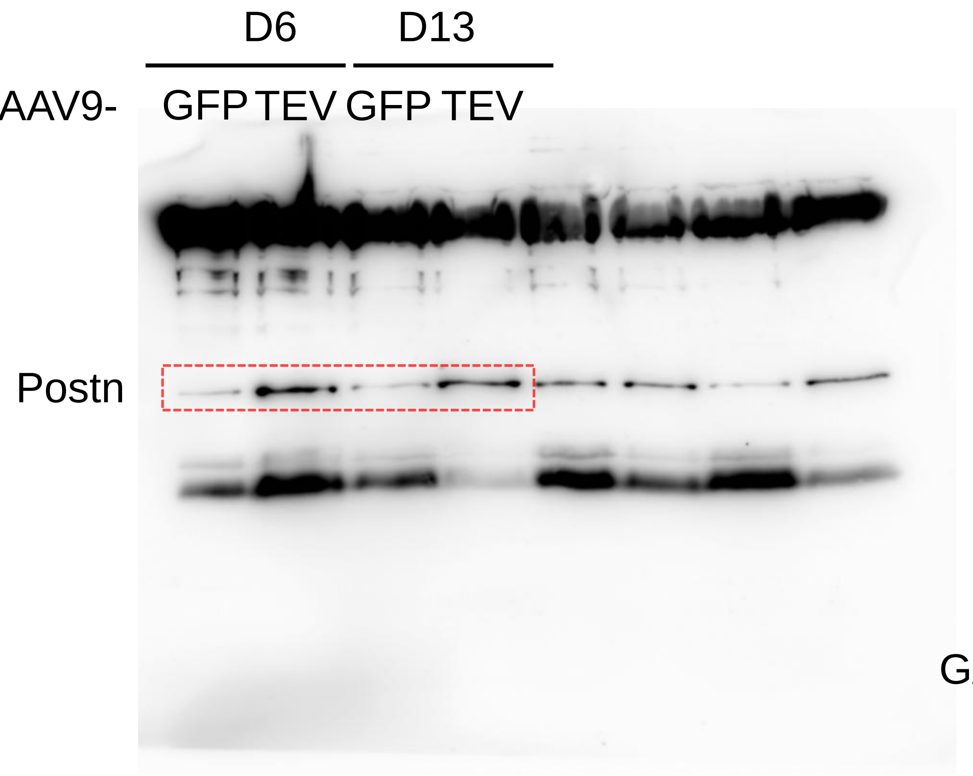
D6
D13
AAV9-
GFP TEV GFP TEV
Postn

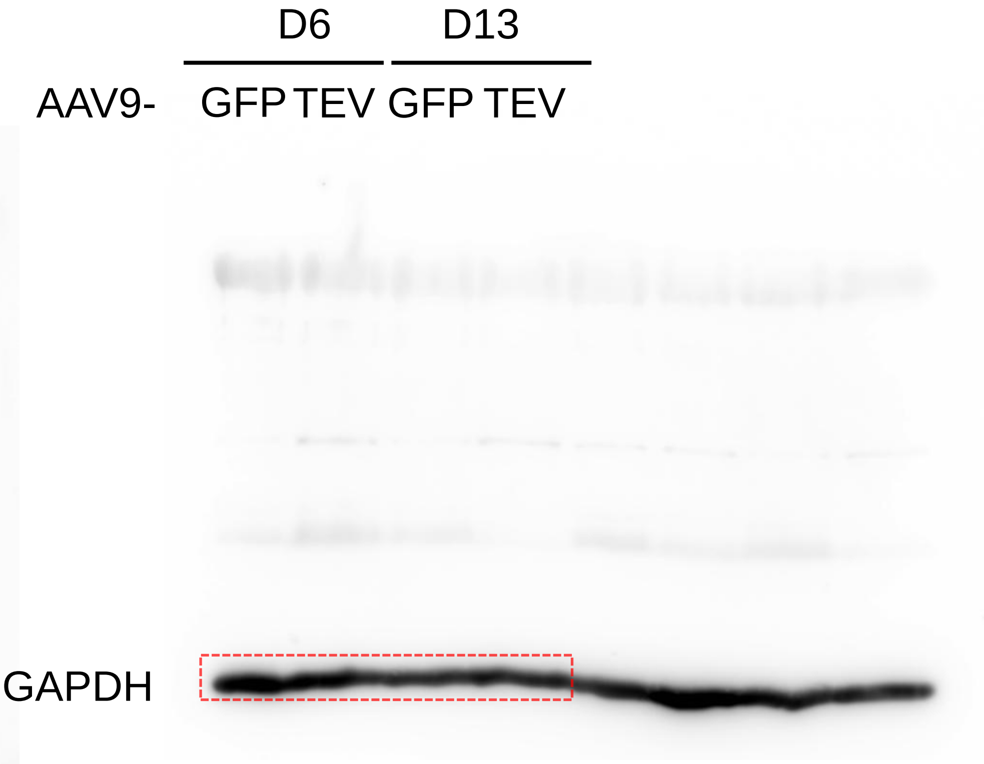
D6
D13
AAV9-
GFP TEV GFP TEV
GAPDH

Fig. 6D

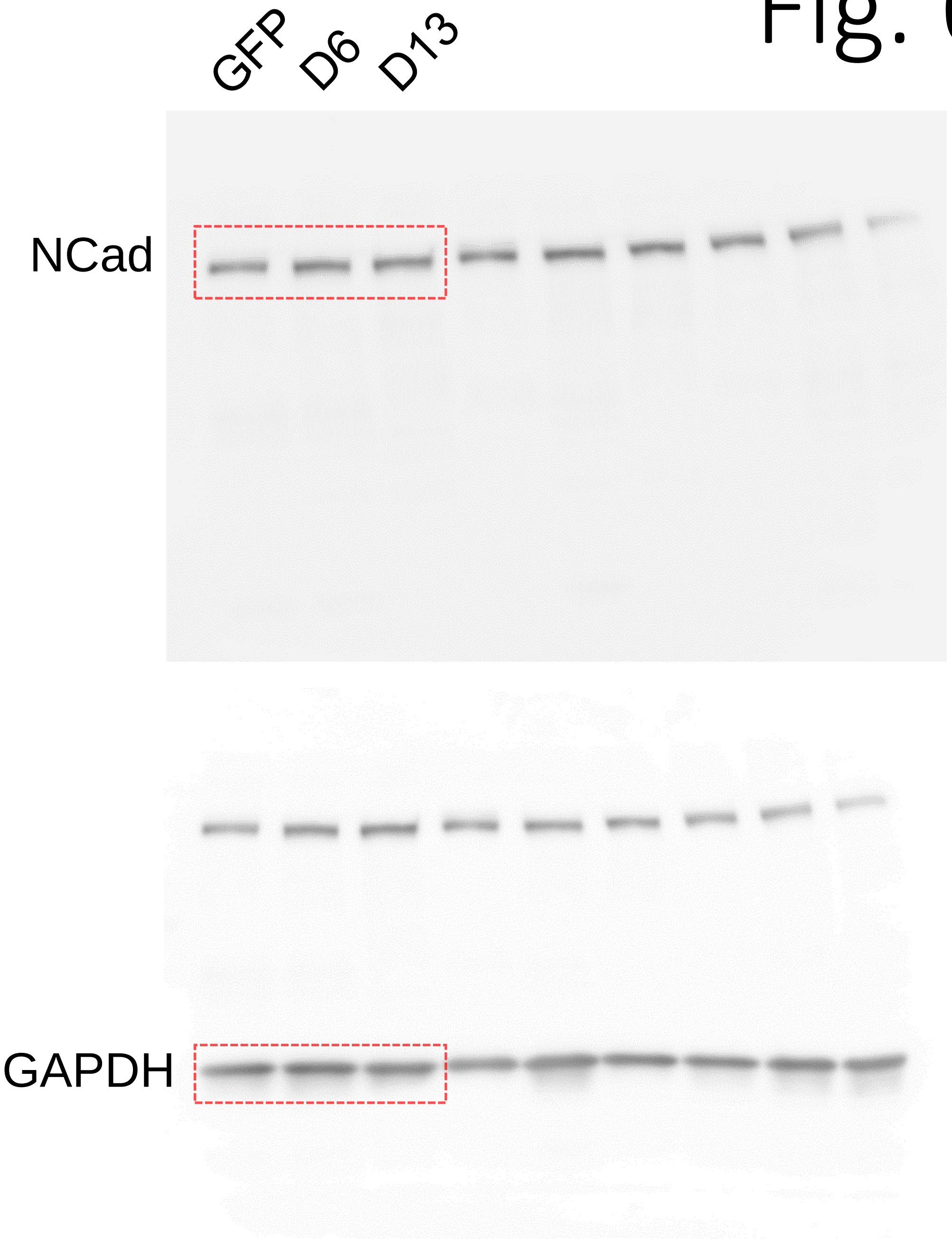

Fig. 6F

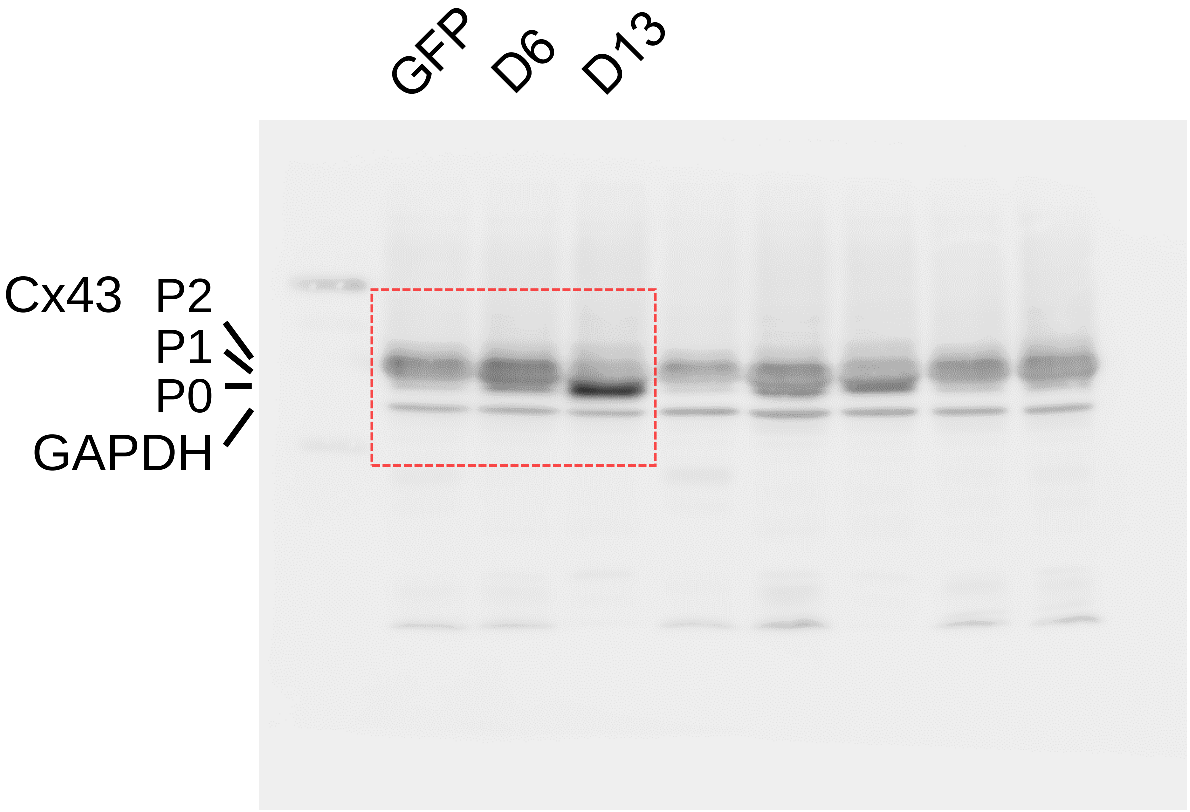

Fig. 6H

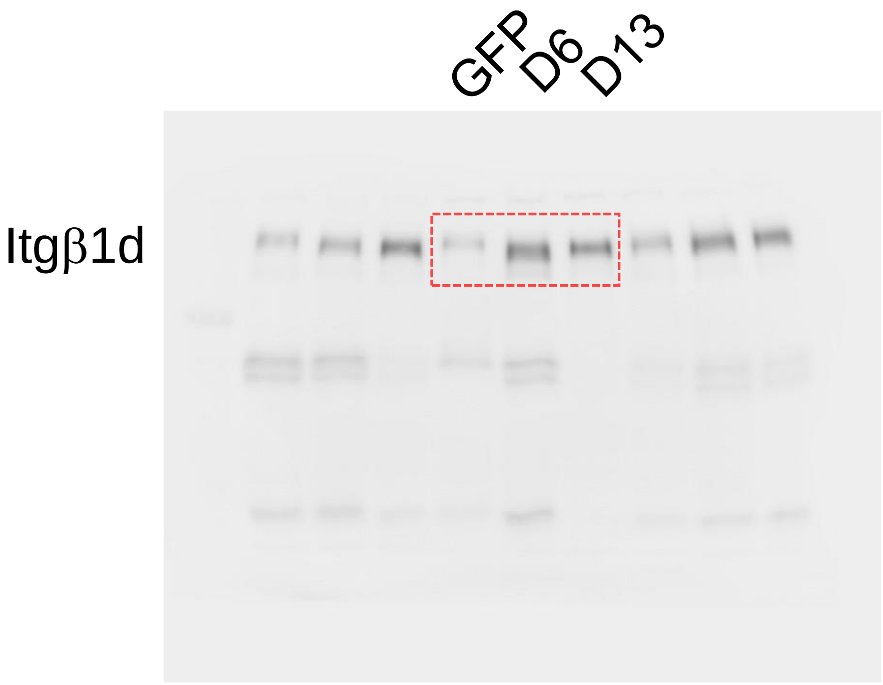

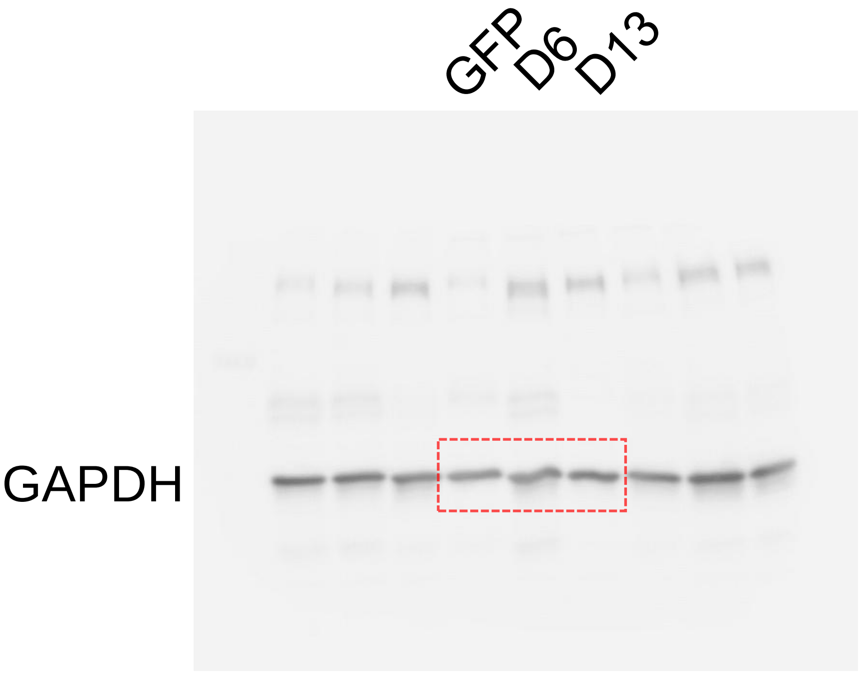

Supplement: Supplementary file 14 — Unprocessed WBs and gels for Figs. 1d,e,f,g,i, 3a, 5g and 6d,f,h. [file 44161_2026_829_MOESM14_ESM.pdf]
