# Supplementary material for: Selective titin cleavage disrupts cardiac mechanical homeostasis to drive heart failure and fibrosis
Source: Nat Cardiovasc Res. 2026 Jun 16;5(6):572–87. doi: 10.1038/s44161-026-00829-z (PMC13271893; doi:10.1038/s44161-026-00829-z)

Fig. S1C Titin-N

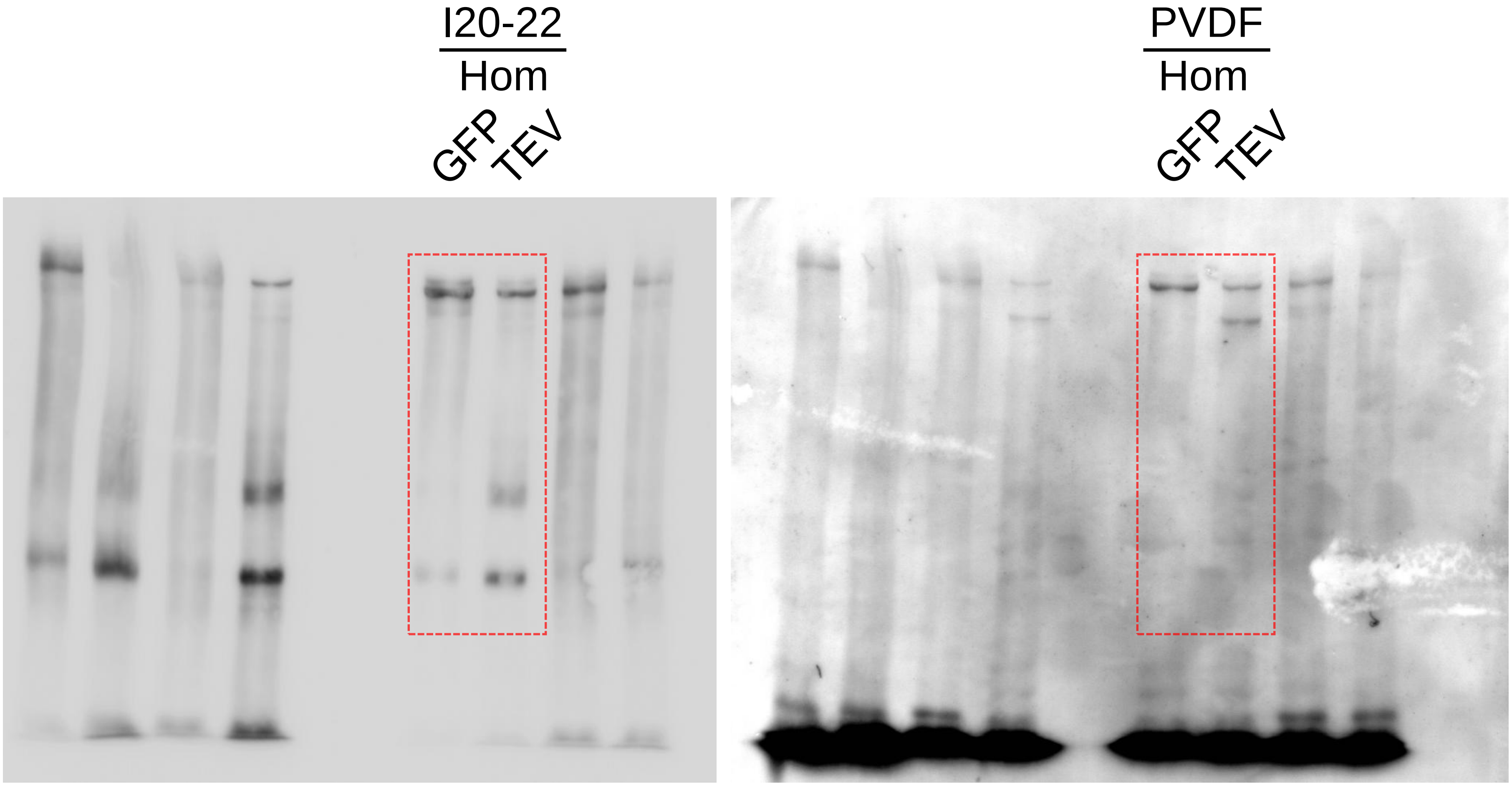

Fig. S1C Titin-N

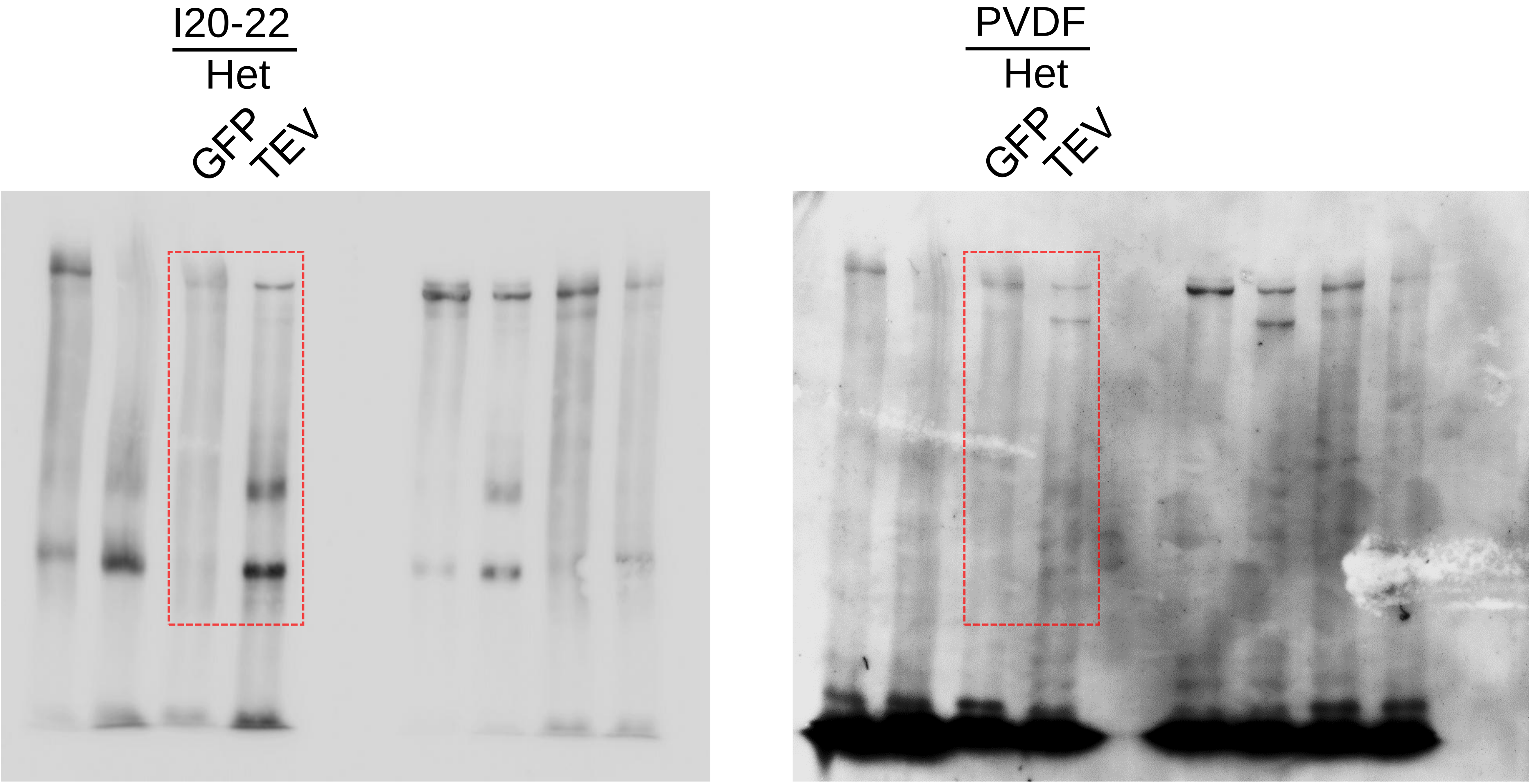

Fig. S1C Titin-C

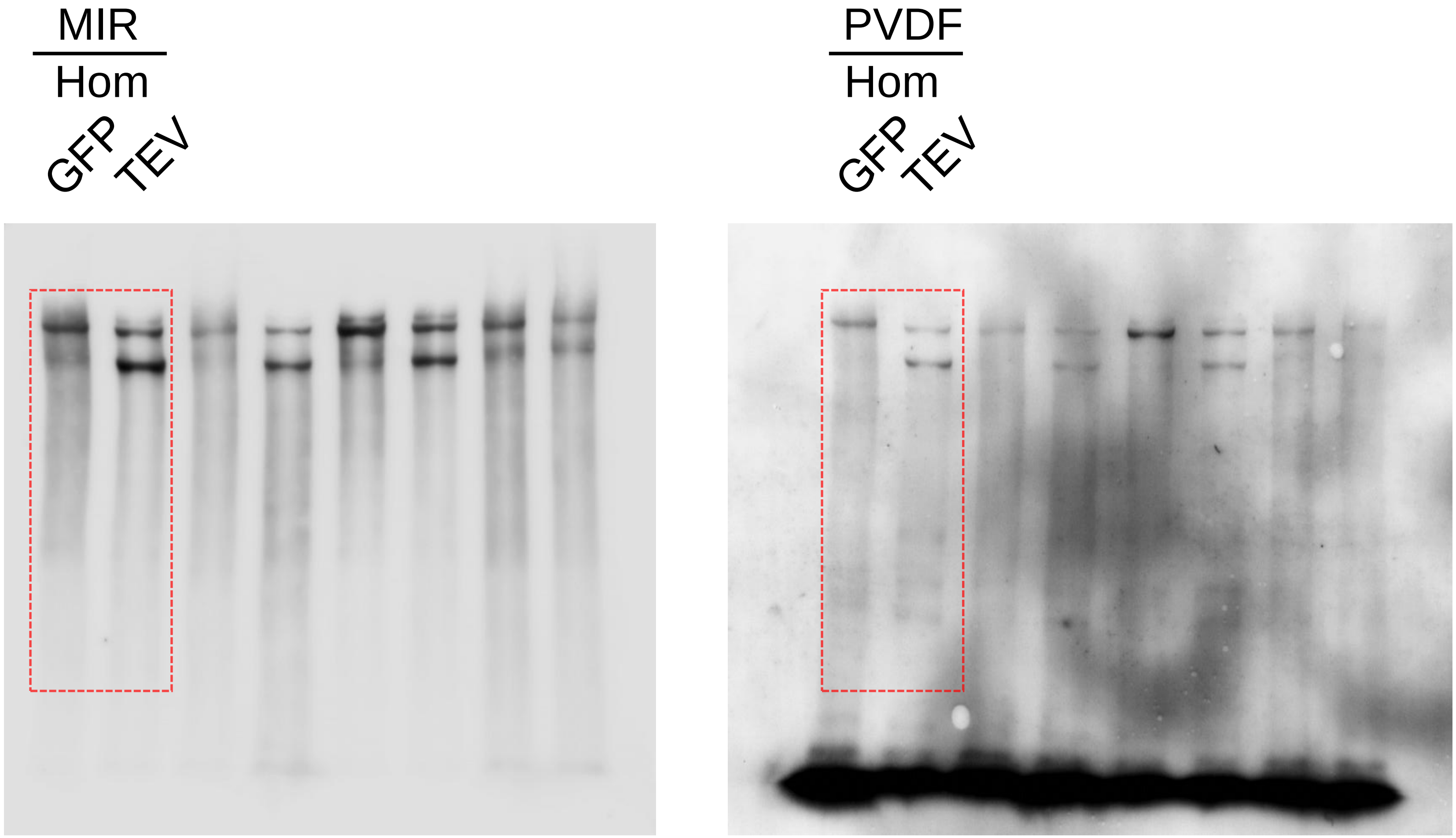

Fig. S1C Titin-C

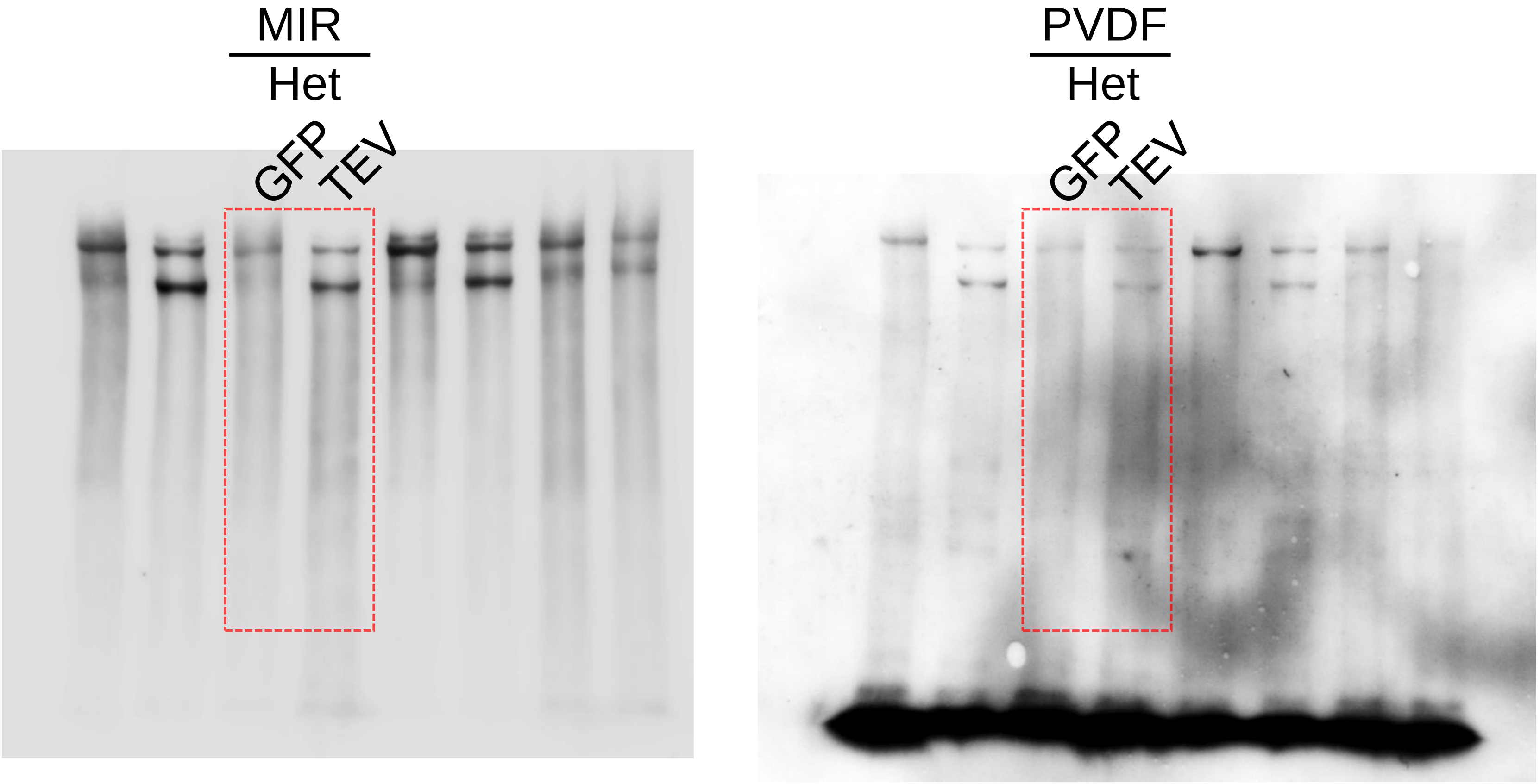

Fig. S1D

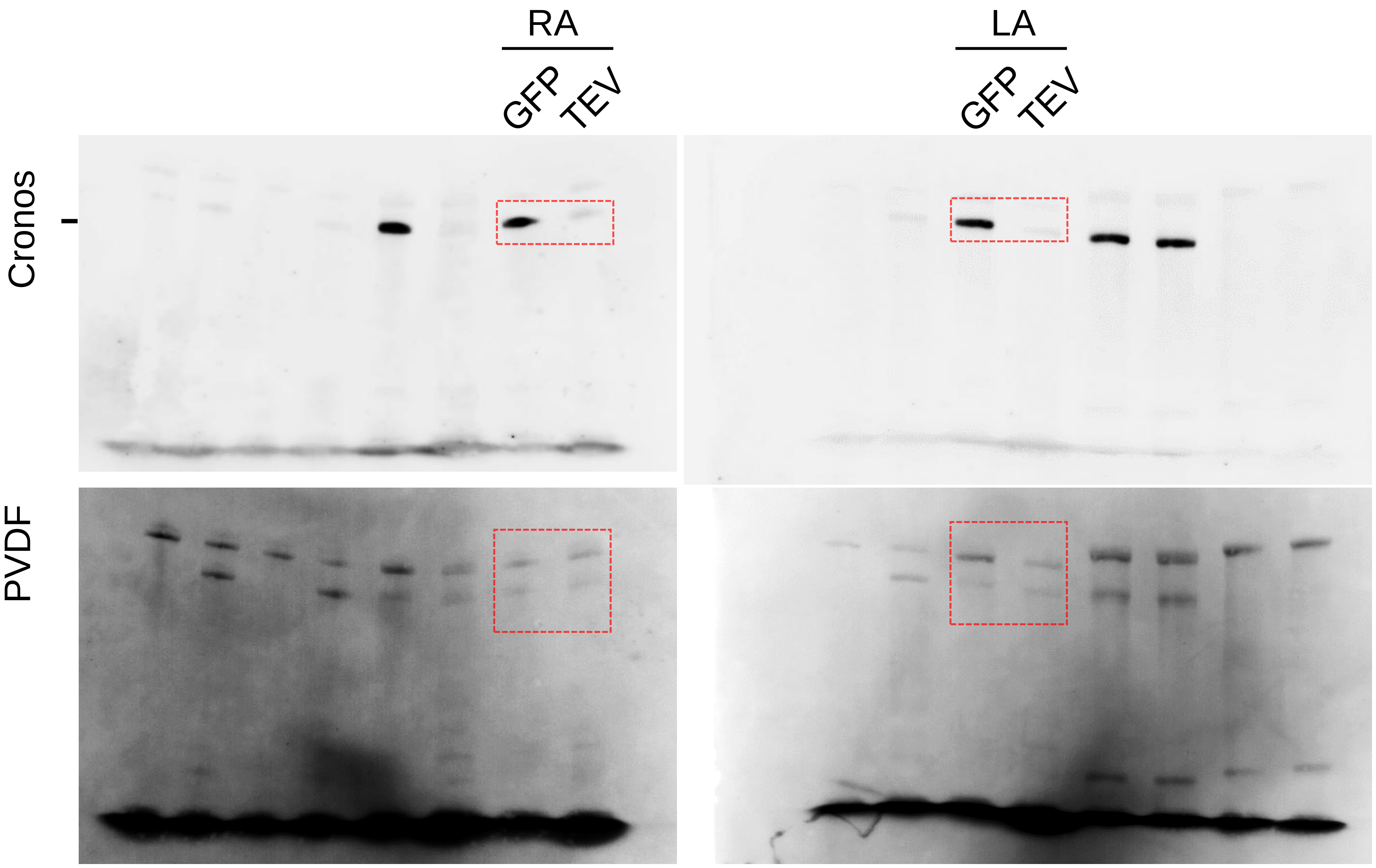

Fig. S1D

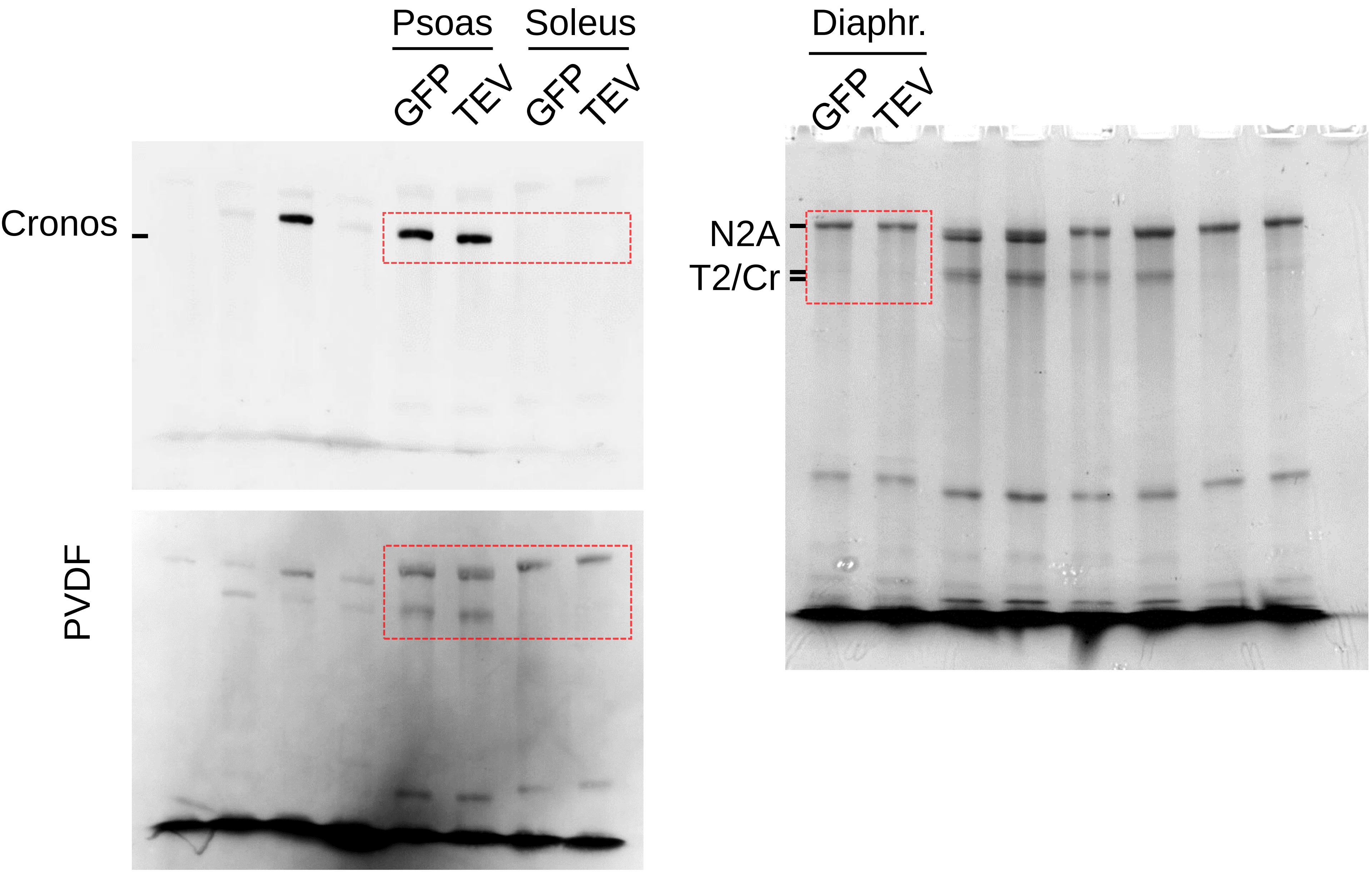

Psoas
Soleus
Diaphr.
GFP
TEV
GFP
TEV
GFP
TEV
Cronos
PVDF
N2A
T2/Cr

Fig. S4A

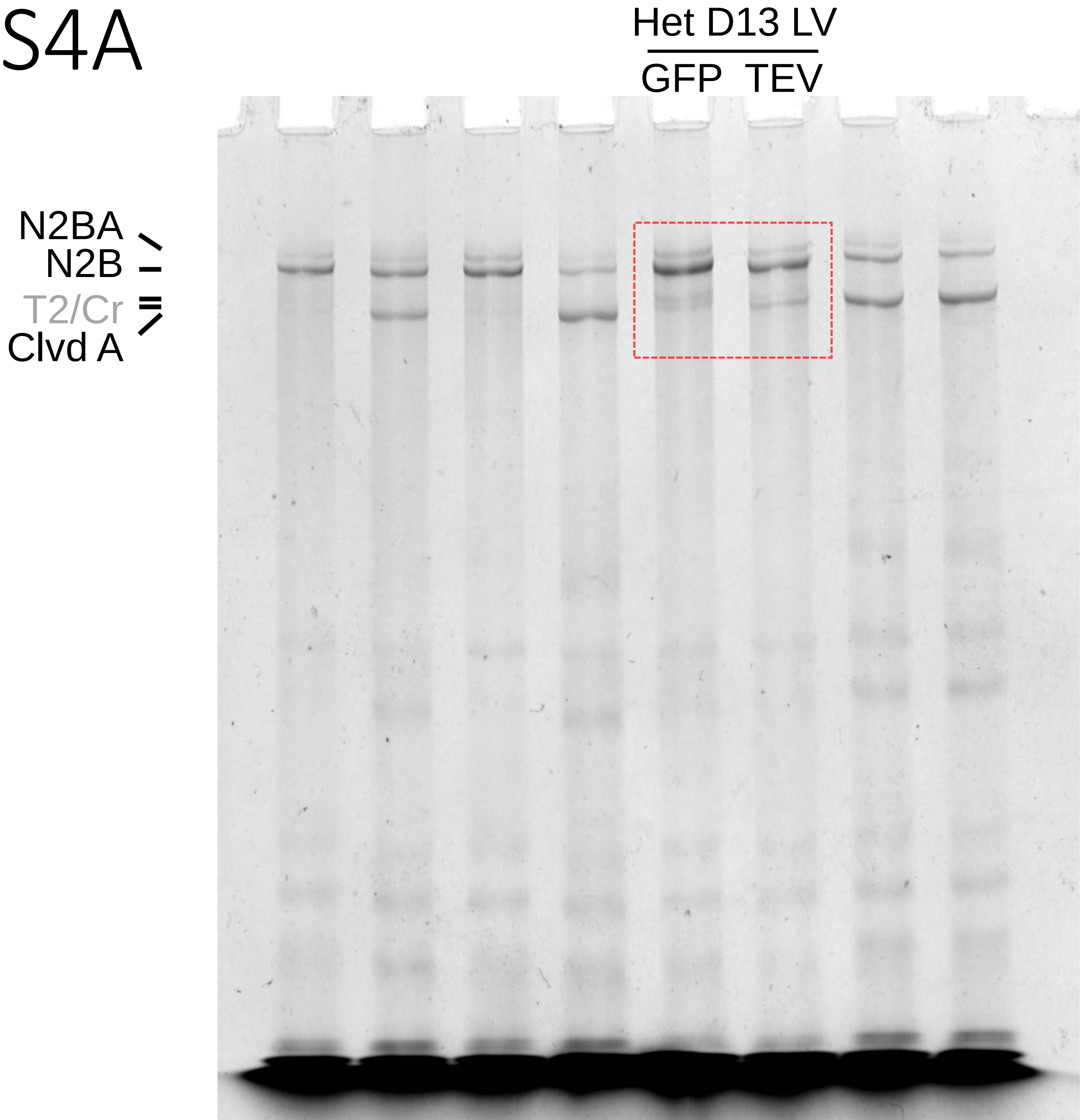

Fig. S4B

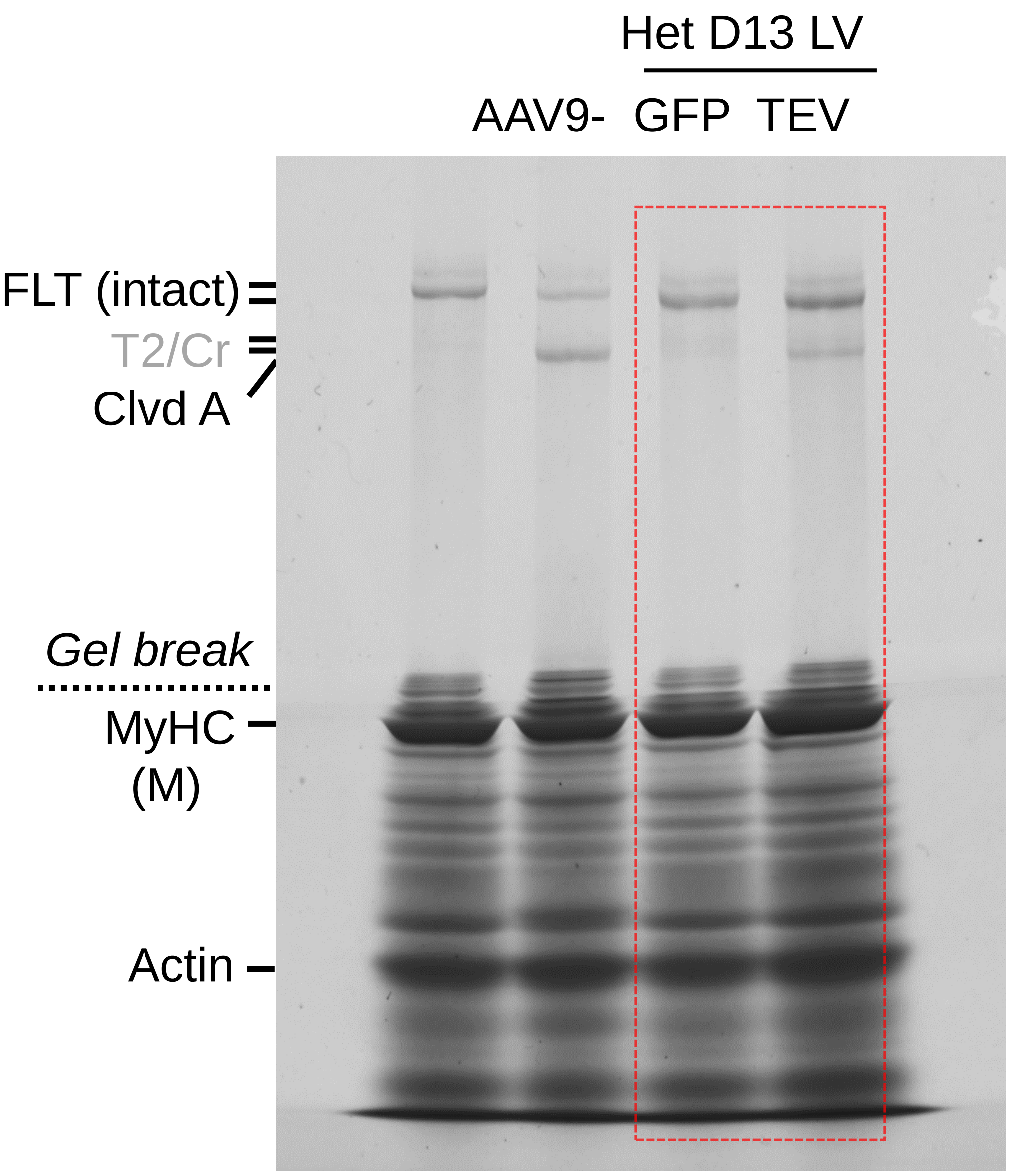
Het D13 LV
AAV9-
GFP
TEV
FLT (intact)
T2/Cr
Clvd A
Gel break
MyHC
(M)
Actin

Fig. S7B

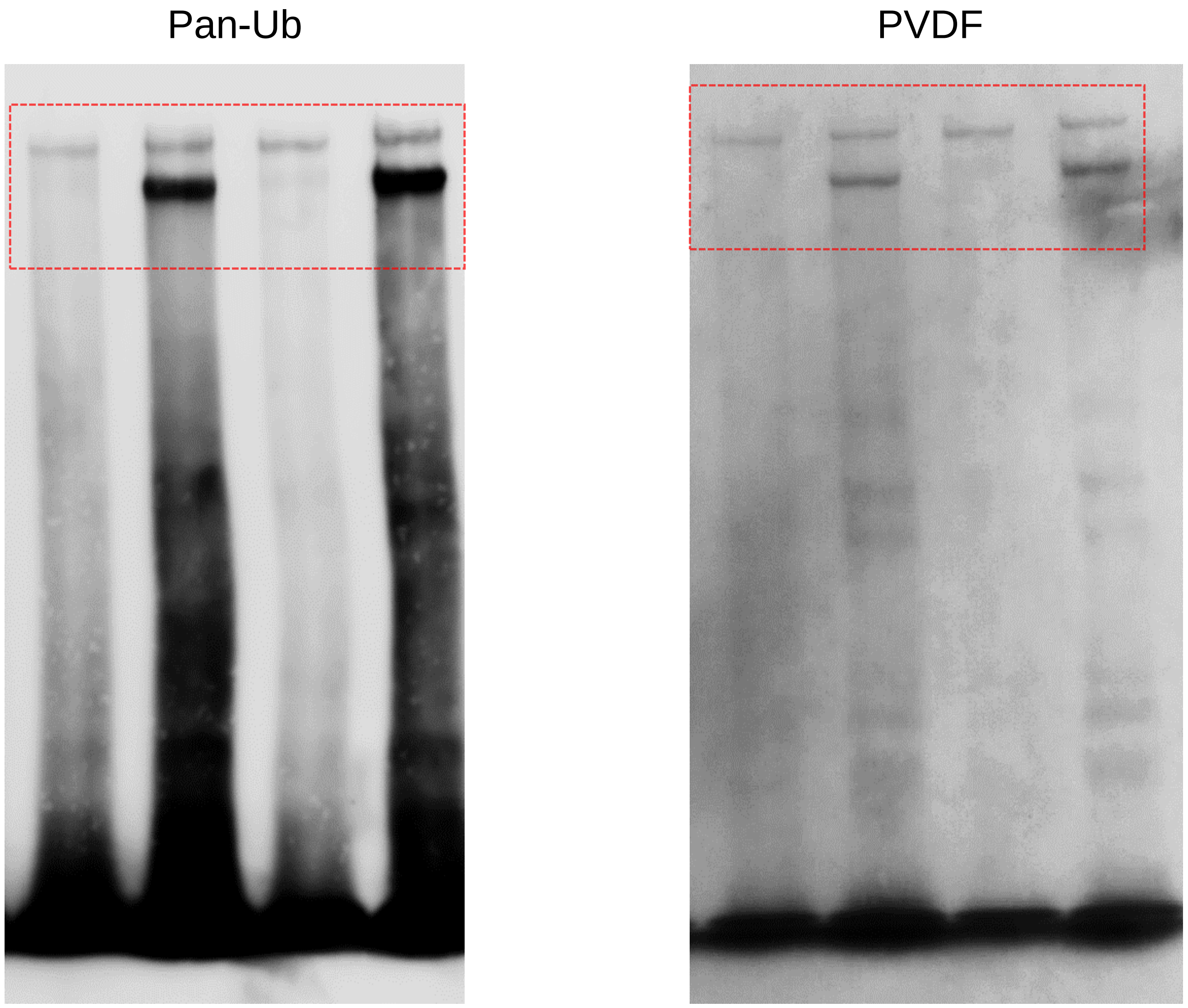

Fig. S7D

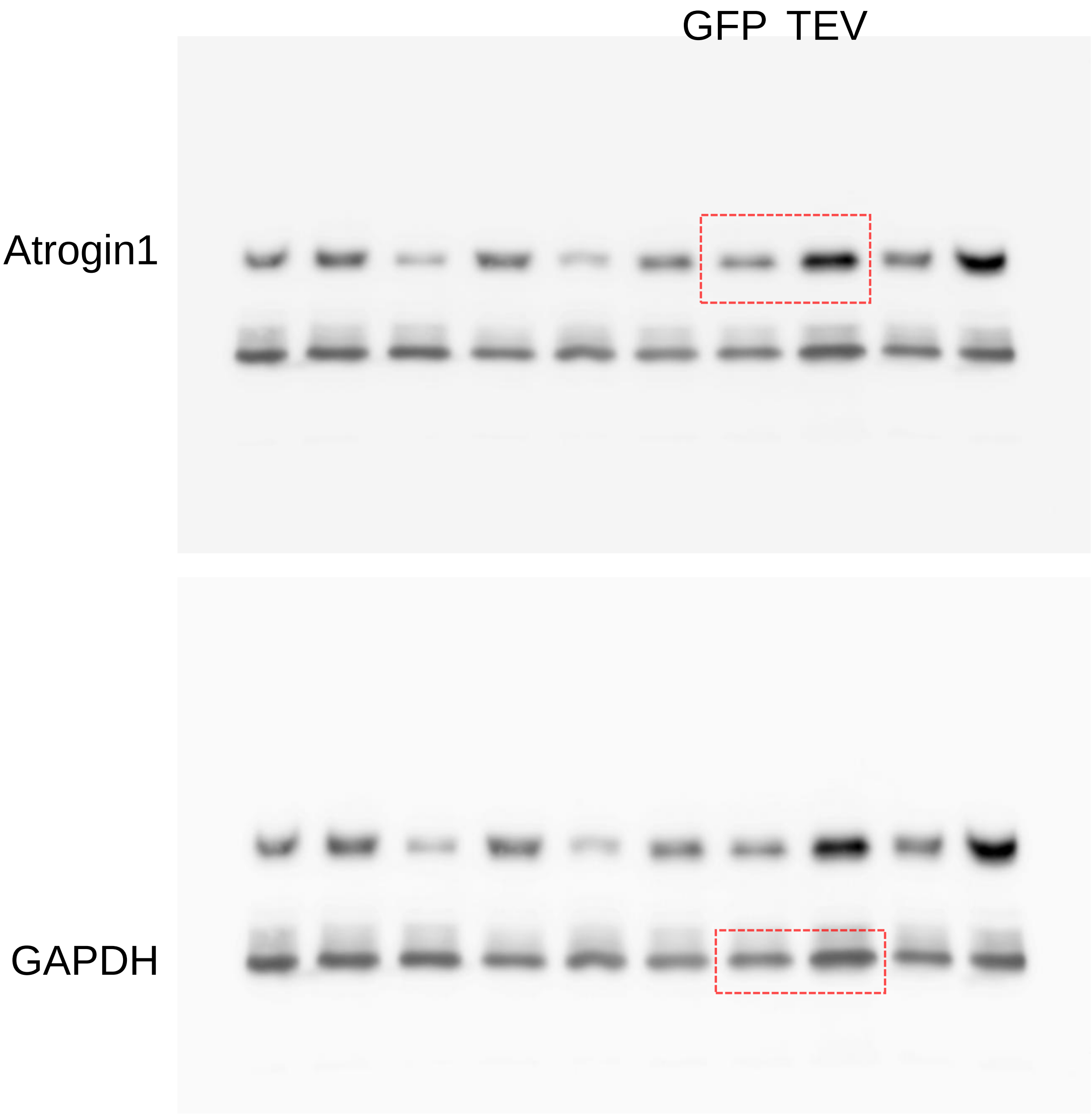

Fig. S7D

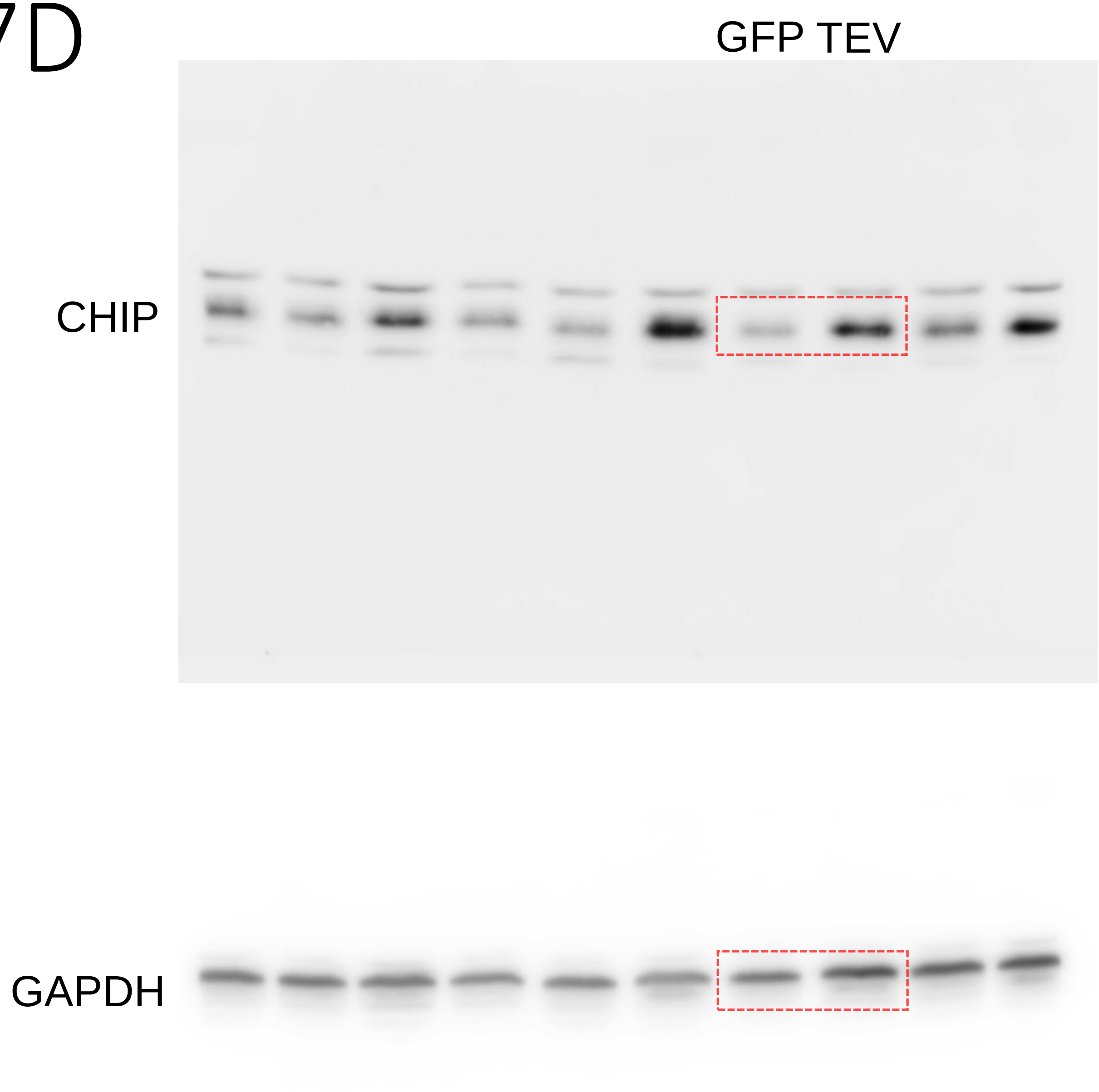

Fig. S7D

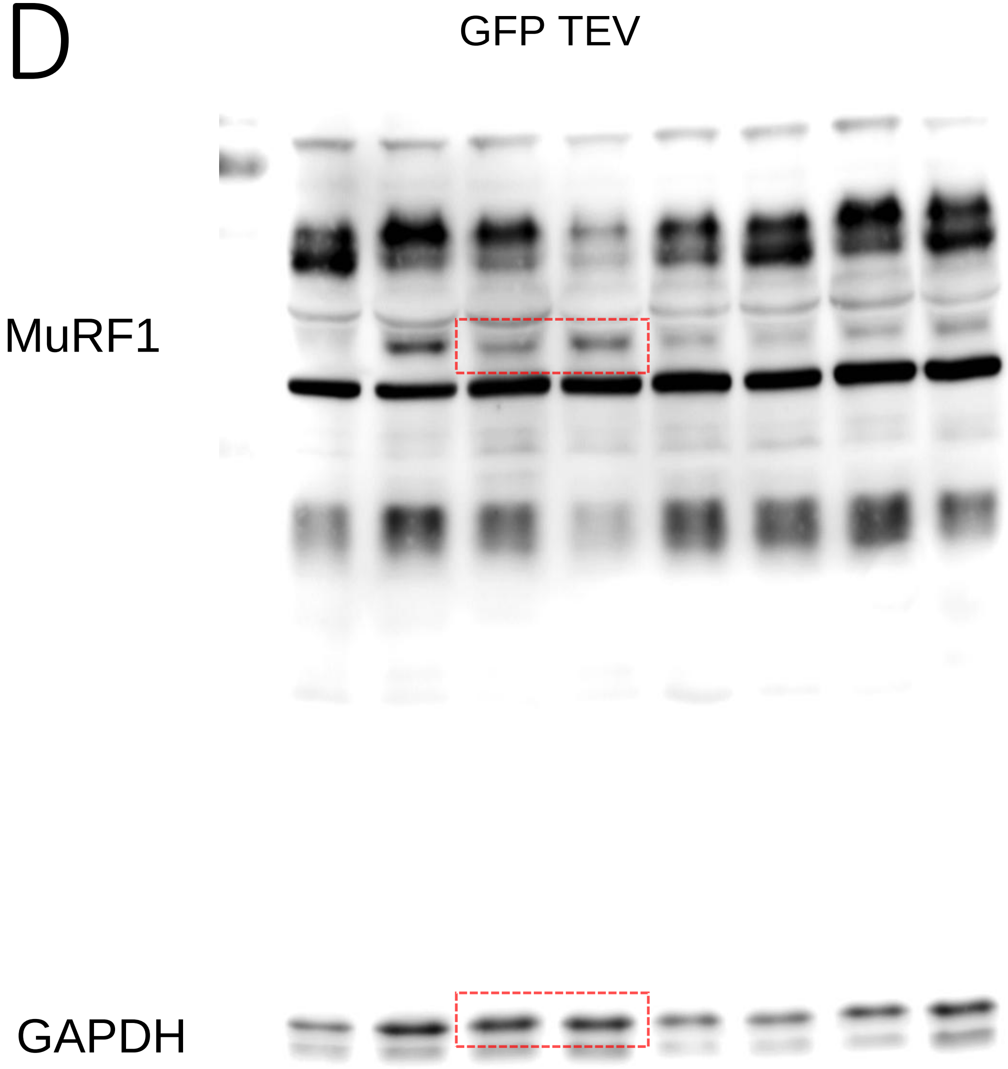

Fig. S7E

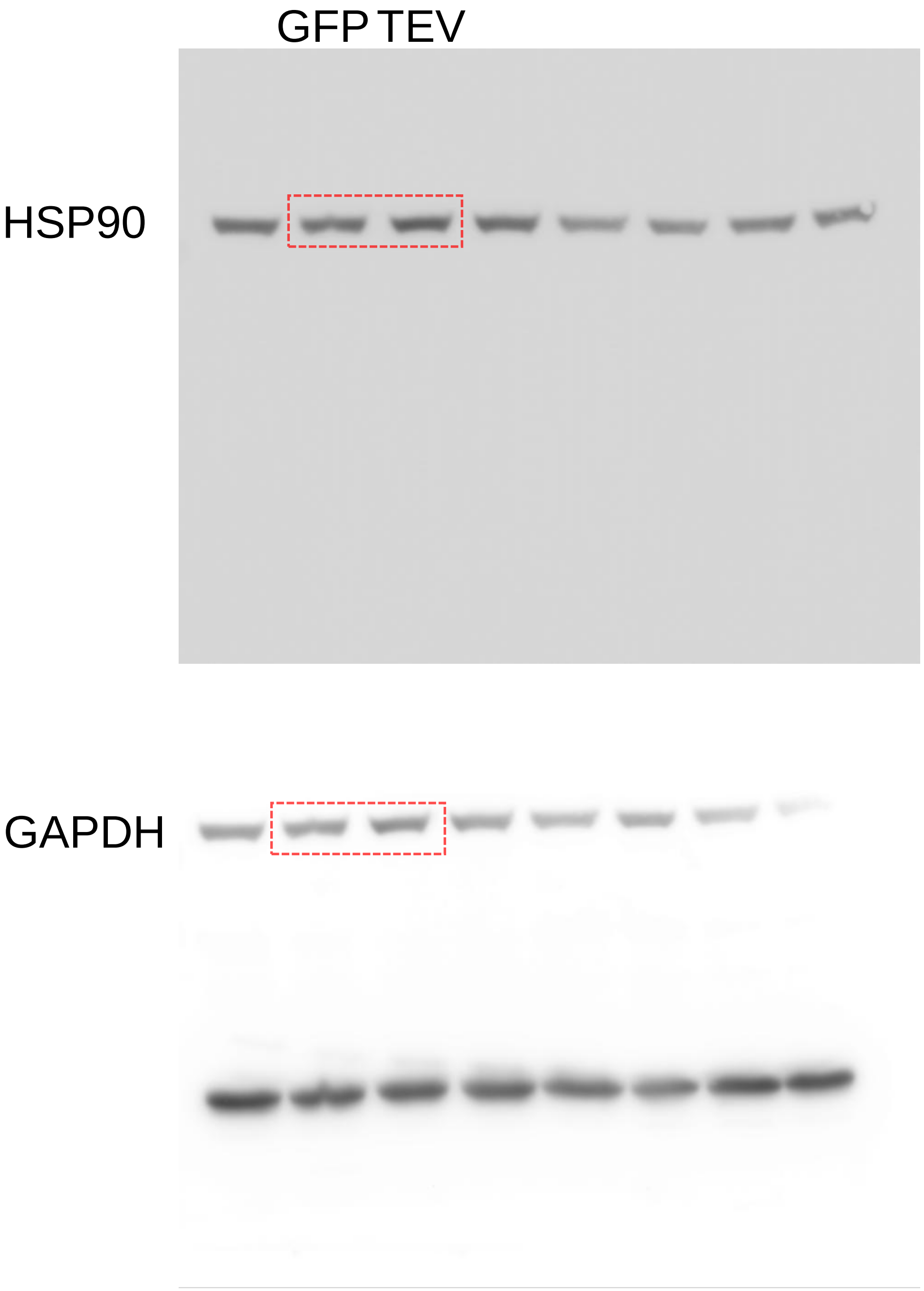

Fig. S7E

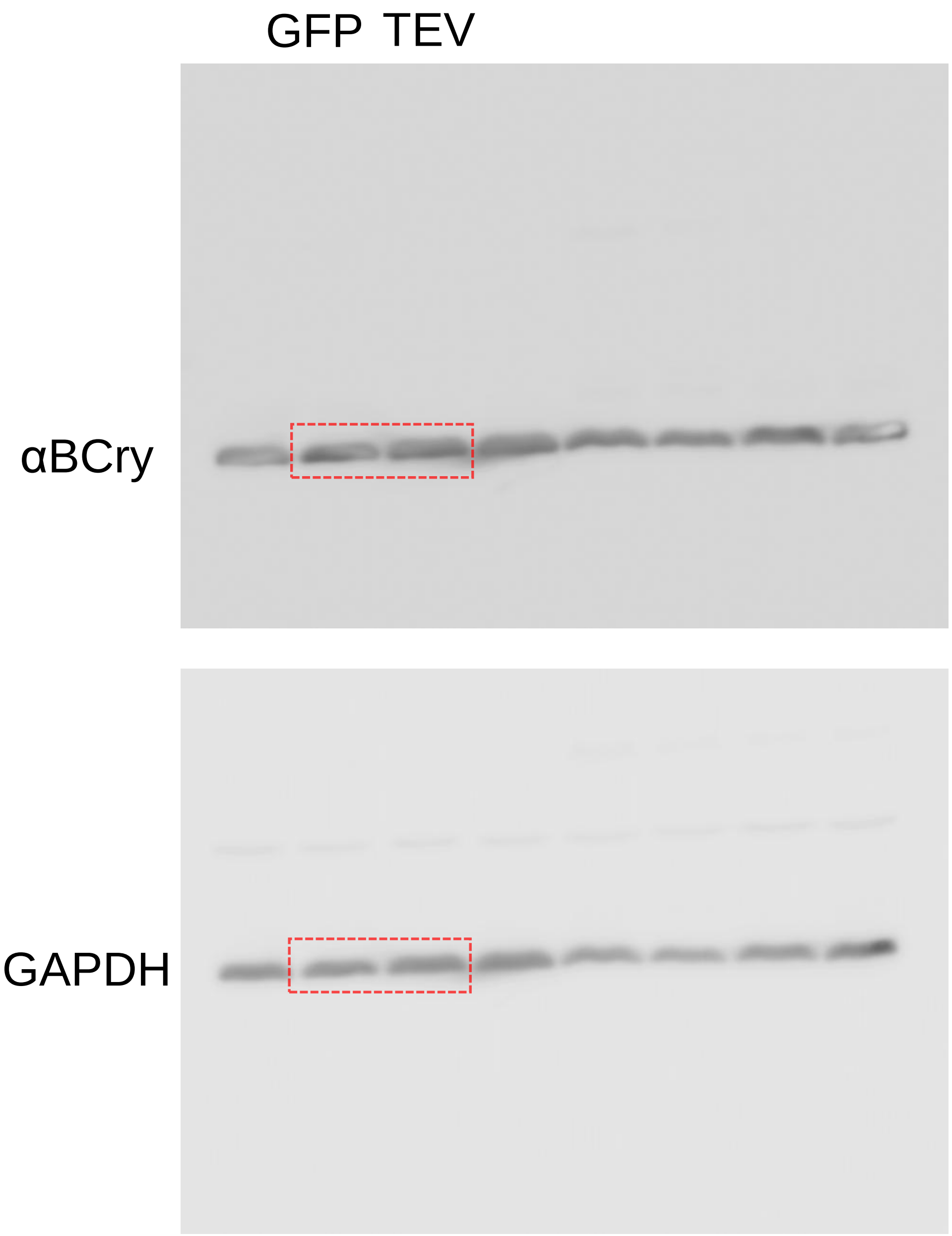

Fig. S7E

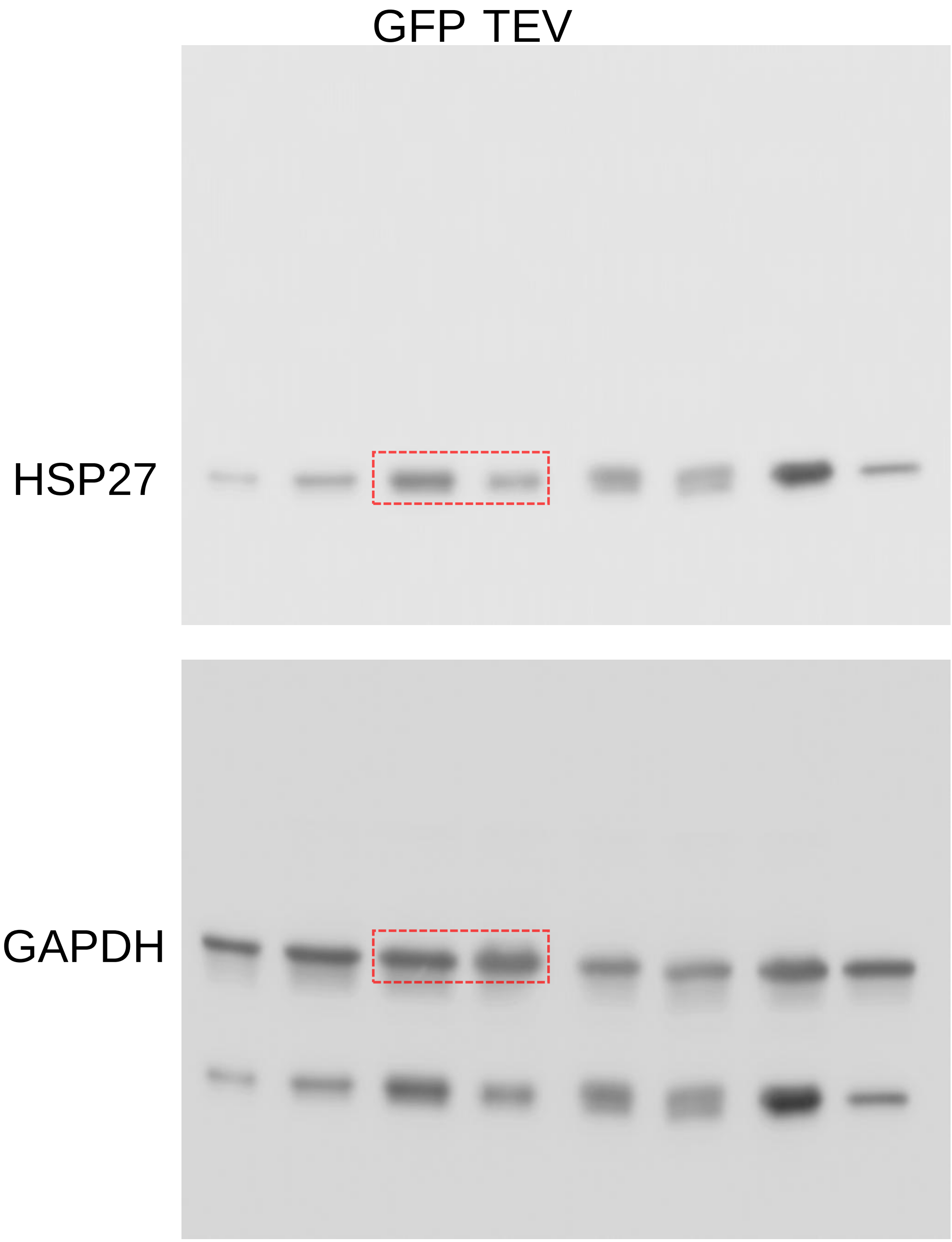

Fig. S7F

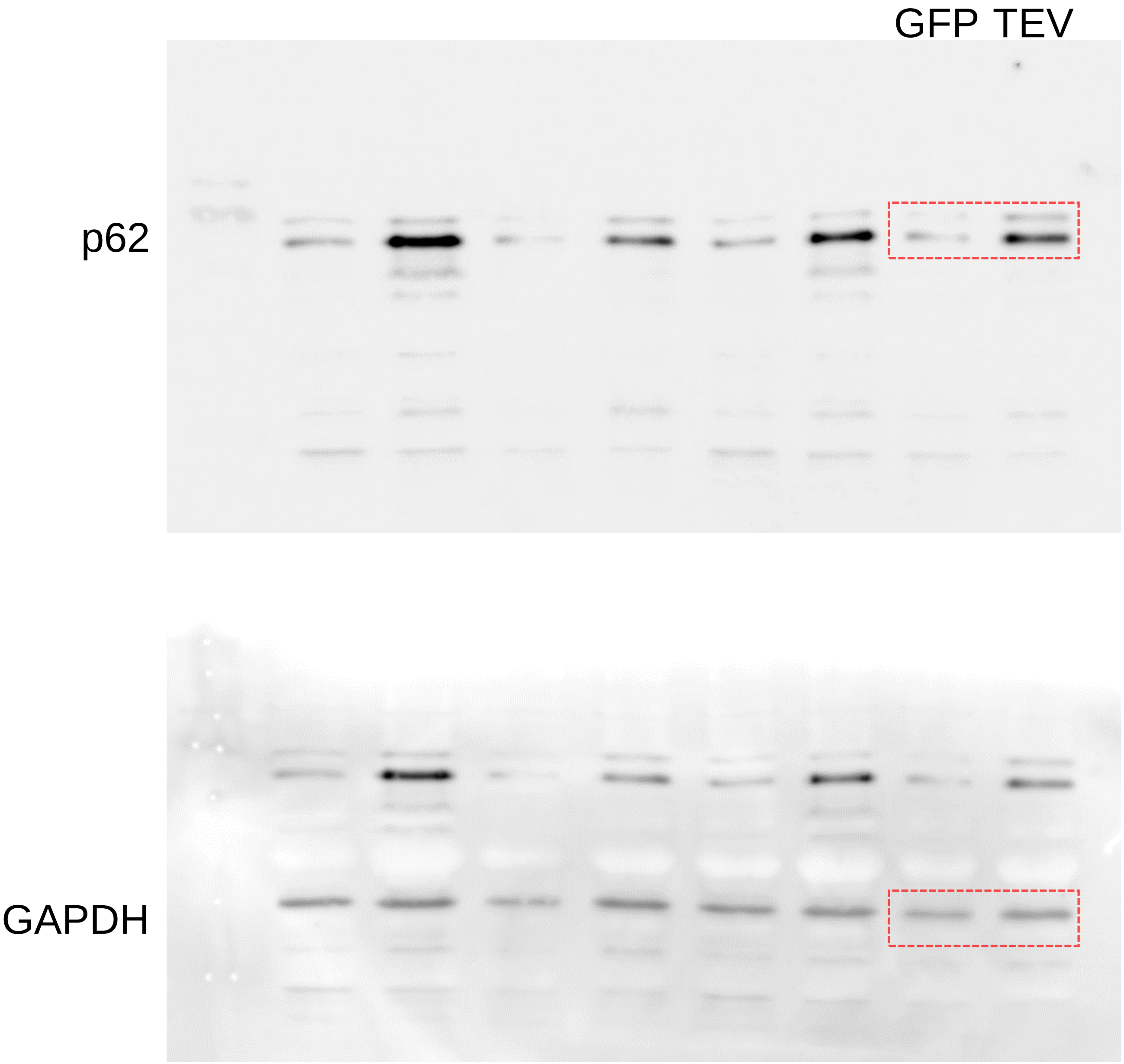

Fig. S7G

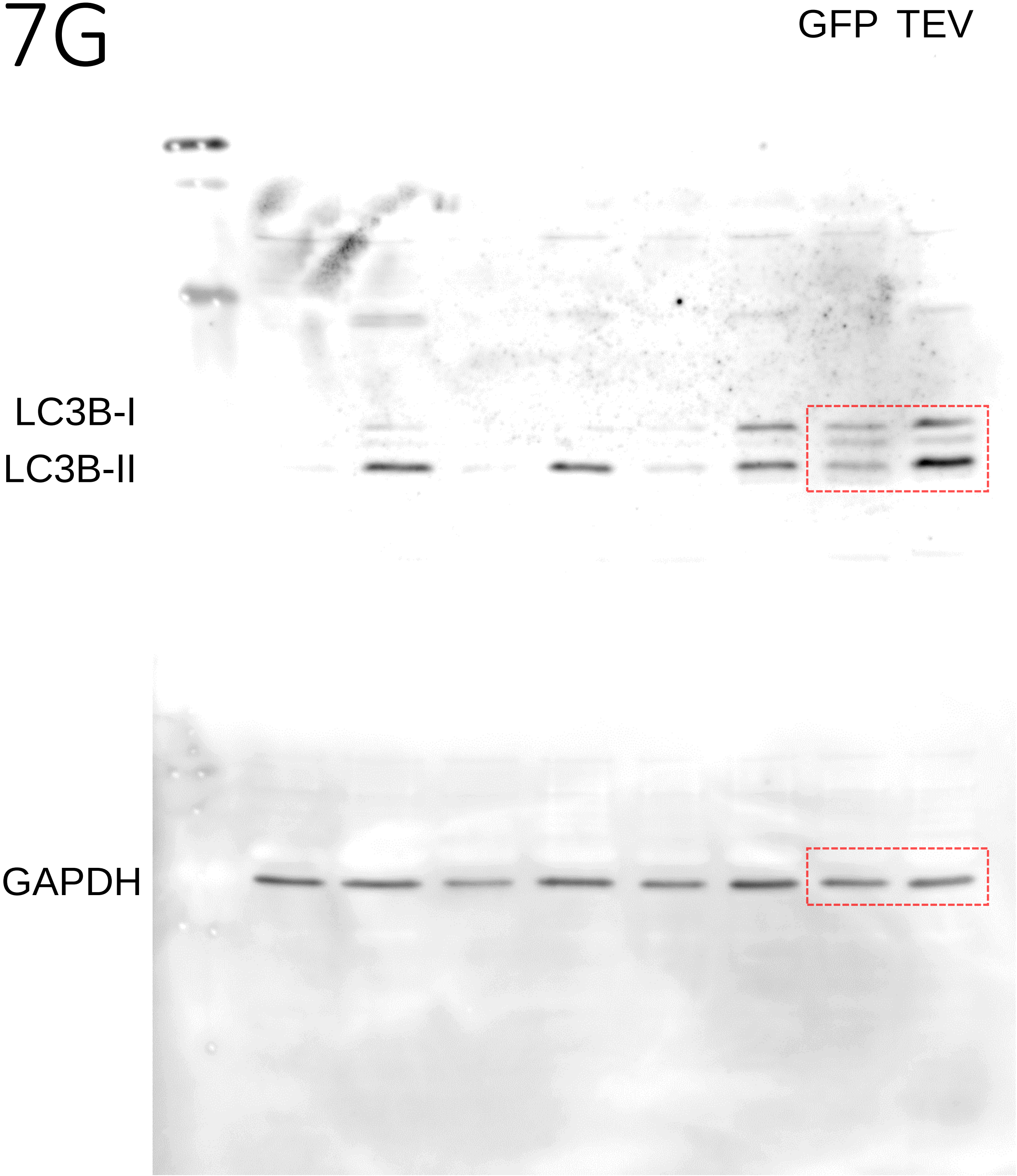

Supplement: Supplementary file 22 — Unprocessed WBs and gels for Extended Data Figs. 1c,d, 4a,b and 7b,d,e,f,g. [file 44161_2026_829_MOESM22_ESM.pdf]
