# Supplementary material for: Selective titin cleavage disrupts cardiac mechanical homeostasis to drive heart failure and fibrosis
Source: Nat Cardiovasc Res. 2026 Jun 16;5(6):572–87. doi: 10.1038/s44161-026-00829-z (PMC13271893; doi:10.1038/s44161-026-00829-z)
Supplement: Supplementary file 2 — Peer Review File [file 44161_2026_829_MOESM2_ESM.pdf]

nature portfolio

Peer Review File

Selective titin cleavage disrupts cardiac mechanical homeostasis to drive heart failure and fibrosis

Corresponding Author: Professor Wolfgang Linke

This file contains all reviewer reports in order by version, followed by all author rebuttals in order by version.

Version 0:

Reviewer comments:

Reviewer #1

(Remarks to the Author)
The study caught my attention, mainly because it connects a defined mechanical disturbance to a recognizable cardiac phenotype. The model is elegant, the dataset extensive, and the topic relevant well beyond basic muscle mechanics.
The story is convincing in parts but slightly overstated in others. If the authors can tighten the data presentation and refine the tone of the discussion, this could be a very solid paper.

Major Comments
1. Clarifying the main sequence of events:
It would help to describe when the key changes occur – titin cleavage, mechanical failure, fibrosis. Even a short schematic or paragraph could show whether the loss of elasticity precedes the structural response.

2. Quantitative clarity
Some central figures (e.g. Fig. 3–5, Suppl. S5–S7) show examples without sufficient numbers. Please include n-values, error bars, and the type of statistical test. Small details like this raise confidence.

3. Fibrosis as consequence, not cause
The link between titin cleavage and fibroblast activation feels mostly correlative. A short note acknowledging that would already help; direct mechanistic evidence would make it even stronger.

4. Intercellular coupling
The intercalated-disk changes are striking. Quantifying connexin-43 or desmosomal markers, if feasible, would support the interpretation. If not, a brief comment on the limitation would be fine.

5. Physiological angle
The predominance of diastolic dysfunction makes good sense – titin mainly affects passive tension. It might help to mention that explicitly and to note that fibrosis likely amplifies stiffness later on.

6. Human or translational link
A short reference to any known titin-cleavage patterns in human cardiomyopathy would strengthen the relevance.

7. Tone of the discussion
A few sentences sound a bit categorical (“titin cleavage causes heart failure”). Softer phrasing would make the argument more persuasive.

Minor Comments:
- Improve axis labeling and add units (e.g. Fig. 2, Fig. 5).
- Define all error bars; specify whether normality was tested.
- Add brief captions to supplementary movies.

Reviewer #2

(Remarks to the Author)
Striated muscles of heart and skeletal muscle systems are reliant on the highly regular, repetitive array of interdigitating, contractile actin and myosin filaments into the smallest contractile units of striated muscle, the sarcomeres. The largest protein on the vertebrate body, titin (encoded by the TTN gene) provides, amongst many assembly functions, a mechanical, elastic link between the actin and myosin filaments and thus assures the central position of myosin filaments at the onset of each contraction, the equal dissipation of imbalanced forces, and thus the mechanical integrity of the entire system. Loss of mechanical continuity of titin between the actin and myosin filaments in the I-band is therefore particularly destructive for striated muscle sarcomeres.
Previous work by other groups showed that cardiac stressors like ischemia/reperfusion-induced injury, inflammation and others lead to the activation of proteases that cleave several intracellular cytoskeletal proteins including titin, thus impairing the essential mechanical connectivity of contractile structures and the cytoskeleton. The results are serious contractile dysfunction and adverse cardiac remodelling, including the proliferation of fibroblasts and excessive, albeit possibly compensatory collagen deposition.
Freundt and co-workers have reduced the complexity of cardiomyocyte stress-induced proteolysis to a single target by genetically engineering mice to include the highly specific tobacco etch virus (TEV) restriction protease site into the I-band portion of titin. Virus-mediated expression of TEV protease under a highly cardiac-specific promoter then triggers relatively rapid and complete cleavage of I-band titin in a significant fraction of cardiomyocytes. This experimental design should therefore allow attributing any cellular and physiological changes solely to the cleavage of titin and not any other cytoplasmic or extracellular protease. This complex and comprehensive study is of very high technical quality, well-written and well-illustrated.
Remarkably, the authors observe that I-band titin cleavage of only a relatively small proportion of cardiomyocytes alone suffices to trigger rapid cardiac failure, fibrotic remodelling of the heart, and pathological remodelling of cardiomyocyte cell-cell contacts at the intercalated disks. These important findings demonstrate beyond reasonable doubt that titin is directly involved in cellular communication that converts information on mechanical stress into signals communicating with the extracellular compartments and that its cleavage alone suffices to trigger a rapid fibrotic response of the cardiac fibroblast population.
These are important and novel findings that will be of interest to the readers of Nature Cardiovascular Research.

There are some important questions about the experimental design, the interpretation and generalisation of the results, however.

1. There is some possible confusion about the numbering of titin domains. According to the primary reference by Rivas-Pardo, the TEV site was introduced between titin I-band domains I86 and I87, not immunoglobulin domains 86-87 as stated here. Please use the titin domain numbering referenced to the IC sequence, which is less ambiguous (due to alternative splicing) than I-band domain number, see e.g. [1] and online titin data base resources. In the complete titin domain array, the two domains are Ig-100 and Ig-101 in the distal I-band.
2. Why was the TEV recognition site introduced into titin's I-band region between immunoglobulin-like domains 100 and 101? Does this mirror any known cleavage site, (if yes, please reference) or is the genetic mouse model used, as the primary publication by Rivas-Pardo 2020 suggests, designed for a different and purely fundamental question? Are there any data on physiological cleavage between or near domains I-86 and 87? If the model does not mirror a mapped disease-linked cleavage site, what is its disease relevance? Where is the closest mapped proteolytic titin cleavage site, and would this influence the interpretation of the data? As I-band titin is hypothesised to interact with actin filaments under certain conditions as [2-4], the position of physiological or non-physiological cleavage would likely matter for the resulting mechanobiological defects. Metalloprotease cleavage as identified by [5] is N-terminal to the TEV site used here. It would be helpful to discuss these points.
3. The cleaved-I is assigned to two corresponding to N2BA and N2B: how were these unambiguously identified beyond sizes on a gel? Fragment-specific antibodies or mass-spectroscopy data would be supportive of the assignment. Please make this clearer.
4. The AAV9-mediated TEV expression under a cardiac troponin-T promoter is assumed to be completely heart specific, however, the pleiotropism of AAV9 is a significant issue for gene therapy approaches [6]. Please comment whether any off-site expression (Liver?? Skeletal muscle?) could contribute to the observed phenotypes and how this was controlled and ruled out.
5. The authors laudably resist the temptation of discussing the relevance of their experimental findings for inherited cardiomyopathies. Proteolytic cleavage of I-band titin would reflect homozygous truncating variants in this region. Albeit biallelic truncating variants in titin can cause severe early-onset myopathies with cardiomyopathy, at least one truncation is always in the A-band or M-band, thus maintaining mechanical connectivity between thick and thin filaments [7-10]. Consequently, in human muscle with biallelic truncating variants studied, titin remained A-band bound and does not recoil to the Z-disk. Dilated cardiomyopathy due to truncating titin mutations is heterozygous, i.e. one allele is normal, while the majority of mutations cluster in the A-band region, therefore providing mechanical continuity because they are retained (albeit likely weakened), as in early onset myopathies. No human disease-linked I-band homozygous or compound-heterozygous variants have been reported, possibly because of intrauterine lethality, as would be inferred from the complete loss of sarcomere integrity after I-band cleavage. Although genetic truncation, manifest over long time and possible mitigated by differential splicing, is not directly comparable to acute proteolytic cleavage, a short discussion with reference to genetic disorders and spontaneous abortions e.g. [11] would be helpful.

Minor points

It is remarkable that the proteomic analysis shows several upregulated ubiquitin ligases (TRIM33, TRIM35, TRIM47). None are "classical" sarcomere-associated tripartite motif family ligases, please comment on their possible implication in sarcomere instability. TRIM35 appears highlighted three times in Figure S5e. Please check if this is a mistake.

1. Chauveau, C., J. Rowell, and A. Ferreiro, A Rising Titan: TTN Review and Mutation Update. Hum Mutat, 2014. 35(9): p. 1046-59.
2. Squarci, C., et al., Titin activates myosin filaments in skeletal muscle by switching from an extensible spring to a mechanical rectifier. Proc Natl Acad Sci U S A, 2023. 120(9): p. e2219346120.
3. Herzog, W., The multiple roles of titin in muscle contraction and force production. Biophys Rev, 2018. 10(4): p. 1187-1199.
4. Wagner, F.A., et al., Direction-dependent contributions of cardiac myofilament networks to myocardial passive stiffness reveal a major disparity for titin. Basic Res Cardiol, 2025. 120(4): p. 761-777.
5. Ali, M.A., et al., Titin is a target of matrix metalloproteinase-2: implications in myocardial ischemia/reperfusion injury. Circulation, 2010. 122(20): p. 2039-47.
6. Firnberg, E., et al., Directed evolution of liver-detargeted AAV vectors for systemic gene delivery to skeletal muscle and heart. Mol Ther Methods Clin Dev, 2025. 33(4): p. 101571.
7. Ceyhan-Birsoy, O., et al., Recessive truncating titin gene, TTN, mutations presenting as centronuclear myopathy. Neurology, 2013. 81(14): p. 1205-14.
8. Carmignac, V., et al., C-terminal titin deletions cause a novel early-onset myopathy with fatal cardiomyopathy. Ann. Neurol., 2007. 61(4): p. 340 - 351.
9. Rees, M., et al., Making sense of missense variants in TTN-related congenital myopathies. Acta Neuropathol, 2021. 141(3): p. 431-453.
10. Di Feo, M.F., et al., Inferring disease course from differential exon usage in the wide titinopathy spectrum. Ann Clin Transl Neurol, 2024.
11. Di Feo, M.F., et al., The crucial role of titin in fetal development: recurrent miscarriages and bone, heart and muscle anomalies characterise the severe end of titinopathies spectrum. J Med Genet, 2023.

Reviewer #3

(Remarks to the Author)
Overall, this study provides the basis of an interesting paper about the organ-scale effect of acute changes in the mechanical state of cardiomyocytes. However there were still some concerns with the study and data interpretation.

Major Concerns:
1. The authors strongly implicate cell-cell and cell-ECM interactions as the drivers of increased wall thickening and altered geometry. Is it possible that altered sarcomere lattice spacing and maintenance of constant sarcomeric volume expands myocardial tissue in the absence of sarcomere addition (hypertrophy) as an underlying determinant. Dose-response measurements of (A) sarcomere lattice spacing and (B) sarcomere lengths as a function of titin cleavage in relaxed and diastolic conditions would provide further mechanistic insight towards the change in structure but also strengthen the elastic recoil interpretation.
2. Related to concern #1, it is surprising that resting sarcomere lengths at diastolic [Ca2+] aren't changed (at least based on the data shown). (A) What is keeping the sarcomeres from being trapped in a short state? (B) How does titin cleavage impact the pressure-volume relationship over a series of contractions?
3. The authors suggest that a major finding of this study is "the prompt activation of fibroblasts and initiation of ECM remodeling following titin cleavage." Yet the authors show no quantitative evidence of fibroblast activation, other than a single image, nor do any time-resolved analysis of this activation. To make this claim, the authors need should quantify the number of PDGFRa+ cardiac fibroblasts that also express Postn+ and/or aSMA+ fibroblasts in the heart at multiple time points after cleavage. In addition, Postn is typically secreted and while the authors have a data figure showing a cell with intracellular Postn expression this approach will likely under-represent activated fibroblasts.
4. (A) Does remodeling of the ECM provide some elastic recoil that prevents the myocytes from totally collapsing to a short state?
5. The authors claim that "Ki67-positive cells began to rise as early as D4" but show no data. The authors should quantify the timing of proliferation and utilize additional cell-specific markers to define which cell types are expressing Ki67 after titin cleavage.
6. There are some concerns with the interpretation of the following data:
a. The "perinuclear" Integrin a5B1 staining is more likely staining interstitial cells (ie fibroblasts) that have a lot of integrin all over their cell surface. Additional staining should be done with cell-specific markers targeted to the membrane to properly interpret the observation.
b. The accumulation of WGA between the myocytes in the TEV mice is likely staining scar tissue, which the authors should comment on.
c. The TUNEL assay needs a counter-stain to determine which cardiac cell types are undergoing apoptosis. Furthermore, the authors should quantify cTEV+ myocytes (with an additional TUNEL costain) at each timepoint to show what population of the cells that have titin cleavage undergo apoptosis. Much of the compensatory mechanism by day 13 post-TEV may be due to an increased loss of myocytes.
7. The data seems to indicate destabilization or increased sarcomere protein turnover, with more highly expressed sarcomeric proteins and more ubiquitin degradation, which may confound the conclusions. To address this concern, the authors should experimentally should determine if there (a) is a change in titin half life due to increased degradation, (b) is a

change in total sarcomere content (i.e. less units of contraction per cardiac myocyte).

Minor comments:
1. The use of t-tests in figure 2B-G is inappropriate, and should instead be a two-way ANOVA with multiple comparisons to measure the TEV vs timepoint effects.
2. It would be helpful if the subfigures were ordered linearly with their labels.

Version 1:

Reviewer comments:

Reviewer #1

(Remarks to the Author)
I have reviewed the revised manuscript and the authors' response to the previous comments.

In my view, the authors have addressed the major concerns in a careful and constructive way. The revised version is clearly improved, particularly regarding the quantitative support of the main findings, the interpretation of fibroblast activation, and the analysis of intercalated-disk changes.

The additional data and clarifications now provide a stronger basis for the proposed sequence of events after titin cleavage. I also appreciate that the discussion has been toned down in several places, which makes the interpretation more balanced and more convincing.

I have no major further comments

Reviewer #2

(Remarks to the Author)
The revised manuscript by Freund and coworkers has been significantly improved, starting with a much clearer introduction and ending in a similarly improved discussion. It explains the rationale for cleaving titin in the I-band not far from the physiological T2 site, thereby mimicking the proteolytic titin cleavage observed also in ischemic or chemotherapy-induced cardiac injury and giving insight into I-band truncations in hereditary cardio/myopathies. The results suggest that the precise cleavage location is likely less critical than disruption of the elastic I-band continuity, and it is therefore intriguing that fibrosis sets on just above the natural T1/T2 ratio of about 20%.
The integration of data across scales, from cells to whole animal physiology, remains a major strength of the paper. The single-cardiomyocyte assays showed clearly that titin cleavage reduces resting sarcomere length following rapid relaxation after contraction, which is consistent with the reduced left-ventricular cavity size observed in vivo.

Several important experimental observations have been additionally highlighted, like the fnding that TUNEL-positive cells were fibroblasts, not cardiomyocytes or that the autophagy adapter SQSTM1 was upregulated in Hom, but not Het hearts.

The new expanded section on deregulation of cell-cell connectivity shows that loss of titin stiffness and the resulting force imbalance compromises sarcomere-intercalated disc connectivity, thus leading to cardiomyocyte detachment, disrupting the mechanical homeostasis sensed by fibroblasts and triggering matrix remodelling.

Insisting on clarifying the domain nomenclature was not malicious capriciousness on behalf of the reviewer. Thank you for clarifying that these are the domains encoded by TTN exons 225 and 226 encoding Ig86 and Ig87, alleviating the confusion in Rivas-Pardo et al. (2020), who were using both I86 and I87 and Ig86 and Ig87, mirroring the nomenclature by Bang et al. (2001) who assigned I to domains presumed to be in the I-band. This numbering is at least 9 domains offset to the contiguous "Ig" nomenclature that avoids such confusion, especially in the differentially spliced regions.

Reviewer #3

(Remarks to the Author)
The authors have adequately addressed my concerns. This is a great study and will be impactful for the field.

Open Access This Peer Review File is licensed under a Creative Commons Attribution 4.0 International License, which permits use, sharing, adaptation, distribution and reproduction in any medium or format, as long as you give appropriate credit to the original author(s) and the source, provide a link to the Creative Commons license, and indicate if changes were made.
In cases where reviewers are anonymous, credit should be given to 'Anonymous Referee' and the source.
The images or other third party material in this Peer Review File are included in the article's Creative Commons license, unless indicated otherwise in a credit line to the material. If material is not included in the article's Creative Commons license and your intended use is not permitted by statutory regulation or exceeds the permitted use, you will need to obtain permission directly from the copyright holder.
To view a copy of this license, visit https://creativecommons.org/licenses/by/4.0/

Response to Editors' comments

"More specifically, it will be particularly important to the editors that you address the following concerns:
1 To provide direct mechanistic evidence of the effects of titin cleavage on fibroblast activation, please quantify PDGFRα$^+$ cardiac fibroblasts co-expressing Postn$^+$ and/or αSMA$^+$ at multiple time points following cleavage.(R#1,3)"*

In response to this important point, we performed additional immunofluorescence experiments demonstrating that the vast majority of periostin-positive fibroblasts also express PDGFRα, indicating a high degree of overlap between these populations (Fig. 5h; p.11, lines 5-8). Based on these data, we conclude that periostin detection does not under-represent activated fibroblasts in our experimental setting.

To provide quantitative evidence of fibroblast activation, we additionaly assessed periostin expression by Western blot analysis, which revealed a significant upregulation of periostin at day (D)6 and D13 post-TEV injection (Fig. 5g; p.11, lines 4-5).

In addition, we expanded our analysis of fibroblast proliferation: whereas Ki67$^+$ fibroblasts were previously quantified at D6 and D13, we now included an earlier time point (D4-5) for TEV-injected TC mice (Fig. 5a and d). As done previously for D6, heart tissues at D4-5 were also co-stained for Ki67 and periostin, confirming that Ki67$^+$ cells are myofibroblasts (Fig. 5f; p.11, lines 3-4). In summary, this extended analysis clearly demonstrates increased fibroproliferation already at very early stages after cleavage (Fig. 5a and d; p. 11, lines 8-9).

*"2*Please quantify connexin-43 or desmosomal markers to support intercalated-disk changes; (R#1)"*

In response to this comment, we have added quantitative analyses of intercalated disk components. Specifically, the "jaggedness" of these components in IF images was analyzed by calculating the root-mean-square (RMS) deviation of desmoplakin and N-cadherin signals, presented in Fig. 6b and c. In addition, RMS deviation of connexin-43 and a quantification of the connexin-43-positive area on IF images are shown in Fig. 6e, collectively providing a robust assessment of intercalated disk changes following TEV-injection (p. 12, lines 3-9). Furthermore, we have additionally quantified the intracellular versus extracellular area occupied by integrin α5β1 complexes on IF images (Fig. 6g; p.12, lines 19-20). All new data support intercalated disk and integrin changes as an immediate consequence of titin cleavage.

"3 To increase translational relevance, please include a discussion on known titin cleavage patterns in human cardiomyopathy (R#1)"*

We have addressed this point in the revised manuscript in two places.

First, in the introduction, we now cite studies reporting known titin cleavage patterns in unrelated cardiac conditions, such as ischemic and chemotherapy-induced cardiomyopathy (p. 3, lines 16-22). We state that the well-known proteolytic "T2" titin product arises due to cleavage in the mid-I-band region, near our TEV cleavage site. The appearance of T2 titin has widely been described for healthy and diseased myocardium, including human hearts, and its expression has recently been quantified by us in a cohort of ~120 human heart tissues (Fomin et al., *Sci. Transl. Med.*, 13:eabd3079, 2021; our Ref 21). We point out that T2 titin is thought to arise from calpain- and MMP-mediated cleavage and that it has been proposed to reflect sarcomere turnover and

repair, while it is elevated under oxidative stress conditions in failing hearts. Our TEV cleavage model essentially mimics this situation and therefore is of high translational relevance.

Second, in the discussion, we expand on the translational implications, emphasizing the likelihood of a potential critical threshold of titin cleavage between physiological and pathological states, and the opportunities for targeted interventions aimed at modulating titin-based stiffness in acquired cardiomyopathies (Discussion, p. 16, lines 1-11): *"Our data also suggest a threshold beyond which titin cleavage triggers pathological remodeling. Fibroblast proliferation increased at only 12-20% titin cleavage (D4-5), preceding measurable functional decline. This range closely approximates T2 titin proportions in human hearts (12-14%)[21], suggesting tightly regulated physiological titin turnover. Excess proteolysis, for example during oxidative stress, may shift this balance toward fibroblast activation and fibrosis. In healthy myocardium, limited titin cleavage likely supports protein turnover without triggering pathological remodeling. In the TC model, cardiac function and geometry remained largely preserved until cleavage exceeded ~30%, delineating a potential therapeutic window. These findings may guide targeted interventions for acquired cardiomyopathies[17,19], atrial fibrillation16, and RBM20-associated inherited cardiomyopathies[14,47], and support moderate titin de-stiffening strategies in HFpEF, such as controlled upregulation of N2BA isoforms via RBM20 modulation[54,55]."*)

"4 To further contextualize your main findings in relation to inherited cardiomyopathies, please include a discussion that references relevant genetic disorders and their potential association with spontaneous abortions, highlighting the clinical implications (R#2)"*

We agree with Reviewer 2 that our model is not a model of *TTN* truncation cardiomyopathy, although hypothetical biallelic I-band truncations could show similar pathology, and would likely be causing prenatal death/spontaneous abortions.

We have incorporated this point into the discussion as follows (p. 14, lines 18-24): *"Although the TC mouse model does not replicate TTN truncation disorders, which are typically caused by heterozygous truncating variants[10,21,48], it provides insight into potential consequences of hypothetical biallelic I-band truncations. Such variants would be incompatible with life because titin could no longer maintain sarcomere integrity and cardiac mechanical function, resulting in intrauterine lethality. Titin defects are implicated in spontaneous abortion, although their prevalence is unclear[49]. Prenatal TTN-associated phenotypes extend beyond cardiac abnormalities to skeletal muscle, joint, and bone defects[49-51]."*

"5 Please include dose-response measurements of sarcomere lattice spacing and sarcomere lengths, and measurements of the diastolic conditions (R#3)"*

This is a very good point, and we thank the reviewer for this insightful suggestion. Rather than presenting additional lattice spacing data in the current manuscript, we now refer to our recent publication (Hessel et al, *Circ. Res.* 134:1026-1028, 2024), in which we demonstrate the effect of targeted titin cleavage (ex vivo) on stretch-dependent sarcomere lattice spacing in TC cardiac fibers using small-angle X-ray diffraction (p.13, line 24 to p.14, line 2).

In addition, the effects of titin cleavage on diastolic sarcomere length are presented in the current manuscript using single-cardiomyocyte mechanical assays (3d-f; p. 6, lines 6-13). The relevant text in the discussion (p.13, line 24 to p.14, line 2) now reads: *"Single-cardiomyocyte assays revealed that titin cleavage reduces resting SL following rapid relaxation after contraction, consistent with the reduced LV cavity size observed in vivo. The accompanying increase in LV wall thickness may partly reflect increased sarcomere diameter at shorter lengths, resulting from expansion of myofilament*

lattice spacing. Titin contributes to lattice spacing control, whereas titin cleavage reduces transverse sarcomere stiffness[45] and further expands lattice spacing[46], thereby promoting wall thickening."

"6 Please discuss whether the TEV recognition sites introduced in the I-band are relevant for any disease, or if these are close to any physiological cleavage site (R#2)"*

In the introduction, we refer to native cleavage patterns described under common disease conditions, explicitly mentioning ischemic damage, chemotherapy-induced cardiotoxicity, and atrial fibrillation, where calpains/MMPs are targeted to cleavage sites in the I-band (p. 3, lines 13-22). Furthermore, we now refer to the frequently described native cleavage site(s) in mid-I-band titin that causes the appearance of "T2" titin and a Z-disk-anchored counterpart, very similar to our TC cleavage model. The role of these cleaved titin fragments under healthy conditions and the possible pathological relevance are incompletely understood (p. 3, lines 20-22). Our TC model provides the first in-depth functional analysis of the consequence of mid-I-band titin cleavage. Importantly, T2 titin makes up 12-14% of total titin protein in endstage failing human hearts and is increased with oxidative stress-induced proteolytic activity increase.

The relevant text in the introduction now reads (p. 3, lines 13-22): *"Equally relevant, yet less explored, is the increased proteolytic cleavage of titin's elastic I-band by calpains and matrix metalloproteinases (MMPs) under common disease conditions, including atrial fibrillation[16], post-ischemic injury[17,18], and chemotherapy-induced cardiotoxicity[19,20]. Notably, an "enigmatic" proteolytic cleavage site resides in the mid-I-band titin region and gives rise to the appearance of "T2" titin on loose gels. T2 titin comprises the A-band and adjacent spring fragment and accounts for approximately 12-14% of total titin protein in endstage failing human hearts[21]. T2 titin is thought to arise from calpain- and MMP-mediated cleavage and has been proposed to reflect sarcomere turnover and repair[16,17]. However, the (patho)physiological relevance of T2 titin and its Z-disk-anchored counterpart, particularly with respect to titin's mechanical function, remains poorly understood."*

Furthermore, in the discussion, we clarify as follows (p. 14, lines 14-15): *"…Importantly, these changes can be triggered by proteolytic cleavage at a single I-band titin site. The precise cleavage location is likely less critical than disruption of elastic I-band continuity. MMP2 cleaves titin in the mid I-band near our TEV site, generating similar T2 fragments (~2.2-2.4 MDa)[17] and is therefore predicted to produce comparable structural and functional consequences in stressed human hearts."*

"7 Please include identification of the cleaved I-band fragments either using Fragment-specific antibodies or mass spectroscopy (R#2)"*

We would like to respectfully note that the identification of the cleaved I-band fragments was already included in the original submission. In the revised manuscript, Western blot analysis using fragment-specific antibodies is shown in Fig. S1, confirming the presence of the expected cleaved fragments (p. 5, lines 15-17).

Response to Reviewers' comments

Response to Reviewer #1

"The study caught my attention, mainly because it connects a defined mechanical disturbance to a recognizable cardiac phenotype. The model is elegant, the dataset extensive, and the topic relevant well beyond basic muscle mechanics.
The story is convincing in parts but slightly overstated in others. If the authors can tighten the data presentation and refine the tone of the discussion, this could be a very solid paper.

Major Comments
1. Clarifying the main sequence of events:
It would help to describe when the key changes occur – titin cleavage, mechanical failure, fibrosis. Even a short schematic or paragraph could show whether the loss of elasticity precedes the structural response."

Thank you for your suggestion. A schematic summarizing the sequence of myocardial remodeling events following titin cleavage is now provided in the new Fig. S8.

"2. Quantitative clarity
Some central figures (e.g. Fig. 3–5, Suppl. S5–S7) show examples without sufficient numbers. Please include n-values, error bars, and the type of statistical test. Small details like this raise confidence."

We agree with the reviewer and have made every effort to include N-values (or n-values), error bars, and the type of statistical test used in all figure legends. We believe that the sample sizes and statistical analyses provided are sufficient to support meaningful and robust conclusions.

"3. Fibrosis as consequence, not cause
The link between titin cleavage and fibroblast activation feels mostly correlative. A short note acknowledging that would already help; direct mechanistic evidence would make it even stronger."

We consent and have added the following statement to the discussion (p. 15, lines 7-10):

"Although the precise mechanisms linking titin cleavage to fibrosis remain incompletely defined, reduced titin stiffness and force imbalance clearly destabilize integrin- and Cx43-mediated signaling, possibly causing local matrix stiffening – initially without overt fibrotic deposition – which is sensed by fibroblasts, as recently reported in a model of sarcomeric hypocontractility[30]."

At the same time, we have added additional data and analyses strengthening our mechanistic insight (Fig. 5 additions and new Fig. 6). With this, we propose a direct link between titin cleavage and fibroblast activation, as follows, on p. 11, lines 23-25:

"Thus, titin cleavage leads to mechanical disconnection and imbalance, rapidly activating fibroblasts and subsequently promoting interstitial fibrosis as a maladaptive remodeling response."

as well as on p. 12, lines 23-26:
"Taken together, loss of titin stiffness and the resulting force imbalance compromise sarcomere-ICD connectivity, promote lateral cardiomyocyte detachment, and disrupt cardiomyocyte-matrix links, thereby destabilizing the mechanical homeostasis sensed by fibroblasts and triggering matrix remodeling."

"4. Intercellular coupling
The intercalated-disk changes are striking. Quantifying connexin-43 or desmosomal markers, if feasible, would support the interpretation. If not, a brief comment on the limitation would be fine."

In response, we have added quantitative analyses of intercalated disk components. Specifically, the "jaggedness" of the desmoplakin, N-cadherin, and connexin-43 signals in IF images was quantified as the root-mean-square (RMS) deviation of ICD staining (Fig. 6b-c, e). In addition, the connexin-43-positive area percentage was quantified (Fig. 6e), together providing a robust assessment of intercalated disk changes following TEV injection (p. 12, lines 3-9). Furthermore, we quantified the redistribution of cardiomyocyte integrin a5b1 from intracellular to extracellular compartments (Fig. 6g). All new data support intercalated disk and integrin alterations as immediate consequences of titin cleavage.

"5. Physiological angle
The predominance of diastolic dysfunction makes good sense – titin mainly affects passive tension. It might help to mention that explicitly and to note that fibrosis likely amplifies stiffness later on."

We now make such a statement in the manuscript. In measurements of passive tension in in vivo-cleaved cardiac fiber bundles (Fig. 3g), we found that titin cleavage primarily reduced myocardial passive tension at D6 post-TEV injection, whereas a marked increase in stiffness at D13 likely reflects reactive extracellular matrix remodelling (p. 8, lines 16-20).

"6. Human or translational link
A short reference to any known titin-cleavage patterns in human cardiomyopathy would strengthen the relevance."

We have addressed this point in the revised manuscript in two places.

First, in the introduction, we cite studies reporting known titin cleavage patterns in unrelated cardiac conditions, such as ischemic and chemotherapy-induced cardiomyopathy (p. 3, lines 16-22). We state that the well-known proteolytic "T2" titin product arises due to cleavage in the mid-I-band region, near our TEV cleavage site. The appearance of T2 titin has widely been described for healthy and diseased myocardium, including human hearts, and its expression has recently been quantified by us in a cohort of ~120 human heart tissues (Fomin et al., *Sci. Transl. Med.*, 13:eabd3079, 2021; our Ref 21). We point out that T2 titin is thought to arise from calpain- and MMP-mediated cleavage and that it has been proposed to reflect sarcomere turnover and repair, while it is elevated under oxidative stress conditions in failing hearts. Our TEV cleavage model essentially mimics this situation and therefore is of high translational relevance.

Second, in the discussion, we expand on the translational implications, emphasizing the likelihood of a potential critical threshold of titin cleavage between physiological and pathological states, and the opportunities for targeted interventions aimed at modulating titin-based stiffness in acquired cardiomyopathies (Discussion, p. 16, lines 1-11):

"Our data also suggest a threshold beyond which titin cleavage triggers pathological remodeling. Fibroblast proliferation increased at only 12-20% titin cleavage (D4-5), preceding measurable functional decline. This range closely approximates T2 titin proportions in human hearts (12-14%)[21], suggesting

tightly regulated physiological titin turnover. Excess proteolysis, for example during oxidative stress, may shift this balance toward fibroblast activation and fibrosis. In healthy myocardium, limited titin cleavage likely supports protein turnover without triggering pathological remodeling. In the TC model, cardiac function and geometry remained largely preserved until cleavage exceeded ~30%, delineating a potential therapeutic window. These findings may guide targeted interventions for acquired cardiomyopathies[17,19], atrial fibrillation16, and RBM20-associated inherited cardiomyopathies[14,47], and support moderate titin de-stiffening strategies in HFpEF, such as controlled upregulation of N2BA isoforms via RBM20 modulation[54,55].")

"7. Tone of the discussion
A few sentences sound a bit categorical ("titin cleavage causes heart failure"). Softer phrasing would make the argument more persuasive."

In the revised manuscript, we have been careful to avoid overly categorical statements and to ensure a balanced and appropriately cautious interpretation of the data.

"Minor Comments:
- *Improve axis labeling and add units (e.g. Fig. 2, Fig. 5)."*

We appreciate the Reviewer's comment. We would like to point out that parameters shown without units (such as in Fig. 2 and Fig 5) report ratios or dimensionless numerical values. For instance, the echocardiographic parameter RWT (Fig. 2i) was calculated as follows (p. 28, lines 43-45):
"Relative wall thickness (RWT) was calculated as twice the LVPWd divided by the LV end-diastolic internal diameter (LVIDd) (RWT = 2 x LVPWd / LVIDd)."

"- Define all error bars; specify whether normality was tested"

All error bars are now clearly defined. The statistical analysis approaches, including tests for normality, is described in the Statistics section (p. 33, lines 30-38), and the figure legends specify the statistical tests used.

"- Add brief captions to supplementary movies."

Captions are provided in the appropriate space on the Nature CVR submission site and also in the file 'Extended Figures and Table' on p. 11.

Response to Reviewer #2

"Striated muscles of heart and skeletal muscle systems are reliant on the highly regular, repetitive array of interdigitating, contractile actin and myosin filaments into the smallest contractile units of striated muscle, the sarcomeres. The largest protein on the vertebrate body, titin (encoded by the TTN gene) provides, amongst many assembly functions, a mechanical, elastic link between the actin and myosin filaments and thus assures the central position of myosin filaments at the onset of each contraction, the equal dissipation of imbalanced forces, and thus the mechanical integrity of the entire system. Loss of mechanical continuity of titin between the actin and myosin filaments in the I-band is therefore particularly destructive for striated muscle sarcomeres.
Previous work by other groups showed that cardiac stressors like ischemia/reperfusion-induced injury, inflammation and others lead to the activation of proteases that cleave several intracellular cytoskeletal proteins including titin, thus impairing the essential mechanical connectivity of contractile structures and the cytoskeleton. The results are serious contractile dysfunction and adverse cardiac remodelling, including the proliferation of fibroblasts and excessive, albeit possibly compensatory collagen deposition.
Freundt and co-workers have reduced the complexity of cardiomyocyte stress-induced proteolysis to a single target by genetically engineering mice to include the highly specific tobacco etch virus (TEV) restriction protease site into the I-band portion of titin. Virus-mediated expression of TEV protease under a highly cardiac-specific promoter then triggers relatively rapid and complete cleavage of I-band titin in a significant fraction of cardiomyocytes. This experimental design should therefore allow attributing any cellular and physiological changes solely to the cleavage of titin and not any other cytoplasmic or extracellular protease. This complex and comprehensive study is of very high technical quality, well-written and well-illustrated.
Remarkably, the authors observe that I-band titin cleavage of only a relatively small proportion of cardiomyocytes alone suffices to trigger rapid cardiac failure, fibrotic remodelling of the heart, and pathological remodelling of cardiomyocyte cell-cell contacts at the intercalated disks. These important findings demonstrate beyond reasonable doubt that titin is directly involved in cellular communication that converts information on mechanical stress into signals communicating with the extracellular compartments and that its cleavage alone suffices to trigger a rapid fibrotic response of the cardiac fibroblast population.
These are important and novel findings that will be of interest to the readers of Nature Cardiovascular Research.

There are some important questions about the experimental design, the interpretation and generalisation of the results, however.

1. There is some possible confusion about the numbering of titin domains. According to the primary reference by Rivas-Pardo, the TEV site was introduced between titin I-band domains I86 and I87, not immunoglobulin domains 86-87 as stated here. Please use the titin domain numbering referenced to the IC sequence, which is less ambiguous (due to alternative splicing) than I-band domain number, see e.g. [1] and online titin data base resources. In the complete titin domain array, the two domains are Ig-100 and Ig-101 in the distal I-band."

We have re-checked how the TEV insertion position was described in Rivas-Pardo et al. (2020), where we first described this genetic mouse model, and found no discrepancies. To further clarify the exact position of the TEV cassette within the titin spring region, we now also refer to the *TTN* exon positions and state in the Results (p. 4, lines 19-21):

*"To test this, we employed the titin cleavage (TC) mouse model[32], inserting a tobacco etch virus protease (TEV) recognition site into titin's I-band region between TTN exons 225 and 226, encoding immunoglobulin-like domains 86 and 87 (**Fig. 1a**)."*

In addition, in the Methods section, we have added the exact amino acid residues for the domains I86 and I87, together with the UniProt identifier (p. 28, lines 22-24):

"The TC mouse model[32] contains a TEV protease recognition site and a HaloTag cassette near titin's I-band/A-band junction between Ig domains I86 (amino acids 14072-14157) and I87 (residues 14161-14246; UniProt A2ASS6-1)."

"2. Why was the TEV recognition site introduced into titin's I-band region between immunoglobulin-like domains 100 and 101? Does this mirror any known cleavage site, (if yes, please reference) or is the genetic mouse model used, as the primary publication by Rivas-Pardo 2020 suggests, designed for a different and purely fundamental question? Are there any data on physiological cleavage between or near domains I-86 and 87? If the model does not mirror a mapped disease-linked cleavage site, what is its disease relevance? Where is the closest mapped proteolytic titin cleavage site, and would this influence the interpretation of the data? As I-band titin is hypothesised to interact with actin filaments under certain conditions as [2-4], the position of physiological or non-physiological cleavage would likely matter for the resulting mechanobiological defects. Metalloprotease cleavage as identified by [5] is N-terminal to the TEV site used here. It would be helpful to discuss these points."

To address this point, we have added additional information to both the introduction and discussion of the revised manuscript. In the introduction, we now refer to a "classical" native cleavage pattern that our model is designed to mimic, namely, mid-I-band cleavage site(s) that give rise to the proteolytic titin fragment T2.

The T2 fragment appears under a range of common disease conditions, including ischemic injury, chemotherapy-induced cardiotoxicity, and atrial fibrillation, and is thought to result from the activity of calpains and MMPs (p. 3, lines 13-22). T2 titin has a molecular size (2.2-2.4 MDa) very similar to that of our TEV-cleaved-A fragment. Importantly, T2 titin accounts for 12-14% of total titin protein in endstage failing human hearts (Fomin et al., *Sci. Transl. Med.*, 13:eabd3079, 2021; our Ref 21) and increases with oxidative stress-induced proteolytic activity. The physiological and pathophysiological roles of the T2 titin fragment (and its Z-disk-anchored counterpart) remain incompletely understood (p. 3, lines 20-22). Our TC model provides the first in-depth functional analysis of the consequence of mid-I-band titin cleavage and is therefore of translational relevance.

The relevant text in the introduction now reads (p. 3, lines 13-22):

"Equally relevant, yet less explored, is the increased proteolytic cleavage of titin's elastic I-band by calpains and matrix metalloproteinases (MMPs) under common disease conditions, including atrial fibrillation[16], post-ischemic injury[17,18], and chemotherapy-induced cardiotoxicity[19,20]. Notably, an "enigmatic" proteolytic cleavage site resides in the mid-I-band titin region and gives rise to the appearance of "T2" titin on loose gels. T2 titin comprises the A-band and adjacent spring fragment and accounts for approximately 12-14% of total titin protein in endstage failing human hearts[21]. T2 titin is thought to arise from calpain- and MMP-mediated cleavage and has been proposed to reflect sarcomere turnover and repair[16,17]. However, the (patho)physiological relevance of T2 titin and its Z-disk-anchored counterpart, particularly with respect to titin's mechanical function, remains poorly understood."

In our view, the precise cleavage location within titin's I-band is likely less critical for the observed effects than disruption of elastic I-band continuity itself. Accordingly, we now clarify this point in the discussion (p. 14, lines 14-15):

"…Importantly, these changes can be triggered by proteolytic cleavage at a single I-band titin site. The precise cleavage location is likely less critical than disruption of elastic I-band continuity. MMP2 cleaves titin in the mid I-band near our TEV site, generating similar T2 fragments (~2.2-2.4 MDa)[17] and is therefore predicted to produce comparable structural and functional consequences in stressed human hearts."

In addition, I-band titin in cardiac muscle – particularly the N2B isoform – has substantially lower affinity for actin filaments than the N2A isoform in skeletal muscles. Consequently, the N2B spring recoils toward the Z-disk following I-band cleavage (Li et al., *Elife* 2020; our Ref. 35). The N2B isoform (much more than N2BA titin) is considered primarily responsible for the bidirectional spring properties underlying titin-based elastic recoil of sarcomeres.

Furthermore, in the discussion we expanded on the translational implications, emphasizing the likelihood of a critical threshold separating physiological from pathological titin cleavage (Discussion, p. 16, lines 1-7):

"Our data also suggest a threshold beyond which titin cleavage triggers pathological remodeling. Fibroblast proliferation increased at only 12-20% titin cleavage (D4-5), preceding measurable functional decline. This range closely approximates T2 titin proportions in human hearts (12-14%)[21], suggesting tightly regulated physiological titin turnover. Excess proteolysis, for example during oxidative stress, may shift this balance toward fibroblast activation and fibrosis. In healthy myocardium, limited titin cleavage likely supports protein turnover without triggering pathological remodeling."

"3. The cleaved-I is assigned to two corresponding to N2BA and N2B: how were these unambiguously identified beyond sizes on a gel? Fragment-specific antibodies or mass-spectroscopy data would be supportive of the assignment. Please make this clearer."

We would like to respectfully note that the identification of the cleaved I-band fragments was already included in the original submission. In the revised manuscript, Western blot analysis using fragment-specific antibodies is shown in Fig. S1, confirming the presence of the expected cleaved fragments (p. 5, lines 15-17).

"4. The AAV9-mediated TEV expression under a cardiac troponin-T promoter is assumed to be completely heart specific, however, the pleiotropism of AAV9 is a significant issue for gene therapy approaches [6]. Please comment whether any off-site expression (Liver?? Skeletal muscle?) could contribute to the observed phenotypes and how this was controlled and ruled out."

We fully agree with the reviewer's comment regarding the potential pleiotropism of the AAV9 vector. We carefully evaluated the cardiac specificity of our approach by quantifying (by WB) GFP expression in the heart and other organs following injection of an AAV9-GFP control vector. The relevant analyses are shown in Fig. 1e and Fig S1a (as in the original manuscript). We observed only minimal off-target expression in other organs, including liver and spleen. We report this in the revised manuscript (p. 5, lines 3-5):

*"Western blots (WBs) using tissue from GFP-injected mice confirmed cardiac-restricted expression: GFP was evident in all heart chambers yet nearly absent in skeletal muscle, spleen, and even liver (**Fig. 1e, Fig. S1a**)."*

"5. The authors laudably resist the temptation of discussing the relevance of their experimental findings for inherited cardiomyopathies. Proteolytic cleavage of I-band titin would reflect homozygous truncating variants in this region. Albeit biallelic truncating variants in titin can cause severe early-onset myopathies with cardiomyopathy, at least one truncation is always in the A-band or M-band, thus maintaining mechanical connectivity between thick and thin filaments [7-10]. Consequently, in human muscle with biallelic truncating variants studied, titin remained A-band bound and does not recoil to the Z-disk. Dilated cardiomyopathy due to truncating titin mutations is heterozygous, i.e. one allele is normal, while the majority of mutations cluster in the A-band region, therefore providing mechanical continuity because they are retained (albeit likely weakened), as in early onset myopathies. No human disease-linked I-band homozygous or compound-heterozygous variants have been reported, possibly because of intrauterine lethality, as would be inferred from the complete loss of sarcomere integrity after I-band cleavage. Although genetic truncation, manifest over long time and possible mitigated by differential splicing, is not directly comparable to acute proteolytic cleavage, a short discussion with reference to genetic disorders and spontaneous abortions e.g. [11] would be helpful."

We agree with the reviewer that our model is not a model of *TTN* truncation cardiomyopathy, although hypothetical biallelic I-band truncations could show similar pathology, and would likely be causing prenatal death/spontaneous abortions.

We have incorporated this point into the discussion as follows (p. 14, lines 18-24):

"Although the TC mouse model does not replicate TTN truncation disorders, which are typically caused by heterozygous truncating variants[10,21,48], it provides insight into potential consequences of hypothetical biallelic I-band truncations. Such variants would be incompatible with life because titin could no longer maintain sarcomere integrity and cardiac mechanical function, resulting in intrauterine lethality. Titin defects are implicated in spontaneous abortion, although their prevalence is unclear[49]. Prenatal TTN-associated phenotypes extend beyond cardiac abnormalities to skeletal muscle, joint, and bone defects[49-51]."

"Minor points
It is remarkable that the proteomic analysis shows several upregulated ubiquitin ligases (TRIM33, TRIM35, TRIM47). None are "classical" sarcomere-associated tripartite motif family ligases, please comment on their possible implication in sarcomere instability. TRIM35 appears highlighted three times in Figure S5e. Please check if this is a mistake."

Thank you for this insightful comment. The deregulation of several ubiquitin ligases following titin cleavage is intriguing. As shown in Fig. S7d, E3 ligases known to be expressed in the heart or skeletal muscle, such as Atrogin1, CHIP, and MuRF1, were upregulated in titin-cleaved versus GFP-injected (homozygous) TC LV, likely explaining the increased ubiquitination of cleaved titin shown in Fig. S7b, c. In the proteomic analysis, CHIP (Stub1) was also significantly upregulated. The concurrent upregulation of ubiquitin ligases not classically associated with muscle, such as TRIM32 and TRIM35 (Fig. 4e), may reflect the use of bulk proteomics rather than spatially resolved proteomics. Therefore, we cannot determine from these data whether these ligases directly contribute to the ubiquitination of sarcomeric proteins, including titin. Identification of sarcomere-relevant E3 ligases is beyond the scope of the current manuscript.

TRIM35 was highlighted more than once in Fig. 4e, because we distinguished between proteins deregulated on D6 (solid lines, circles) and D13 (dashed lines; squares as symbol). However, the additional reference to TRIM35 in the upper right corner was included in error. We thank the reviewer for identifying this mistake, which has now been corrected in the revised version. We have also carefully re-examined this and the other volcano plot figures to ensure that all displayed data are accurate. We sincerely apologize for this oversight.

Response to Reviewer #3

"Overall, this study provides the basis of an interesting paper about the organ-scale effect of acute changes in the mechanical state of cardiomyocytes. However there were still some concerns with the study and data interpretation.

Major Concerns:
1. The authors strongly implicate cell-cell and cell-ECM interactions as the drivers of increased wall thickening and altered geometry. Is it possible that altered sarcomere lattice spacing and maintenance of constant sarcomeric volume expands myocardial tissue in the absence of sarcomere addition (hypertrophy) as an underlying determinant. Dose-response measurements of (A) sarcomere lattice spacing and (B) sarcomere lengths as a function of titin cleavage in relaxed and diastolic conditions would provide further mechanistic insight towards the change in structure but also strengthen the elastic recoil interpretation."

This is a very good point, and we thank the reviewer for this insightful suggestion. Rather than presenting additional lattice spacing data in the current manuscript, we now refer to our recent publication (Hessel et al, *Circ. Res.* 134:1026-1028, 2024), in which we demonstrate the effect of targeted titin cleavage (ex vivo) on stretch-dependent sarcomere lattice spacing in TC cardiac fibers using small-angle X-ray diffraction (p.13, line 24 to p.14, line 2).

In addition, the effects of titin cleavage on diastolic sarcomere length are presented in the current manuscript using single-cardiomyocyte mechanical assays (3d-f; p. 6, lines 6-13). The relevant text in the discussion (p.13, line 24 to p.14, line 2) now reads:

"Single-cardiomyocyte assays revealed that titin cleavage reduces resting SL following rapid relaxation after contraction, consistent with the reduced LV cavity size observed in vivo. The accompanying increase in LV wall thickness may partly reflect increased sarcomere diameter at shorter lengths, resulting from expansion of myofilament lattice spacing. Titin contributes to lattice spacing control, whereas titin cleavage reduces transverse sarcomere stiffness[45] and further expands lattice spacing[46], thereby promoting wall thickening."

"2. Related to concern #1, it is surprising that resting sarcomere lengths at diastolic [Ca2+] aren't changed (at least based on the data shown). (A) What is keeping the sarcomeres from being trapped in a short state? (B) How does titin cleavage impact the pressure-volume relationship over a series of contractions?"

As noted above, sarcomeres do adopt a shorter resting length following cleavage, as expected (Fig. 3d-f).

While we did not directly measure pressure-volume relationships in these experiments, we would expect the end-systolic pressure–volume relationship (ESPVR) to become steeper. The end-diastolic pressure–volume relationship (ESPVR) would likely show little change initially but may become steeper over time as structural remodeling progresses.

"3. The authors suggest that a major finding of this study is "the prompt activation of fibroblasts and initiation of ECM remodeling following titin cleavage." Yet the authors show no quantitative evidence of fibroblast activation, other than a single image, nor do any time-resolved analysis of this activation. To make this claim, the authors need should quantify the number of PDGFRa+ cardiac fibroblasts that also express Postn+ and/or aSMA+ fibroblasts in the heart at multiple time points after cleavage. In addition, Postn is typically secreted and while

the authors have a data figure showing a cell with intracellular Postn expression this approach will likely under-represent activated fibroblasts."

In response to this important point, we performed additional immunofluorescence experiments demonstrating that the vast majority of Postn+ fibroblasts also expresses PDGFRα, indicating a high degree of overlap between these populations (Fig. 5h; p.11, lines 5-8). Based on these data, we conclude that periostin detection does not under-represent activated fibroblasts in our experimental setting.

To provide quantitative evidence of fibroblast activation, we additionally assessed periostin expression by Western blot analysis, which revealed a significant upregulation of periostin at D6 and D13 post-TEV injection (Fig. 5g; p.11, lines 4-5).

"4. (A) Does remodeling of the ECM provide some elastic recoil that prevents the myocytes from totally collapsing to a short state?"

We agree that ECM remodeling may contribute to elastic recoil and may help prevent sarcomeres from collapsing into an excessively shortened state. We believe that ECM remodeling likely contributes to the partial recovery of selected cardiac functional parameters observed at D13 and may partially restore elastic recoil. However, over the longer term, this maladaptive response is likely to promote further functional decline. In the manuscript, we now state (p. 15, lines 25-27):

"This ECM-driven stiffness increase can transiently stabilize myocardial geometry, function, and elastic recoil, as reflected by partial recovery of echocardiographic parameters on D10-13, but cannot prevent rapid decompensation."

"5. The authors claim that "Ki67-positive cells began to rise as early as D4" but show no data. The authors should quantify the timing of proliferation and utilize additional cell-specific markers to define which cell types are expressing Ki67 after titin cleavage."

In response to this comment, we have now included quantification of $Ki67^{+}$ cells at D4-5 post-TEV injection (Fig. 5a and d). As previously performed for D6, tissue samples at D4-5 were also co-stained for Ki67 and periostin, confirming that the Ki67+ cells predominantly represent myofibroblasts (Fig. 5f; p.11, lines 3-4).

"6. There are some concerns with the interpretation of the following data:
a. The "perinuclear" Integrin a5B1 staining is more likely staining interstitial cells (ie fibroblasts) that have a lot of integrin all over their cell surface. Additional staining should be done with cell-specific markers targeted to the membrane to properly interpret the observation."

In response to this comment, we performed co-staining of integrin α5β1 with the cardiomyocyte marker PCM1 and the fibroblast marker PDGFRα (Fig. 6i). The IF images demonstrate that integrin α5β1 localizes to the perinuclear space specifically in $PCM1^{-}$/ $PDGFR\alpha^{+}$ fibroblasts, indicating that the observed staining reflects fibroblast-specific perinuclear localization rather than cardiomyocytes or a generalized cell-surface signal (p. 12, lines 21-23).

"b. The accumulation of WGA between the myocytes in the TEV mice is likely staining scar tissue, which the authors should comment on."

In our view, the increased WGA signal between the myocytes after titin cleavage does not represent scar tissue, which typically results from the replacement of necrotic cardiomyocytes by extracellular matrix material. In our manuscript, we show no evidence of increased cardiomyocyte loss; instead, cardiomyocyte numbers remain stable (Fig. 5c). We interpret the WGA accumulation as a consequence of severely disrupted inter-cardiomyocyte connectivity, leading to loss of tight alignment between adjacent cell borders. Consistent with this interpretation, electron micrographs show no collagen deposition between titin-cleaved cardiomyocytes, despite the widened WGA-positive areas observed as early as D4-5 (data not included in the manuscript). To clarify this point, we have added the following statement to the revised manuscript (p. 11, lines 18-21):

*"Strikingly, WGA staining revealed a greatly enlarged inter-cardiomyocyte space at D6 and D13 (**Fig. 5l**), primarily reflecting loss of cell-cell contact rather than scar tissue formation. These expanded gaps are likely to contribute to the LV wall thickening observed in titin-cleaved hearts."*

"c. The TUNEL assay needs a counter-stain to determine which cardiac cell types are undergoing apoptosis. Furthermore, the authors should quantify cTEV+ myocytes (with an additional TUNEL costain) at each timepoint to show what population of the cells that have titin cleavage undergo apoptosis. Much of the compensatory mechanism by day 13 post-TEV may be due to an increased loss of myocytes."

We have now combined the TUNEL assay with anti-cTEV staining to identify titin-cleaved cardiomyocytes, and with anti-periostin staining to label myofibroblasts (Fig. S7a). These experiments show that apoptotic cells are exclusively fibroblasts (p. 10, lines 3-7). Thus, there is no evidence of increased myocyte loss, in line with stable cardiomyocyte numbers observed at D13 post-TEV injection (Fig. 5c).

"7. The data seems to indicate destabilization or increased sarcomere protein turnover, with more highly expressed sarcomeric proteins and more ubiquitin degradation, which may confound the conclusions. To address this concern, the authors should experimentally should determine if there (a) is a change in titin half life due to increased degradation, (b) is a change in total sarcomere content (i.e. less units of contraction per cardiac myocyte)."

Thank you for raising this point. In our manuscript, we show that cleaved titin is specifically hyper-ubiquitinated, whereas intact titin is not (Fig. S7b, c). Moreover, the persistence of cleaved titin bands – i.e., Cleaved-A (~2.2 MDa) and Cleaved-I fragments (~0.8 and 1.0 MDa) – on gels and blots suggests these large fragments are not immediately cleared by either pathway. However, the pathways governing titin degradation remain incompletely defined, with both the ubiquitin-proteasome system and lysosomal-autophagic machinery implicated. While detailed investigation of titin half-life is beyond the current scope, we agree that future studies tracking titin degradation would yield valuable insights, particularly in the context of chronic cardiac stressors under disease conditions.

As regards the sarcomere content per cardiomyocyte area, we show a modest decrease at D6 which increases by D13 (Fig. S1e, f), and we mention this in the manuscript on p. 5, lines 25-26.

"Minor comments:
1. The use of t-tests in figure 2B-G is inappropriate, and should instead be a two-way ANOVA with multiple comparisons to measure the TEV vs timepoint effects."

We apologize for our oversight and thank the reviewer for the valuable comment. In the revised manuscript, functional data (Fig. 2, Fig. S2h-l, Fig. S3) are now analyzed using two-way ANOVA (mixed-effects model for repeated measures), followed by Šidák's or Tukey's test. In the Statistics section, we describe the statistical analyses applied, including tests for normality, and in all relevant figure legends, we specify the type of statistical test used.

"2. It would be helpful if the subfigures were ordered linearly with their labels."

We thank the reviewer for this suggestion. Subfigures are now ordered linearly with their labels.

Response to Reviewer comments NCVR-2025-09-1424A (final round)

Reviewer #1:

"I have reviewed the revised manuscript and the authors' response to the previous comments.

In my view, the authors have addressed the major concerns in a careful and constructive way. The revised version is clearly improved, particularly regarding the quantitative support of the main findings, the interpretation of fibroblast activation, and the analysis of intercalated-disk changes.

The additional data and clarifications now provide a stronger basis for the proposed sequence of events after titin cleavage. I also appreciate that the discussion has been toned down in several places, which makes the interpretation more balanced and more convincing.

I have no major further comments"

We thank the reviewer for the constructive and helpful critique during the revision of this manuscript.

Reviewer #2:

"The revised manuscript by Freund and coworkers has been significantly improved, starting with a much clearer introduction and ending in a similarly improved discussion. It explains the rationale for cleaving titin in the I-band not far from the physiological T2 site, thereby mimicking the proteolytic titin cleavage observed also in ischemic or chemotherapy-induced cardiac injury and giving insight into I-band truncations in hereditary cardio/myopathies. The results suggest that the precise cleavage location is likely less critical than disruption of the elastic I-band continuity, and it is therefore intriguing that fibrosis sets on just above the natural T1/T2 ratio of about 20%.
The integration of data across scales, from cells to whole animal physiology, remains a major strength of the paper. The single-cardiomyocyte assays showed clearly that titin cleavage reduces resting sarcomere length following rapid relaxation after contraction, which is consistent with the reduced left-ventricular cavity size observed in vivo.

Several important experimental observations have been additionally highlighted, like the fnding that TUNEL-positive cells were fibroblasts, not cardiomyocytes or that the autophagy adapter SQSTM1 was upregulated in Hom, but not Het hearts.

The new expanded section on deregulation of cell-cell connectivity shows that loss of titin stiffness and the resulting force imbalance compromises sarcomere-intercalated disc connectivity,

thus leading to cardiomyocyte detachment, disrupting the mechanical homeostasis sensed by fibroblasts and triggering matrix remodelling.

Insisting on clarifying the domain nomenclature was not malicious capriciousness on behalf of the reviewer. Thank you for clarifying that these are the domains encoded by TTN exons 225 and 226 encoding Ig86 and Ig87, alleviating the confusion in Rivas-Pardo et al. (2020), who were using both I86 and I87 and Ig86 and Ig87, mirroring the nomenclature by Bang et al. (2001) who assigned I to domains presumed to be in the I-band. This numbering is at least 9 domains offset to the contiguous "Ig" nomenclature that avoids such confusion, especially in the differentially spliced regions."

We thank the reviewer for the positive evaluation of our manuscript and insightful comments during the revision process.

Reviewer #3:

"The authors have adequately addressed my concerns. This is a great study and will be impactful for the field."

We thank the reviewer for the guidance during the revision process and positive final evaluation of our manuscript.
